# Naphtho[1,8-*de*][1,2]Oxazin-4-ol: Precursor to 1,2,8-Trisubstituted Naphthalenes and 1-Unsubstituted Naphtho[1,2-*d*]isoxazole 2-Oxide: A Novel Isomerization of the *N*-Oxide to Nitrile Oxide *en Route* to Isoxazol(in)es

**DOI:** 10.3390/molecules29010048

**Published:** 2023-12-20

**Authors:** Ioannis E. Gerontitis, Petros G. Tsoungas, George Varvounis

**Affiliations:** 1 Section of Organic Chemistry and Biochemistry, Department of Chemistry, University of Ioannina, 451 10 Ioannina, Greece; giannhs_ger@yahoo.gr; 2 Department of Biochemistry, Hellenic Pasteur Institute, 127 Vas. Sofias Ave., 115 21 Athens, Greece; pgt@otenet.gr

**Keywords:** naphthols, oxime, oxidation, isomerization, nitrile oxide, 1,3-dipolar cycloaddition, isoxazoles

## Abstract

Naphtho[1,8-*de*][1,2]oxazin-4-ol and its acyl or benzyl derivatives ring open to various 2,8-dihydroxy-1-naphthonitriles, which, through (de)protection protocols and reduction, afford the target (*E*)-2-hydroxy-8-methoxy-1-naphthaldehyde. This was converted to its corresponding oxime, which was oxidatively *o*-cyclized with phenyliodine(III) diacetate (PIDA) to 9-methoxynaphtho[1,2-*d*]isoxazole 2-oxide. The latter, in deuterated DMSO at room temperature, was rearranged to its isomer 2-hydroxy-8-methoxy(naphthalen-1-yl)nitrile oxide. The isomerization was detected by time-course plot ^1^H NMR spectroscopy and further identified from its ^13^C NMR and HRMS spectra. The nitrile oxide was stable in (non)deuterated DMSO for at least 18 h. A 3,4-bis(2-hydroxy-8-methoxynaphthalen-1-yl)-1,2,5-oxadiazole 2-oxide, as a dimerization product or an isocyanate as a rearrangement isomer, was ruled out, the former by its HRMS spectrum and the latter by its 1,3-dipolar cycloaddition reactions to substituted isoxazoles.

## 1. Introduction

1,2-Oxazines and their benzo-fused derivatives are embedded in many drugs and bioactive natural products. Their synthetic strategies, which are used as intermediates in synthesis due to their biological potential, have been comprehensively reported [1,2,3,4,5,6]. Analogues of 1,2-oxazine, *peri-*annelated to naphthalene, are not abundant in the literature. The first naphtho[1,8-*de*][1,2]oxazine to be synthesized was produced by the cyclodehydration of 6,6′,7,7′-tetramethoxygossypol dioxime [7]. Analogous reactions produced more derivatives [8,9]. Varvounis and co-workers oxidized 2-hydroxy-1-naphthaldehyde oxime (**1**) with lead(IV) acetate (LTA) in THF, and obtained, for the first time, naphtho[1,8-*de*][1,2]oxazin-4-ol (**2**) and naphtho[1,2-*d*]isoxazole 2-oxide (**4**); this was a reaction that never gave **4** again but instead gave **2** together with the spiro adduct-dimer **3** [10,11]. Applying the same reaction conditions to (2-hydroxy-1-naphthyl)ketoximes gave the appropriate 3-alkyl-4-hydroxynaphtho[1,8-*de*][1,2]oxazines together with the corresponding 2-alkyl-1-hydroxybenzo[*cd*]indol-3(1*H*)-one side product. (2-Hydroxynaphthalen-1-yl)(phenyl)methanone oxime, on the other hand, gave solely 3-phenyl-4-hydroxynaphtho[1,8-*de*][1,2]oxazine [11]. A high yield of **2** was obtained from the oxidation of **1** with phenyliodonium diacetate (PIDA) in *t*-BuOH [12].

**Figure 1:**
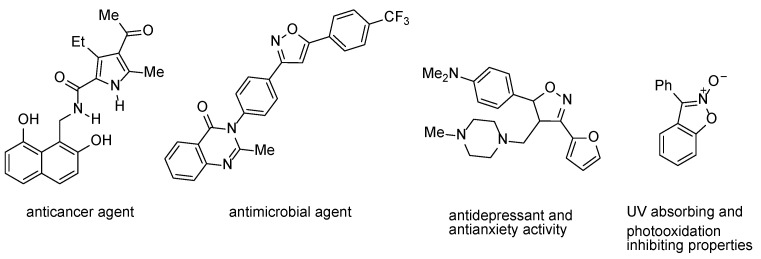
Bioactive and UV-absorbing molecules bearing 1-substituted 2,8-dihydroxynaphthalene, 3,5-diarylisoxazole, 4,5-dihydroisoxazole and benzo[*d*]isoxazole moieties.

Naphthalenediols have applications in dyes, medicines, catalysis, and batteries [13]. For example, the naphthalenediol derivative with an alkylamide arm (Figure 1) is an inhibitor of malignant melanoma, breast cancer, and leukemia [14]. The *ortho*-CH acetoxylation and *peri*-CH hydroxylation [15], as well as the *ortho*- and *peri*-CH methoxylation [16], of substituted 1-naphthaldehydes are important chemical processes for the preparation of useful structures in synthesis and applications.

Isoxazole and 4,5-dihydroisoxazole (isoxazoline) have long been known as privileged core structures of the family of 5-membered aromatic *N*,*O*-heterocycles. Their commonly reported di- and trisubstituted derivatives have shown a wide spectrum of applications in medicinal chemistry, marketed drugs, organic materials, and agriculture, and they are versatile building blocks in organic synthesis. These aspects of isoxazoles and isoxazolines have been extensively reviewed [17,18,19,20,21]. For example, the depicted 3,5-diarylisoxazole derivative (Figure 1) has shown excellent antimicrobial activity against all pathogenic strains [20], while the 3,4,5-trisubstituted 4,5-dihydroisoxazole exhibited antidepressant activity and antianxiety activity [22]. Dialkyl 3-(napththalen-1-yl)isoxazole-4,5-dicarboxylates were obtained by a 1,3-dipolar cycloaddition of a (naphthalene-1-yl)nitrile oxide in situ, with the appropriate dialkyl acetylenedicarboxylate. The reactions differ by the generation mode of the nitrile oxide from the 1-naphthaldehyde oxime, by the use of PIDA [23] and oxone^®^ [24,25,26,27], or by chlorination and then dehydrochlorination [28]. 5-Aryl-3-(naphthalene-1- or 2-yl)isoxazoles have been prepared by the oxidative aromatization of 5-aryl-4,5-dihydro-3-(naphthalene-1 or 2-yl)isoxazoles [29,30]. 3,5-Disubstituted isoxazoles have been assembled by the 1,3-dipolar cycloaddition of appropriate nitrile oxides and arylacetylenes [31,32,33]. There are only two published reports on the synthesis of ethyl 4,5-dihydro-3-(naphthalen-1- or 2-yl)isoxazole-5-carboxylates [34]. Both naphthalen-1- and 2-yl derivatives have been synthesized by [2 + 2 + 1] cycloaddition reactions [35]. 5-Aryl-5-(phenyl or 4-chlorophenyl)-4,5-dihydro-3-(naphthalene-1-yl)isoxazoles [36,37] and a few more that have recently been reported have been synthesized [38,39].

A series of 3-(methyl or phenyl)benzo[*d*]isoxazole-2-oxide derivatives have been studied for their use as novel UV absorbers and photooxidation inhibitors of polystyrene, the most effective being 3-phenylbenzo[*d*]isoxazole 2-oxide (Figure 1) [40]. The first 3-(methyl, ethyl or phenyl)benzo[*d*]isoxazole-2-oxide derivatives were synthesized by Boulton and Tsoungas, using the oxidative cyclization of (2-hydroxy-1-phenyl)ketoximes with LTA or sodium hypochlorite [41,42,43]. The reaction has worked equally efficiently with sodium perborate [44], PIDA [40,45,46], HTIB [47], and NCS [46]. The parent C-3 unsubstituted benzo[*d*]isoxazole-2-oxide and the C-1 unsubstituted naphtho[1,2-*d*]isoxazole 2-oxide (**4**) (vide infra) have so far not been isolated, which is a result of their susceptibility to ring-opening or hydrolysis. In line with stable 3-alkyl benzo[*d*]isoxazole 2-oxides, the oxidative cyclization of (*E*,*Z*)-1-(2-hydroxynaphthalen-1-yl)propan-1-one oxime produced 1-ethylnaphtho[1,2-*d*]isoxazole 2-oxide [11]. The oxidative cyclization of 1-(2-hydroxynaphthalen-1-yl)ethan-1-one oxime with NCS gave stable 1-methylnaphtho[1,2-*d*]isoxazole 2-oxide [46].

Nitrile oxides are highly reactive; they are mostly non-isolable structures, generated in situ from various precursors [48]. In turn, they, as ambiphilic dipoles with a low HOMO-LUMO energy difference [49], serve as precursors to diverse heterocycles, mainly the 5-membered ones [50,51,52]. Relatively few substituted arylnitrile oxides stable enough to be isolable, such as those bearing a bulky *t*-butyl or mesityl group, an alkoxy, and/or a dimethyl acetal group, have been reported [53,54,55,56]. Moreover, several stable (naphthalen-1-yl)nitrile oxide derivatives have been found in the literature [33,53,57,58,59].

**Scheme 1:**
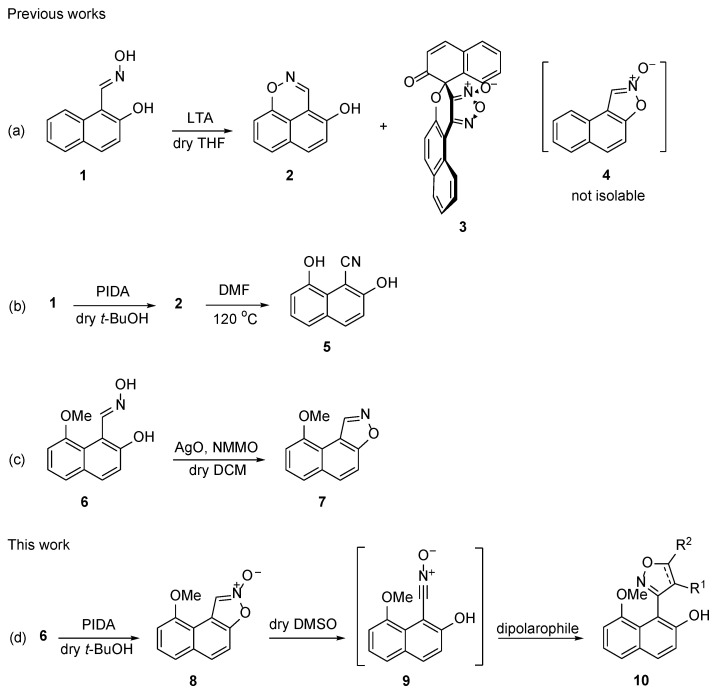
Oxidation of (*E*)-2-hydroxy (or 8-methoxy)-1-naphthaldehyde oximes (**1**) or **6**, ring-opening of 9-methoxynaphtho[1,2-*d*]isoxazole 2-oxide (**8**) in DMSO to the corresponding nitrile oxide **9** and interception with dipolarophiles to afford isoxazole derivatives **10**.

In our previous works, we demonstrated that the oxidation of oxime **1** with LTA, a one-electron oxidant, gave rise to intermediate *o*-naphthoquinone nitrosomethide, which then underwent *peri*-cyclization and intermolecular cyclodimerization to naphtho[1,8-*de*]-[1,2]oxazine (**2**) and (±)-*spiro* adduct-dimer **3**, respectively (Scheme 1a) [10,11]. Later, we established that when **1** was oxidized with PIDA, a two-electron oxidant, in non-nucleophilic *t*-BuOH, oxidative *peri*-cyclization occurred exclusively and provided **2** in an 80% yield [12] (Scheme 1b). More recently, **1** was oxidized by AgO, in the presence of *N*-methyl morpholine *N*-oxide (NMMO), to afford the *spiro* adduct-dimer **3** (Scheme 1a) as the sole product. Under the same reaction conditions, oxime **6** afforded 9-methoxynaphtho[1,2-*d*]isoxazole (**7**), instead of the corresponding *spiro* adduct-dimer [60] (Scheme 1c). We therefore conclude that the formation of the *spiro* adduct-dimer is sterically impeded by the *peri*-OMe substituents.

Herein, we report an optimized synthetic protocol for 2-hydroxy-8-methoxy-1-naphthaldehyde (**14**) from oxazine **2**, which provides access to a variety of 1,2,8-trisubstituted naphthalenes. The transformation of the pivotal aldehyde to oxime **6**; oxidative cyclization to 9-methoxynaphtho[1,2-*d*]isoxazole 2-oxide (**8**); isomerization in DMSO to 2-hydroxy-8-methoxy(naphthalen-1-yl)nitrile oxide (**9**); and the reaction with various dipolarophiles afforded substituted isoxazoles **10** (Scheme 1d).

## 2. Results and Discussion

**Scheme 2:**
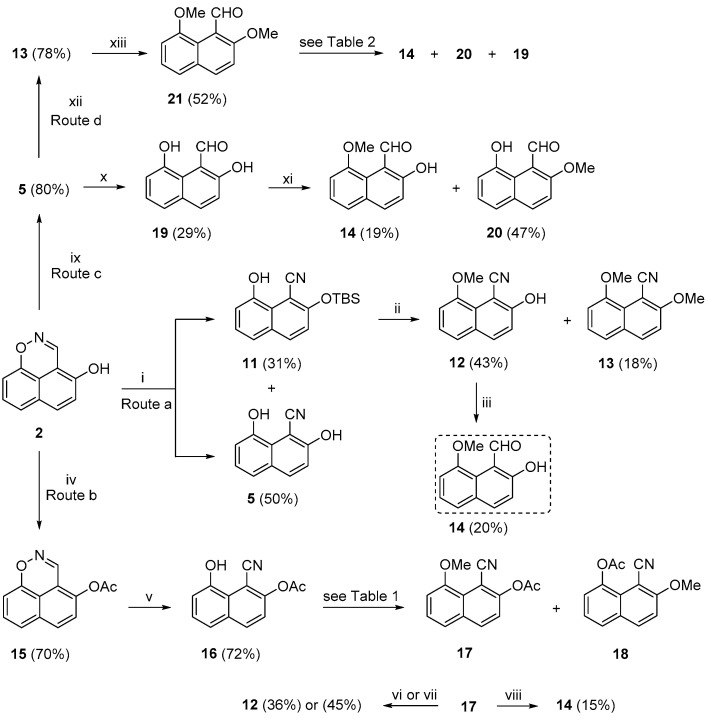
Synthesis of 2-hydroxy-8-methoxy-1-naphthaldehyde (**14**). Reagents and conditions: Route a (i) (**2**) (1 equiv), TBSCl (1.2 equiv), imidazole (2.5 equiv), dry DMF, N_2_, r.t., 2 h then 120 °C, 1 h; (ii) (**11**) (1 equiv), MeI (1.5 equiv), K_2_CO_3_ (1.1 equiv_)_, dry acetone, N_2_, r.t., 1 h; (iii) (**12**) (1 equiv), DIBAL (1.2 M in toluene) (2 equiv), dry toluene, N_2_, 0 °C, 0.5 h, r.t., 18 h. Route b (iv) (**2**) (1 equiv), acetic anhydride (41 equiv), N_2_, r.t., 18 h; (v) (**15**), dry DMF, 120 °C, N_2_, 0.5 h; **16** See Table 1; (vi) (**17**) (1 equiv), DIBAL (1.2 M in toluene) (3.2 equiv), dry THF, NH_4_Cl, 1 M HCl aq., −78 °C, 1 h; (vii) (**17**) (1 equiv), PtO_2_ (0.1 equiv), HCO_2_H/H_2_O, 55–60 °C, 2 h; (viii) (**17**) (1 equiv), DIBAL (1.2 M in toluene) (2 equiv), dry toluene, 0 °C, 0.5 h, r.t., 18 h. Route c (ix) (**2**), dry DMF, 120 °C, N_2_, 0.5 h; (x) (**5**) (1 equiv), Ca(H_2_PO_2_)_2_ (1 equiv), Ni(OAc)_2_·4H_2_O (0.2 equiv), Ca(OAc)_2_·H_2_O (0.2 equiv), EtOH, r.t., 24 h; (xi) (**19**) (1 equiv), MeI (1.05 equiv), K_2_CO_3_ (1.05 equiv), dry DMF, r.t., 2 h. Route d (xii) (**5**) (1 equiv), MeI (2.02 equiv), Na_2_CO_3_ (1.02 equiv), H_2_O, acetone, reflux, 3 h; (xiii) (**13**) (1 equiv), DIBAL (1.2 M in toluene) (2 equiv), dry toluene, 0 °C, 0.5 h, r.t., 18 h; **21** See Table 2.

Following up our previous observations about the instability of unsubstituted naphtho[1,2-*d*]isoxazole 2-oxide (**4**) [11] (Scheme 1a) and the effect of the C-8 OMe group of (*E*)-oxime (**6**) on its AgO (or Ag_2_O) selective oxidation to 9-methoxynaphtho[1,2-*d*]isoxazole (**7**) [60] (Scheme 1c), we investigated the outcome of a PIDA oxidation of that oxime, a reagent used successfully for the oxidative cyclization of 2-hydroxyaryl ketoximes into 3-arylbenzo[*d*]isoxazole 2-oxides [46]. Our recently reported [60] key structure aldehyde **14** (Scheme 2) was prepared by an *o*- and *peri*-methoxylation of 1-naphthaldehyde with Pd(OAc)_2_, K_2_S_2_O_8_, and 3-(trifluoromethyl)aniline in a closed vessel to 2,8-dimethoxy-1-naphthaldehyde in a 30% yield and was further selectively demethylated by AlCl_3_ to **14** in a 15% yield. The low overall yield (4.5%) and the use of a sealed vessel steered us towards an alternative route to **14**. The high-yielding (80%) synthesis of **2**, followed by its equally high-yielding (80%) ring-opening to **5** (Scheme 1b), provided relevant and attractive starting materials.

Thus, compound **2** was reacted with TBSCl and imidazole in dry DMF at room temperature (Scheme 2, Route a), following a relevant report by Bencivenni and co-workers [61], then heated at 120 °C for a short period of time, under the conditions used in our previous report (Scheme 1b) [10]. The expected ring-opened 2-TBS protected 1-naphthonitrile **11** and the unprotected 1-naphthonitrile **5** were produced in 31% and 50% yields, respectively. Compound **5** most likely resulted from **11** by the cleavage of its TBS group. The methylation of **11** (MeI and K_2_CO_3_ in dry acetone), under the conditions used by Bencivenni and co-workers [61] on 7-[(*tert*-butyldimethylsilyl)oxy]naphthalen-1-ol, furnished the selectively methylated nitrile **12** in a 43% yield and dimethylated nitrile **13** in an 18% yield. Nitrile **12** was then reduced to aldehyde **14** with DIBAL in toluene at 0 °C [62] and then at room temperature, in our case. The overall yield of **14** from **2**, as described in Scheme 2, was only 2.8%.

In our next attempt (Scheme 2, Route b), we acetylated **2** with acetic anhydride, according to an old, published procedure [8], and obtained acetate **15** in a 70% yield. The ring-opening of **15** in DMF at 120 °C, under conditions used previously [10], afforded 1-naphthonitrile **16** in a 72% yield. We then conducted a search for methylation of the OH group of **16** without cleaving the acetyl group (Table 1). For a detailed description of the procedures, see Materials and Methods (Section 3) [63]. The origin of **18** may well have been derived from an intermolecular acetylation between two molecules of **16**, followed by the methylation of the resulting 8-acetoxy-1-cyanonaphthalen-2-olate. The methylation outcome of **16** remained the same, under different conditions but with varying yields of **17** and **18**. It was interesting to note that the solvent effect in the last two attempts apparently favored **17** (41% yield) compared to **18** (9% yield). Moreover, the OMe group in **17** was properly placed for further deprotection and reduction to the target **16**. Thus, by reducing **17** with either DIBAL in dry THF at −78 °C [62] or with PtO_2_ in an equal volume of HCO_2_H/H_2_O at 55–60 °C [64], **12** was obtained in 36% and 45% yields, respectively. The reduction of **17** to **14** proved difficult, under either the standard DIBAL conditions [62] or an attempted modification (initiating the reaction at 0 °C and allowing a long period at room temperature), and gave a disappointing 15% yield and an overall 3% yield from **2**.

In our next attempt (Scheme 2, Route c), we anticipated that the 6-membered intramolecular H bond in aldehyde **19** between the CHO oxygen atom and the OH hydrogen atom would be strong enough to survive in DMF and would thus encourage selective methylation at the *peri* position. Thus, the ring-opening of **2** to **5** [10] and the reduction of the CN group in **5** with calcium hypophosphite, in the presence of a base and nickel(II) acetate tetrahydrate, according to Estelle Métay, Marc Lemaire, and co-workers [65], who reduced 1-naphthonitrile to 1-naphthaldehyde in an 85% yield, led to a disappointing 29% yield of **19**. Aryl nitriles bearing OH groups are tolerant to these conditions. The methylation of **19** (dry DMF and at room temperature, in the presence of MeI and K_2_CO_3_) afforded the targets **14** and **20** in 19% and 47% yields, respectively [15]. The formation of **20** implies that in a DMF solution of **19**, the intramolecular H bond between the CHO group and the *peri*-OH group survives, whereas that between the CHO group and the *ortho* OH group is disrupted by intermolecular H bonding with the solvent. The outcome of this effort was a 4% overall yield of **6**, which was more or less the same as in Scheme 2, Route b.

The next plan was to alkylate **5**, reduce the resultant nitrile **13** to the corresponding aldehyde **21**, and then selectively demethylate to the target **14** (Scheme 2, Route d). Accordingly, the dimethylation of **5** (MeI and Na_2_CO_3,_ in aqueous acetone, under mild heating) [61], gave **13** in a satisfying 78% yield. The latter was reduced to the aldehyde **21** in a moderate yield by a modification of the standard DIBAL conditions [62], as described earlier. Aldehyde **21** was then subjected to what we hoped would be the selective deprotection of the 2-OMe group to the target **14** in a useful yield. Thus, the selective demethylation of **21** to **14** entailed various reagents and conditions, as depicted in Table 2. For a detailed description of the procedures, see Materials and Methods (Section 3) [66,67]. We can assume that at low temperature, the *peri*-OMe group in **21** is intramolecularly engaged in a H bond with the CHO group for most of the time. Consequently, this H bonding interaction allows a relatively easier demethylation of its more available *o*-OMe counterpart. The moderate yields of the last two steps of this reaction sequence towards **14** diminished its value, despite an increase in the overall yield to 18%.

**Scheme 3:**
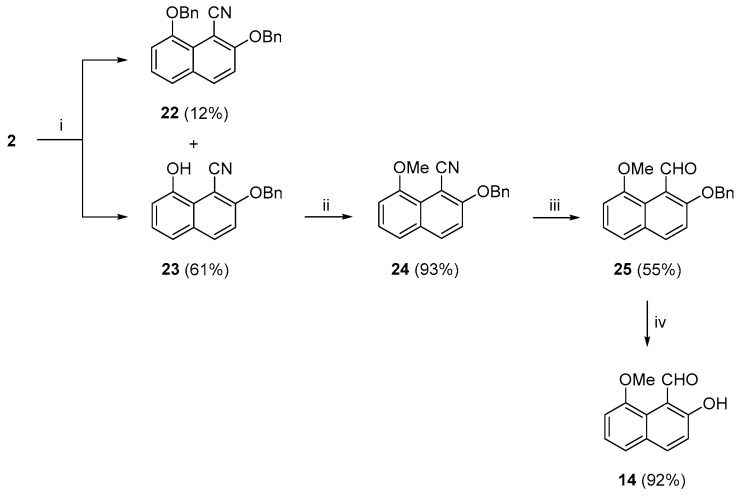
Synthesis of 2-hydroxy-8-methoxy-1-naphthaldehyde (**14**) via benzylation/ring-opening/methylation/reduction/debenzylation. Reagents and conditions: (i) **2** (1 equiv), BnBr (1 equiv), K_2_CO_3_ (1.05 equiv), KI (0.2 equiv), dry acetone, r.t., 18 h; (ii) **23** (1 equiv), MeI (1.02 equiv), Na_2_CO_3_ (1.02 equiv), dry acetone, N_2_, reflux, 3 h; (iii) **24** (1 equiv), DIBAL (1.2 M in toluene) (2 equiv), dry toluene, 0 °C, 0.5 h, r.t., 18 h; (iv) 5% Pd/C, H_2_, MeOH, r.t., 12 h.

The final attempt to synthesize **14** is delineated in Scheme 3. Accordingly, we started with an *O*-benzylation of **2** and then a ring-opening of the resulting 4-(benzyloxy)naphtho[1,8-*de*][1,2]oxazine (by heating in DMF) to the target precursor **23**. Further, we envisaged the methylation of the *peri*-OH group, the reduction of nitrile to aldehyde, and the debenzylation of the OBn group. For the benzylation step, we slightly modified the conditions applied by Luo and Zheng and co-workers [68] to convert 2-hydroxy-1-naphthaldehyde to 2-(benzyloxy)-1-naphthaldehyde. Starting from **2,** we used benzyl bromide, instead of benzyl chloride, in the presence of K_2_CO_3_ and KI and stirred the reaction in acetone at room temperature for 18 h. Compounds **22** and **23** were identified in 12% and 61% yields, respectively. To our surprise, the intermediate 4-(benzyloxy)naphtho[1,8-*de*][1,2]oxazine was not detected. Apparently, the oxazine suffered ring-opening in the presence of the base. Indeed, this result was experimentally verified by stirring **2** with K_2_CO_3_ and KI in acetone, and after 4 h, **5** was obtained as a single product. It may be argued that benzylation could take place either sequentially, first on **2** and then on ring-opened **22** to **23**, or on the ring-opened **5**. The higher yield of **23** points to the former process. In the second step of this reaction sequence, **23** was methylated (MeI and K_2_CO_3_ in dry acetone under reflux) [61] to afford the 8-OMe derivative **24** in an excellent 94% yield. The reduction of the nitrile group of **24** was accomplished under conditions described earlier [62], and aldehyde **25** was thus obtained in a moderate 55% yield. The debenzylation of **25** took place with H_2_ and Pd/C as catalysts, according to an analogously reported procedure [69] that gave **14** in an excellent 92% yield. The overall yield of **14** from **2** by this route (Scheme 3) was increased to 29%.

**Scheme 4:**
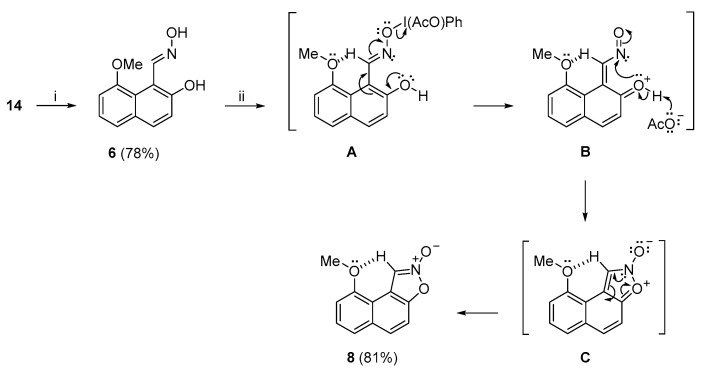
Synthesis of (*E*)-2-hydroxy-8-methoxy-1-naphthaldehyde oxime (**6**) and proposed reaction mechanism for the synthesis of 9-methoxynaphtho[1,2-*d*]isoxazole 2-oxide (**8**). Reagents and conditions: (i) 14 (1 equiv), NH_2_OH.HCl (1.1 equiv), aqueous saturated Na_2_CO_3_ solution, MeOH, 0 °C, r.t., 1 h; (ii) 6 PIDA (2 equiv), dry *t*-BuOH, N_2_, r.t., 0.5 h.

Having established a viable route to target **14**, we moved to the synthesis of (*E*)-oxime **6** and its reaction with PIDA (Scheme 4). Compound **6** was accordingly obtained in a 78% yield by a method in a recent report by Tzeli, Tsoungas, and co-workers [60]. Compound **6** was then subjected to oxidation with PIDA in non-nucleophilic dry *t*-BuOH at room temperature, to afford isoxazole-2-oxide **8** in a very good yield (81%). We propose that the initially formed organoiodo complex **A** by the ligand coupling mechanism [70] collapses to *o*-naphthoquinone nitrosomethide intermediate **B**; the driving force is the rupture of the weaker O–I bond compared to the N–O bond. **B** underwent a 6π-electrocyclization to the non-aromatic naphthoisoxazole-2-oxide **C,** which aromatized to stable 2-oxide **8**. The stability of **8** was apparently due to the intramolecular H bonding between the OMe *peri* substituent and the sp^2^ C–1 hydrogen atom (2.006 Å) [60], which impeded the ring-opening of the isoxazole ring. This result could well serve as a rationale for the non-isolable naphtho[1,2-*d*]isoxazole 2-oxide (**4**), which lacks this particular stabilizing factor (Scheme 1a) [11].

**Scheme 5:**
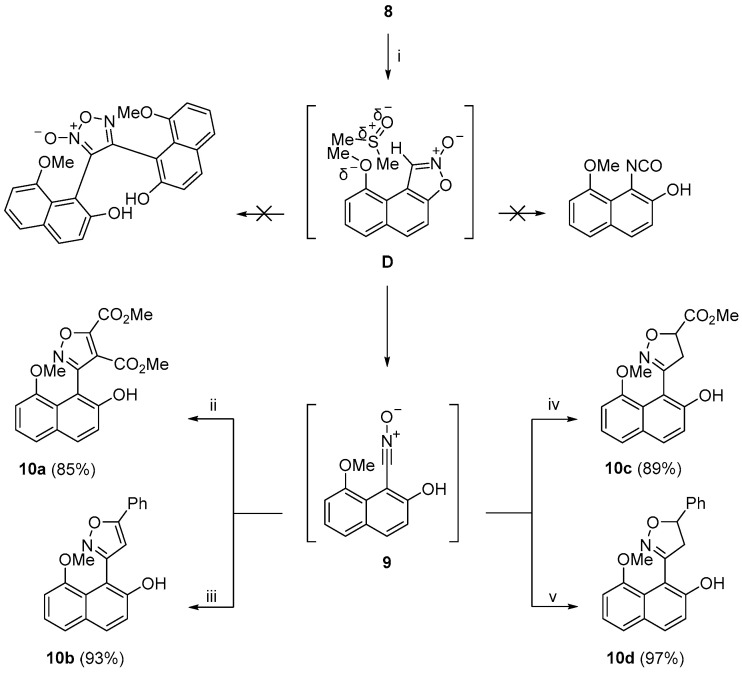
Synthesis of substituted isoxazoles **10a** to **10d**. Reagents and conditions: (i) **8** (1 equiv) dry DMSO-*d*_6_, N_2_, r.t., 15 min.; (ii) DMAD (3 equiv), 18 h; (iii) phenylacetylene (3 equiv), 18 h; (iv) methyl acrylate (3 equiv), 18 h; (v) styrene (3 equiv), 18 h.

When first recorded, the ^1^H NMR spectrum of **8** in DMSO-*d*_6_ did show the peaks corresponding to its structure. When recorded again the next day, its ^1^H NMR spectrum was identified as that of the nitrile oxide **9** (see Supplementary Figure S59 for superimposed and Figure S60 for stacked ^1^H NMR spectra of **8** and **9**). This novel isomerization of **8** to **9** was also detected by ^1^H NMR spectroscopy, which was measured by a time-course plot (see Supplementary Figure S61). When recorded in CDCl_3_, the ^1^H and ^13^C NMR spectra of **8** confirmed that the compound is stable in this solvent for at least 18 h. We stirred a sample of **8** in DMSO-*d*_6_ and after 6 h recorded the ^1^H and ^13^C NMR spectra. As with the previously recorded NMR spectra of nitrile oxide **9**, in the ^1^H NMR spectrum in DMSO-*d*_6_, the OH proton at a high field was not visible, and there was a total of five aromatic protons belonging to the naphthalen-2-ol ring in the range of 7.90–7.02 ppm. The characteristic singlet of the methyl group was found at 3.92 ppm. The ^13^C NMR spectrum in DMSO-*d*_6_ showed 11 signals. According to Koyama, Takata, and co-workers, [71] quaternary signals of nitrile groups usually turn up under the residual DMSO signal so that for nitrile oxide **9** the total number of carbon atoms was 12, as expected. High-resolution mass spectrometry (HRMS) analysis confirmed the expected molecular ion at *m*/*z* = 216.0661 [M + H]^+^ (ESI), which took up a proton and was calculated for C_12_H_10_NO_3_ *m*/*z* = 216.0661. There was no peak at *m*/*z* = 431.1243 [M + H]^+^ corresponding to the dimerization of **9** to its 1,2,5-oxadiazole 2-oxide (Scheme 5). The confirmation of the nitrile oxide structure of **9** and not its isocyanate isomer came from 1,3-dipolar cycloaddition reactions of in situ generated nitrile oxide **9** with various dipolarophiles, which produced substituted isoxazoles **10a**–**d** (Scheme 5). The reactions took place by dissolving isoxazole 2-oxide **8** in DMSO, under an atmosphere of nitrogen, followed by the addition of the appropriate dipolarophile (DMAD, phenylacetylene, methyl acrylate, or styrene, stirred at room temperature for 18 h). The substituted isoxazoles **10a**–**d** were produced in 85%, 93%, 89%, and 97% yields, respectively. It was suggested that when 2-oxide **8** was dissolved in DMSO, the solvated species **D** could suffer disruption of the intramolecular H bond between the MeO group and the C-1 hydrogen atom, allowing for the ring-opening of the isoxazole ring to its nitrile oxide isomer **9**, which then underwent 1,3-dipolar cycloaddition with the dipolarophiles (Scheme 5).

## 3. Materials and Methods

The organic solutions were concentrated by rotary evaporation at 40 °C under 15 Torr. Melting points were taken using a Büchi 510 apparatus (Büchi Labortechnik AG, Flawil, Switzerland) and are uncorrected. ^1^H and ^13^C NMR spectra were measured in CDCl_3_ or DMSO-*d*_6_ on a 400 MHz Brüker Avance spectrometer (Brüker BioSpin GmbH, Rheinstetten, Germany). ^1^H chemical shifts are reported in ppm from an internal standard TMS, residual CHCl_3_ (7.26 ppm), or DMSO (2.50 ppm). ^13^C NMR chemical shifts are reported in ppm from an internal standard TMS, residual CHCl_3_ (77.00 ppm), or DMSO (39.43 ppm). High resolution ESI mass spectra were measured on a Thermo Fisher Scientific Orbitrap XL system (Thermo Fisher Scientific, Waltham, MA, USA). IR spectra were acquired on an Agilent Cary 630 FTIR spectrophotometer (Agilent Technologies, 5301 Stevens Creek Blvd. CA, USA) as solids and are reported in wave numbers (cm^−1^). Analytical thin layer chromatography (TLC) was performed with TLC plates (Merck 70–230 mesh silica gel). TLC visualization took place under a 254 nm UV light source. Purification of the reaction products was generally conducted by flash column chromatography using Carlo Erba Reactifs-SDS silica gel 60. Solvents, reagents, and catalysts were used as received from the manufacturers, Thermo Scientific™ (Paisely, U.K.), Sigma-Aldrich (St. Louis, MO, USA), and Merck Chemicals GmbH (Darmstadt, Germany), except for DCM, EtOAc, and hexane, which were dried and purified according to the recommended procedures.

### 3.1. Synthesis of 2-[(Tert-butyldimethylsilyl)oxy]-8-hydroxy-1-naphthonitrile (***11***) and 2,8-Dihydroxy-1-naphthonitrile (***5***)

To a solution of compound **2** (500 mg, 2.2 mmol, 1 equiv) in dry DMF (15 mL), under an atmosphere of N_2_, was added imidazole (374 mg, 5.5 mmol, 2.5 equiv) and TBSCl (398 mg, 2.64 mmol, 1.2 equiv), and the reaction was left stirring at room temperature for 2 h (TLC analysis showed the absence of the starting material spot and the presence of a new spot). The reaction mixture was then heated at 120 °C for 0.5 h. TLC examination revealed the absence of the starting material spot and the presence of two new spots. To the cooled reaction mixture, water (100 mL) was added and extracted with EtOAc (3 × 20 mL), and the combined organic extracts were washed with brine (20 mL), dried over anhydrous Na_2_SO_4_, and concentrated in a vacuum. The acquired crude residue was purified by flash column chromatography (25% EtOAc in hexane) to give the title compounds.

Compound **11**: (204 mg, 31%) as a yellow oil; R*_f_* = 0.61 (25% EtOAc in hexane); ^1^H NMR (400 MHz, CDCl_3_) *δ*: a broad singlet corresponding to OH is not visible, 7.76 (d, *J =* 8.9 Hz, 1H), 7.26 (d, *J =* 8.2 Hz, 1H), 7.18 (t, *J =* 7.5 Hz, 1H), 7.00–6.90 (m, 2H), 1.01 (s, 9H), 0.25 (s, 6H); ^13^C NMR (100.6 MHz, CDCl_3_) *δ*: 160.0, 150.9, 134.9, 130.4, 125.8, 123.7, 121.1, 120.3, 118.7, 113.2, 95.2, 25.8, 18.5; IR (solid): 3161, 3060, 2955, 2922, 2855, 2215, 2118, 1729 cm^−1^; HRMS (ESI): *m*/*z* [M–H]^−^ calcd. for C_17_H_21_NO_2_Si: 298.1263, found: 298.1264.
Compound **5**: (203 mg, 50%) as a yellow solid, m.p. = 166–167 °C (lit. [10], m.p. = 167–168 °C); R*_f_* = 0.1 (20% ethyl acetate in hexane); ^1^H NMR (400 MHz, DMSO-*d*_6_) *δ*: two broad singlets corresponding to OH are not visible, 11.24 (s, 1H), 10.31 (s, 1H), 8.20 (d, *J =* 8.7 Hz, 1H), 7.66 (d, *J =* 8.3 Hz, 1H), 7.42 (t, *J* = 7.8 Hz, 1H), 7.34 (d, *J* = 8.7 Hz, 1H), 7.25 (d, *J* = 7.3 Hz, 1H) (in agreement with the ^1^H NMR data that were previously reported for this compound) [10].

### 3.2. Synthesis of 2-Hydroxy-8-methoxy-1-naphthonitrile (***12***) and 2,8-Dimethoxy-1-naphthonitrile (***13***)

To a solution of compound **2** (100 mg, 0.334 mmol, 1 equiv) in dry acetone (10 mL), under an atmosphere of N_2_, was added oven-dried K_2_CO_3_ (50 mg, 0.367 mmol, 1.1 equiv) and MeI (70 mg, 0.501 mmol, 1.5 equiv), and the reaction was left stirring at room temperature for 1 h (TLC analysis showed complete conversion of the starting material and the presence of two new spots). Water (20 mL) was added, the reaction mixture was extracted with EtOAc (3 × 10 mL), and the combined organic extracts were washed with brine (10 mL), dried over anhydrous Na_2_SO_4_, and concentrated under vacuum. The acquired crude residue was purified by flash column chromatography (11% EtOAc in hexane) to give the title compounds.

Compound **12**: (28.6 mg, 43%) as a yellow solid, m.p. = 172–174 °C; R*_f_* = 0.09 (20% EtOAc in hexane); ^1^H NMR (400 MHz, CDCl_3_) *δ*: a broad singlet corresponding to OH is not visible, 7.90 (d, J = 9.0 Hz, 1H), 7.42–7.33 (m, 2H), 7.22 (d, J = 9.0 Hz, 1H), 6.97 (d, *J =* 7.3 Hz, 1H), 4.05 (s, 3H); ^13^C NMR (100.6 MHz, CDCl_3_) *δ*: 160.4, 153.9, 135.2, 129.8, 125.5, 123.6, 121.2, 117.5, 117.4, 107.9, 89.4, 55.9; IR (solid): 3072, 2920, 2848, 2216, 1705, 1600, 1513 cm^−1^; HRMS (ESI): *m*/*z* [M + Na]^+^ calcd. for C_12_H_9_NO_3_Na: 222.0531, found: 222.0528.
Compound **13**: (12.8 mg, 18%) as a colorless amorphous solid (hexane), m.p. = 147–149 °C; R*_f_* = 0.21 (20% EtOAc in hexane); ^1^H NMR (400 MHz, CDCl_3_) *δ*: 7.83 (d, *J =* 9.2 Hz, 1H), 7.25 (d, *J =* 8.2 Hz, 1H), 7.19 (t, *J =* 7.9 Hz, 1H), 7.13 (d, *J =* 9.3 Hz, 1H), 6.80 (dd, *J =* 7.5, 1.2 Hz, 1H), 3.93 (s, 3H), 3.89 (s, 3H); ^13^C NMR (100.6 MHz, CDCl_3_) *δ*: 162.9, 154.3, 134.8, 129.7, 125.2, 123.8, 121.1, 117.0, 112.4, 107.7, 92.6, 56.8, 55.8; IR (solid): 2921, 2848, 2218, 2091, 1915, 1826, 1593 cm^−1^; HRMS (ESI): *m*/*z* [M + H]^+^ calcd. for C_13_H_12_NO_2_: 214.0863, found: 214.0858.

### 3.3. Synthesis of 2-Hydroxy-8-methoxy-1-naphthaldehyde (***14***)

To a stirred solution of **12** (200 mg, 1 mmol, 1 equiv) in dry toluene (10 mL), under an atmosphere of N_2_ at 0 °C, was added DIBAL (1.67 mL of 1.2 M in toluene, 2 mmol, 2 equiv), and the reaction mixture was left stirring for 0.5 h and then for 18 h at room temperature (TLC analysis showed the absence of a starting material spot and the presence of a new spot). The solvent was removed under reduced pressure, water (20 mL) was carefully added to the residue, and the resulting mixture was cooled to 0 °C, followed by the dropwise addition of 1 M aqueous HCl until pH = 1. The aqueous solution was extracted with EtOAc (3 × 10 mL), and the combined organic extracts were washed with brine (20 mL), dried over anhydrous Na_2_SO_4_, and concentrated under vacuum. The acquired crude residue was purified by flash column chromatography (17% EtOAc in hexane) to give the title compound (40 mg, 20%) as a yellow solid, m.p. = 66–69 °C (lit. [60], m.p. = 68–70 °C); R*_f_* = 0.54 (20% ethyl acetate in hexane); ^1^H NMR (400 MHz, CDCl_3_) *δ*: 14.15 (s, 1H), 11.22 (s, 1H), 7.87 (d, *J =* 9.0 Hz, 1H), 7.38 (dd, *J =* 8.0, 1.3 Hz, 1H), 7.31 (t, *J =* 7.9 Hz, 1H), 7.10 (d, *J =* 9.0 Hz, 1H), 7.04 (dd, *J =* 7.8, 1.2 Hz, 1H), 3.99 (s, 3H) (in agreement with the ^1^H NMR data that were previously reported for this compound) [60].

### 3.4. Synthesis of Naphtho[1,8-de][1,2]oxazin-4-yl Acetate (***15***)

A solution of **2** (500 mg, 2.7 mmol, 1 equiv) in freshly distilled acetic anhydride (10 mL, 41 equiv), under an atmosphere of N_2_, was stirred at room temperature for 18 h (TLC showed the absence of a starting material spot and the presence of one new spot). Ice water (30 mL) was added, and the reaction mixture was stirred for 0.5 h at room temperature. The reaction mixture was then extracted with EtOAc (3 × 15 mL), and the combined organic extracts were washed with brine (10 mL), dried over anhydrous Na_2_SO_4_, and concentrated under vacuum. The acquired crude residue was purified by flash column chromatography (17% EtOAc in hexane) to give the title compound (429 mg, 70%) as a brown oil; R*_f_* = 0.05 (20% EtOAc in hexane); ^1^H NMR (400 MHz, DMSO-*d*_6_) *δ*: 8.71 (s, 1H), 7.99 (d, *J =* 9.1 Hz, 1H), 7.58–7.51 (m, 2H), 7.41 (d, *J =* 9.1 Hz, 1H), 7.06–6.97 (m, 1H), 2.33 (s, 3H); ^13^C NMR (100.6 MHz, DMSO-*d_6_*) *δ*: 169.3, 167.0, 151.3, 144.2, 140.4, 130.8, 128.3, 124.1, 119.9, 119.2, 107.9, 105.7, 22.0; IR (solid): 2924, 2682, 2217, 1926, 1749, 1510 cm^−1^; HRMS (ESI): *m*/*z* [M + Na]^+^ calcd. for C_13_H_9_NO_3_Na: 250.0480, found: 250.0477.

### 3.5. Synthesis of 1-Cyano-8-hydroxynaphthalen-2-yl Acetate (***16***)

A solution of **15** (400 mg, 2.16 mmol) in DMF (8 mL) was heated at 120 °C for 45 min. (TLC analysis showed the absence of a starting material spot and the presence of a new spot). Water (30 mL) was added, and the reaction mixture was extracted with Et_2_O (3 × 15 mL). The combined organic extracts were dried (Na_2_SO_4_), and the solvent was removed under vacuum. The residue was purified by flash column chromatography (50% EtOAc in hexane) to give the title compound (267 mg, 72%) as a brown solid; m.p. = 165–167 °C; R*_f_* = 0.13 (20% ethyl acetate in hexane); ^1^H NMR (400 MHz, DMSO-*d*_6_) *δ*: 10.85 (s, 1H), 8.22 (d, *J =* 9.0 Hz, 1H), 7.55–7.42 (m, 3H), 7.07 (dd, *J =* 7.5, 1.3 Hz, 1H), 2.41 (s, 3H); ^13^C NMR (100.6 MHz, DMSO-*d*_6_) *δ*: 168.7, 153.8, 152.4, 134.8, 132.69, 127.9, 122.2, 121.7, 119.4 (2C), 115.4, 111.7, 99.0, 20.7; IR (solid): 3338, 2921, 2855, 2229, 2070, 1906, 1753, 1583 cm^−1^; HRMS (ESI): *m*/*z* [M + Na]^+^ calcd. for C_13_H_9_NO_3_Na: 250.0480, found: 250.0471.

### 3.6. Procedure A for the Synthesis of 1-Cyano-8-methoxynaphthalen-2-yl Acetate (***17***) and 8-Cyano-7-methoxynaphthalen-1-yl Acetate (***18***)

To a solution of **16** (50 mg, 0.22 mmol, 1 equiv) in dry acetone (5 mL), under an atmosphere of N_2_, was added oven-dried K_2_CO_3_ (34 mg, 0.24 mmol, 1.1 equiv) and dimethyl sulfate (31 mg, 0.24 mmol, 1.1 equiv), and the reaction was left stirring for 24 h at room temperature. (TLC showed complete conversion of the starting material and the presence of two new spots (visualized under a UV lamp). Water (20 mL) was added; the reaction mixture was extracted with EtOAc (3 × 10 mL); and the combined organic extracts were washed with brine (10 mL), dried over anhydrous Na_2_SO_4_, and concentrated in vacuo. The acquired crude residue was purified by flash column chromatography (20% EtOAc in hexane) to give the title compounds.

Compound **17**: (5.3 mg, 10%) as a yellow solid, m.p. = 94–96 °C; R*_f_* = 0.25 (20% EtOAc in hexane); ^1^H NMR (400 MHz, DMSO-*d*_6_) *δ*: 8.30 (d, *J =* 8.9 Hz, 1H), 7.66 (dd, *J =* 8.3, 1.2 Hz, 1H), 7.64–7.52 (m, 2H), 7.24 (dd, *J =* 7.8, 1.1 Hz, 1H), 4.00 (s, 3H), 2.42 (s, 3H); ^13^C NMR (100.6 MHz, DMSO-*d*_6_) *δ*: 168.6, 154.2, 153.8, 135.0, 132.2, 127.8, 122.8, 122.1, 121.1, 115.2, 108.5, 98.8, 56.0, 20.6; IR (solid): 2924, 2855, 2223, 1578 cm^−1^; HRMS (ESI): *m*/*z* [M + H]^+^ calcd. for C_14_H_12_NO_3_: 242.0812, found: 242.0813.
Compound **18**: (26.5 mg, 50%) as a yellow solid, m.p. = 132–134 °C; R*_f_* = 0.11 (20% EtOAc in hexane); ^1^H NMR (400 MHz, DMSO-*d*_6_) *δ*: 8.36 (d, *J =* 9.3 Hz, 1H), 7.95 (dd, *J =* 8.2, 1.3 Hz, 1H), 7.66 (d, *J =* 9.3 Hz, 1H), 7.54–7.50 (m, 1H), 7.43 (dd, *J =* 7.6, 1.2 Hz, 1H), 4.08 (s, 3H), 2.42 (s, 3H); ^13^C NMR (100.6 MHz, DMSO-*d*_6_) *δ*: 169.9, 163.7, 144.0, 136.2, 129.3, 127.4, 124.9 (2C), 123.0, 116.1, 113.7, 88.912, 57.1, 20.9; IR (solid): 2942, 2850, 2219, 2101, 1747, 1592 cm^−1^; HRMS (ESI): *m*/*z* [M + Na]^+^ calcd. for C_14_H_11_NO_3_Na: 264.0631, found: 264.0625.

### 3.7. Procedure B for the Synthesis of Compounds ***17*** and ***18***

To a solution of **8** (50 mg, 0.22 mmol, 1 equiv) in dry THF (5 mL), under an atmosphere of nitrogen, was added oven-dried K_2_CO_3_ (34 mg, 0.24 mmol, 1.1 equiv) and MeI (34 mg, 0.24 mmol, 1.1 equiv), and the reaction mixture was left stirring for 18 h at room temperature (TLC showed complete conversion of the starting material and the presence of two new spots). Water (20 mL) was added, and the reaction mixture was extracted with EtOAc (3 × 10 mL); the combined organic extracts were washed with brine (10 mL), dried over anhydrous Na_2_SO_4_, and concentrated under reduced pressure. The acquired crude residue was purified by flash column chromatography (20% EtOAc in hexane) to give the title compounds.

Compound **17**: (6.9 mg, 13%) as a yellow solid, m.p. = 94–96 °C; R*_f_* = 0.25 (20% EtOAc in hexane); (the ^1^H NMR data were in agreement with those reported in Procedure A for this compound).
Compound **18**: (25 mg, 50%) as a yellow solid, m.p. = 132–134 °C; R*_f_* = 0.11 (20% EtOAc in hexane); (the ^1^H NMR data were in agreement with those reported in Procedure A for this compound).

### 3.8. Procedure C for the Synthesis of Compounds ***17*** and ***18***

To a solution of **16** (50 mg, 0.22 mmol, 1 equiv) in dry THF (5 mL), under an atmosphere of N_2_, was added NaH (5.8 mg, 0.24 mmol, 1.1 equiv) and MeI (34 mg, 0.24 mmol, 1.1 equiv), and the reaction mixture was left stirring for 18 h at room temperature (TLC analysis showed the absence of starting material and the presence of two new spots). Water (20 mL) was added to the reaction mixture and then extracted with EtOAc (3 × 10 mL). The combined organic extracts were washed with brine (10 mL), dried over anhydrous Na_2_SO_4_, and removed under reduced pressure. The acquired crude residue was purified by flash column chromatography (20% EtOAc in hexane) to give the title compounds.

Compound **17**: (11.7 mg, 22%) as a yellow solid, m.p. = 94–96 °C; R*_f_* = 0.25 (20% EtOAc in hexane); (the ^1^H NMR data were in agreement with those reported in Procedure A for this compound).
Compound **18**: (21 mg, 40%) as a yellow solid, m.p. = 132–134 °C; R*_f_* = 0.11 (20% EtOAc in hexane); (the ^1^H NMR data were in agreement with those reported in Procedure A for this compound).

### 3.9. Procedure D for the Synthesis of Compounds ***17*** and ***18***

To a solution of **16** (50 mg, 0.22 mmol, 1 equiv) in dry THF (50 mL), under an atmosphere of N_2_, was added NaH (5.8 mg, 0.24 mmol, 1.1 equiv) and MeI (34 mg, 0.24 mmol, 1.1 equiv), and the reaction was left stirring for 18 h at room temperature (TLC analysis showed the absence of the starting material spot and the presence of two new spots). Water (20 mL) was added to the reaction mixture and extracted with EtOAc (3 × 10 mL); the combined organic extracts were washed with brine (10 mL), dried over anhydrous Na_2_SO_4_, and evaporated under vacuum. The acquired crude residue was purified by flash column chromatography (20% EtOAc in hexane) to give the title compounds.

Compound **17**: (21.8 mg, 41%) as a yellow solid, m.p. = 94–96 °C; R*_f_* = 0.25 (20% EtOAc in hexane); (the ^1^H NMR data were in agreement with those reported in Procedure A for this compound).
Compound **18**: (4.8 mg, 9%) as a yellow solid, m.p. = 132–134 °C; R*_f_* = 0.11 (20% EtOAc in hexane); (the ^1^H NMR data were in agreement with those reported in Procedure A for this compound).

### 3.10. Attempted Reduction of 1-Cyano-8-methoxynaphthalen-2-yl Acetate (***17***) with DIBAL

To a solution of **17** (50 mg, 0.27 mmol, 1 equiv) in dry THF, under an atmosphere of N_2_ that was cooled to −78 °C, DIBAL (0.86 mL of 1.2 M in toluene, 0.864 mmol, 3.2 equiv) was added dropwise, and the reaction mixture was left stirring at that temperature for 0.5 h and then for 1 h at room temperature (TLC analysis showed the absence of the starting material spot and the presence of one new spot). A saturated aq. solution of NH_4_Cl (5 mL) was added dropwise, followed by 1 N HCl aq. (25 mL) and EtOAc (25 mL), and the reaction mixture was left stirring for 1 h. The organic phase was separated, and the aqueous phase was extracted with EtOAc (3 × 10 mL); the combined organic phases were washed with brine (20 mL), dried over anhydrous Na_2_SO_4_, and evaporated under vacuum. The acquired crude residue was purified by flash column chromatography (20% EtOAc in hexane) to give 2-hydroxy-8-methoxy-1-naphthonitrile (**12**) (23 mg, 36%) as a yellow solid, m.p. = 172–174 °C; R*_f_* = 0.09 (20% EtOAc in hexane); (the ^1^H NMR data were in agreement with those reported for this compound synthesized from compound **2** (Scheme 2)).

### 3.11. Attempted Reduction of 1-Cyano-8-methoxynaphthalen-2-yl Acetate (***17***) with PtO_2_

Compound **17** (30 mg, 0.16 mmol, 1 equiv) was dissolved in a stirred 1/1 solution of HCOOH/H_2_O (4 mL), under an atmosphere of N_2_. PtO_2_ (3.6 mg, 0.016 mmol, 0.1 equiv) was added and the reaction mixture was heated at 55 °C for 2 h (TLC analysis showed the absence of the starting material spot and the presence of one new spot). The cooled reaction mixture was filtered through celite, water (10 mL) was added to the filtrate, and the aqueous solution was extracted with EtOAc (3 × 10 mL). The combined organic phases were washed with brine (20 mL), dried over anhydrous Na_2_SO_4_, and evaporated under vacuum. The acquired crude residue was purified by flash column chromatography (20% EtOAc in hexane) to give 2-hydroxy-8-methoxy-1-naphthonitrile (**12**) (29 mg, 45%) as a yellow solid, m.p. = 172–174 °C; R*_f_* = 0.09 (20% EtOAc in hexane) (the ^1^H NMR data were in agreement with those reported for this compound synthesized from compound **11** (Scheme 2)).

### 3.12. Synthesis of 2-Hydroxy-8-methoxy-1-naphthaldehyde (***14***)

To a solution of **17** (250 mg, 1 mmol, 1 equiv) in dry toluene (10 mL) at 0 °C, under an atmosphere of N_2_, was added DIBAL (1.67 mL of 1.2 M in toluene, 2 mmol, 2 equiv), and the reaction mixture was stirred for 0.5 h and then left stirring for 18 h at room temperature. Upon completion of the reaction (TLC examination), the solvent was removed under vacuum, and water (20 mL) was carefully added to the residue. The resulting mixture was cooled to 0 °C, followed by the dropwise addition of 1 M HCl aq. until pH = 1. The aqueous solution was extracted with EtOAc (3 × 10 mL), and the combined organic extracts were washed with brine (20 mL) and dried over anhydrous Na_2_SO_4_; the solvent was removed under vacuum. The acquired crude residue was purified by flash column chromatography (17% EtOAc in hexane) to give the title compound (31.5 mg, 15%) as a yellow solid, m.p. = 67–69 °C (lit. [60], m.p. = 68–70 °C); R*_f_* = 0.54 (20% ethyl acetate in hexane); (the ^1^H NMR data were in agreement with those reported for this compound synthesized from **12** (Scheme 2) and with those previously reported) [60].

### 3.13. Synthesis of 2,8-Dihydroxy-1-naphthonitrile (***5***)

A solution of **2** (500 mg, 2.7 mmol) in dry DMF (15 mL) was heated at 120 °C for 0.5 h (TLC analysis showed the absence of starting material and the presence of one new spot). The reaction was left to cool to room temperature; then, water (100 mL) was added. The reaction mixture was extracted with EtOAc (3 × 20 mL), and the combined organic extracts were washed with brine (20 mL), dried over anhydrous Na_2_SO_4_, and concentrated under vacuum. The acquired crude residue was purified by flash column chromatography (25% EtOAc in hexane) to give the title compound as a yellow solid (400 mg, 80%), m.p. = 166–167 °C (lit. [10], m.p. = 167–168 °C); R*_f_* = 0.1 (20% ethyl acetate in hexane); ^1^H NMR (400 MHz, DMSO-*d*_6_) *δ*: 11.24 (s, 1H), 10.31 (s, 1H), 7.93 (d, *J =* 9.0 Hz, 1H), 7.32 (d, *J =* 7.9 Hz, 1H), 7.25–7.15 (m, 2H), 6.92 (dd, *J =* 7.6, 1.1 Hz, 1H) (in agreement with the ^1^H NMR data that were previously reported for this compound) [10].

### 3.14. Synthesis of 2,8-Dihydroxy-1-naphthaldehyde (***19***)

A closed vessel with a magnetic stir bar was charged with Ni(OAc)_2_·4H_2_O (20 mg, 0.11 mmol, 0.2 equiv), followed by water (1 mL), and the mixture was stirred at room temperature for a few minutes. Ca(H_2_PO_2_)_2_ (90 mg, 0.54 mmol, 1 equiv) was then added, followed by Ca(OAc)_2_·H_2_O (40 mg, 0.22 mmol, 0.4 equiv), compound **5** (100 mg, 0.54 mmol, 1 equiv), and EtOH (1 mL). The reaction mixture was heated at 100 °C for 24 h. Upon completion (TLC analysis), the reaction was left to cool to room temperature, and water (10 mL) was added. The reaction mixture was then extracted with EtOAc (3 × 10 mL), and the combined organic extracts were washed with brine (10 mL), dried over anhydrous Na_2_SO_4_, and concentrated under vacuum. The acquired crude residue was purified by flash column chromatography (17% EtOAc in hexane) to give the title compound (29 mg, 29%) as a yellow solid, m.p. = 194–196 °C (lit. [14], m.p. = 195–197 °C); R*_f_* = 0.51 (33% EtOAc in hexane); ^1^H NMR (400 MHz, CDCl_3_) *δ*: a broad singlet corresponding to OH is not visible, 11.33 (s, 1H), 7.92 (d, *J =* 9.1 Hz, 1H), 7.42 (d, *J =* 7.9 Hz, 1H), 7.23 (d, *J =* 7.7 Hz, 1H), 7.14 (d, *J =* 9.0 Hz, 1H), 6.98 (d, *J =* 7.4 Hz, 1H) (in agreement with the ^1^H NMR data that were previously reported for this compound [14].

### 3.15. Synthesis of 2-Hydroxy-8-methoxy-1-naphthaldehyde (***14***) and 8-Hydroxy-2-methoxy-1-naphthaldehyde (***20***)

To a solution of **19** (25 mg, 0.133 mmol, 1 equiv) in dry DMF (5 mL), under an atmosphere of N_2_, oven-dried K_2_CO_3_ (19 mg, 0.134 mmol, 1.05 equiv) was added, followed by MeI (19 mg, 0.134 mmol, 1.05 equiv), and the reaction was stirred at room temperature for 2 h (TLC showed the absence of the starting material and the presence of two new spots). Water (50 mL) was added; the reaction mixture was extracted with EtOAc (3 × 20 mL); and the combined organic extracts were washed with brine (20 mL), dried over anhydrous Na_2_SO_4_, and concentrated under vacuum. The acquired crude residue was purified by flash column chromatography (11% EtOAc in hexane) to give the title compounds.

Compound **14**: (5.1 mg, 19%) as a yellow solid, m.p. = 67–68 °C (lit. [60], m.p. = 68–70 °C); R*_f_* = 0.54 (20% ethyl acetate in hexane); (the ^1^H NMR data were in agreement with those reported for this compound synthesized from **4** (Scheme 2) and with those previously reported) [60].
Compound **20**: (12.5 mg, 47%) as a yellow solid, m.p. = 110–112 °C); R*_f_* = 0.28 (20% ethyl acetate in hexane); ^1^H NMR (400 MHz, CDCl_3_) *δ*: 12.00 (s, 1H), 10.59 (s, 1H), 8.12 (d, *J =* 9.3 Hz, 1H), 7.35 (d, *J =* 7.7 Hz, 1H), 7.29–7.26 (m, 1H), 7.22 (d, *J =* 9.2 Hz, 1H), 7.14 (dd, *J =* 7.6, 1.4 Hz, 1H), 4.08 (s, 3H) (in agreement with the ^1^H NMR data that were previously reported for this compound) [15].

### 3.16. Synthesis of 2,8-Dimethoxy-1-naphthonitrile (***13***)

To a stirred solution of **5** (300 mg, 1.62 mmol, 1 equiv) in acetone (10 mL) was added MeI (460 mg, 3.28 mmol, 2.02 equiv), followed by Na_2_CO_3_ (175 mg, 1.65 mmol, 1.02 equiv) and H_2_O (2 mL). The reaction mixture was gently heated for 3 h, during which time TLC analysis showed complete conversion of the starting material and the presence of one new spot. The solvents were removed under reduced pressure and the oily residue was triturated with hexane to give the title compound (270 mg, 78%) as a colorless solid (hexane), m.p. = 147–149 °C; R*_f_* = 0.21 (20% ethyl acetate in hexane); (the ^1^H NMR data were in agreement with those reported for this compound synthesized from compound 2 (Scheme 2)).

### 3.17. Synthesis of 2,8-Dimethoxy-1-naphthaldehyde (***21***)

To a stirred solution of **13** (210 mg, 1 mmol, 1 equiv) in dry toluene (10 mL) at 0 °C, under an atmosphere of N_2_, was added DIBAL (1.67 mL of 1.2 M in toluene, 2 mmol, 2 equiv), and the reaction mixture stirred for 0.5 h and then left stirring at room temperature for 18 h. Upon completion of the reaction (TLC analysis), the solvent was removed under reduced pressure, cold water (20 mL) was carefully added to the residue, and the resulting mixture was cooled to 0 °C, followed by the dropwise addition of 1 M HCl aq. until pH = 1. The aqueous solution was extracted with EtOAc (3 × 10 mL), and the combined organic extracts were washed with brine (20 mL), dried over anhydrous Na_2_SO_4_, and concentrated under vacuum. The acquired crude residue was purified by flash column chromatography (17% EtOAc in hexane) to give the title compound as a light brown oil (112 mg, 52%), R*_f_* = 0.37 (20% ethyl acetate in hexane); ^1^H NMR (400 MHz, CDCl_3_) δ: 10.75 (s, 1H), 7.85 (d, *J =* 9.1 Hz, 1H), 7.40 (dd, *J =* 8.2, 1.0 Hz, 1H), 7.34–7.25 (m, 2H), 6.86 (dd, *J =* 7.6, 1.0 Hz, 1H), 3.93 (s, 3H), 3.92 (s, 3H) (in agreement with the ^1^H NMR data that were previously reported for this compound) [16].

### 3.18. Procedure F for the Synthesis of 2-Hydroxy-8-methoxy-1-naphthaldehyde (***14***), 8-Hydroxy-2-methoxy-1-naphthaldehyde (***12***), and 2,8-Dihydroxy-1-naphthaldehyde (***19***)

To an oven-dried closed vessel with a magnetic stirrer bar, under an atmosphere of N_2_, was added **13** (50 mg, 0.231 mmol, 1 equiv), MgBr_2_ diethyl etherate (119 mg, 0.462 mmol, 2 equiv), KI (76.5 mg, 0.642 mmol, 2 equiv), and MeCN (10 mL). The vessel was sealed and then heated with stirring at 150 °C for 2 h. TLC analysis of the cooled reaction mixture showed the absence of the starting material and the presence of three new spots, one intense and two very faint (visualized under a UV lamp). The solvent was removed under vacuum, cooled water (20 mL) was carefully added to the residue, and the resulting cooled mixture was acidified by adding dropwise 1 M HCl aq. to pH = 1. The aqueous mixture was extracted with EtOAc (3 × 10 mL); the combined organic extracts were washed with brine (10 mL), dried over anhydrous Na_2_SO_4_, and concentrated under reduced pressure. The acquired crude residue was purified by flash column chromatography (11% EtOAc in hexane) to give the title compound (4.6 mg, 10%) as a yellow solid, m.p. = 67–69 °C (lit. [60], m.p. = 68–70 °C); R*_f_* = 0.54 (20% EtOAc in hexane); (the ^1^H NMR data were in agreement with those reported for this compound synthesized from compound (**12**) (Scheme 2) and with those previously reported) [60].

### 3.19. Procedure G for the Synthesis of 2-Hydroxy-8-methoxy-1-naphthaldehyde (***14***), 8-Hydroxy-2-methoxy-1-naphthaldehyde (***20***), and 2,8-Dihydroxy-1-naphthaldehyde (***19***)

A solution of **21** (50 mg, 0.231 mmol, 1 equiv) in dry DCM (10 mL), over an atmosphere of N_2_, was cooled to 0 °C; then, BBr_3_ (690 μL of a 1 M solution in DCM, 0.693 mmol, 3 equiv) was added dropwise. The reaction was left stirring at room temperature for 18 h (TLC analysis showed complete conversion of the starting material and the presence of three new spots) and then cooled to 0 °C, quenched slowly with cold water (30 mL), and extracted with DCM (3 × 50 mL). The combined organic extracts were washed with brine (20 mL), dried over anhydrous Na_2_SO_4_, and concentrated under reduced pressure. The acquired crude residue was purified by flash column chromatography (11% EtOAc in hexane) to give the title compounds.

Compound **14**: (3.7 mg, 8%) as a yellow solid, m.p. = 67–68 °C (lit. [60], m.p. = 68–70 °C); R*_f_* = 0.54 (20% EtOAc in hexane); (the ^1^H NMR data were in agreement with those reported for this compound synthesized from (**12**) (Scheme 2) and with those previously reported) [60].
Compound **20**: (3.2 mg, 7%) as a yellow solid, m.p. = 110–112 °C); R*_f_* = 0.28 (20% ethyl acetate in hexane); ^1^H NMR (400 MHz, CDCl_3_) *δ*: 12.00 (s, 1H), 10.59 (s, 1H), 8.12 (d, *J =* 9.3 Hz, 1H), 7.35 (d, *J =* 7.7 Hz, 1H), 7.29–7.26 (m, 1H), 7.22 (d, *J =* 9.2 Hz, 1H), 7.14 (dd, *J =* 7.6, 1.4 Hz, 1H), 4.08 (s, 3H) (in agreement with the ^1^H NMR data that were previously reported for this compound) [15].
Compound **19**: (25.6 mg, 59%) as a yellow solid, m.p. = 194–196 °C (lit. [14], m.p. = 195–197 °C); R*_f_* = 0.51 (33% EtOAc in hexane); (the ^1^H NMR data were in agreement with those reported for this compound synthesized from compound (**5**) (Scheme 2) and with those previously reported) [14].

### 3.20. Procedure H for the Synthesis of 2-Hydroxy-8-methoxy-1-naphthaldehyde (***14***), 8-Hydroxy-2-methoxy-1-naphthaldehyde (***20***), and 2,8-Dihydroxy-1-naphthaldehyde (***19***)

A solution of **21** (50 mg, 0.231 mmol, 1 equiv) in dry DCM (10 mL), over an atmosphere of N_2_, was cooled to 0 °C; then, BBr_3_ (230 μL of a 1M solution in DCM, 0.231 mmol, 1 equiv) was added dropwise, and the reaction was left stirring at room temperature for 1 h. TLC analysis showed complete conversion of the starting material and the presence of three new spots (visualized under a UV lamp). The reaction mixture was cooled to 0 °C, quenched slowly with cold water (30 mL), and extracted with DCM (3 × 20 mL); the combined organic extracts were washed with brine (20 mL), dried over anhydrous Na_2_SO_4_, and concentrated under vacuum. The acquired crude residue was purified by flash column chromatography (11% EtOAc in hexane) to give the title compounds.

Compound **14**: (3.7 mg, 19%) as a yellow solid, m.p. = 67–68 °C (lit. [60], m.p. = 68–70 °C); R*_f_* = 0.54 (20% EtOAc in hexane); (the ^1^H NMR data were in agreement with those reported for this compound synthesized from (**12**) (Scheme 2) and with those previously reported) [60].
Compound **20**: (5 mg, 11%) as a yellow solid, m.p. = 110–112 °C; R*_f_* = 0.28 (20% ethyl acetate in hexane); (the ^1^H NMR data were in agreement with those reported for this compound synthesized from compound (**21**) (Scheme 2) and with those previously reported) [15].
Compound **19**: (14.3 mg, 33%) as a yellow solid, m.p. = 194–196 °C (lit. [14], m.p. = 195–197 °C); R*_f_* = 0.51 (33% EtOAc in hexane); (the ^1^H NMR data were in agreement with those reported for this compound synthesized from compound (**13**) (Scheme 2) and with those previously reported) [14].

### 3.21. Procedure I for the Synthesis of 2-Hydroxy-8-methoxy-1-naphthaldehyde (***14***), 8-Hydroxy-2-methoxy-1-naphthaldehyde (***20***), and 2,8-Dihydroxy-1-naphthaldehyde (***19***)

A solution of **21** (50 mg, 0.231 mmol, 1 equiv) in dry DCM (10 mL), over an atmosphere of N_2_, was cooled to −15 °C; then, BBr_3_ (230 μL of a 1 M solution in DCM, 0.231 mmol, 1 equiv) was added dropwise over a period of 10 min. The reaction was allowed to slowly reach room temperature and was left stirring for 1 h, after which TLC analysis showed complete conversion of the starting material and the presence of three new spots (visualized under a UV lamp). The reaction mixture was cooled to 0 °C, quenched slowly with cold water (30 mL), and extracted with DCM (3 × 20 mL). The combined organic extracts were washed with brine (20 mL), dried over anhydrous Na_2_SO_4_, and concentrated under reduced pressure. The acquired crude residue was purified by flash column chromatography (11% EtOAc in hexane) to give the title compounds.

Compound **14**: (25.9 mg, 56%) as a yellow solid, m.p. = 67–68 °C (lit. [60], m.p. = 68–70 °C); R*_f_* = 0.54 (20% EtOAc in hexane); (the ^1^H NMR data were in agreement with those reported for this compound synthesized from (**12**) (Scheme 2) and with those previously reported) [60].
Compound **20**: (10 mg, 22%) as a yellow solid, m.p. = 110–112 °C); R*_f_* = 0.28 (20% ethyl acetate in hexane); (the ^1^H NMR data were in agreement with those reported for this compound synthesized from compound (**21**) (Scheme 2) and with those previously reported) [15].
Compound **19**: (4.3 mg, 10%) as a yellow solid, m.p. = 194–196 °C (lit. [14], m.p. = 195–197 °C); R*_f_* = 0.51 (33% EtOAc in hexane); (the ^1^H NMR data were in agreement with those reported for this compound synthesized from compound (**5**) (Scheme 2) and with those previously reported) [14].

### 3.22. Procedure J for the Synthesis of 2,8-Bis(benzyloxy)-1-naphthonitrile (***22***) and 2-(Benzyloxy)-8-hydroxy-1-naphthonitrile (***23***)

To a stirred solution of **2** (500 mg, 2.7 mmol, 1 equiv) in dry acetone (20 mL), under an atmosphere of N_2_, was added benzyl bromide (462 mg, 2.7 mmol, 1 equiv), oven-dried K_2_CO_3_ (391 mg, 2.83 mmol, 1.05 equiv), and KI (89 mg, 0.54 mmol, 0.2 equiv). The reaction was left stirring at room temperature for 18 h, after which TLC analysis indicated the absence of the starting material and the presence of two new spots. The solvent was removed under vacuum, the residue was dissolved in EtOAc (30 mL) and washed with water (3 × 10 mL). The organic extract was dried over anhydrous Na_2_SO_4_ and concentrated under reduced pressure. The acquired crude residue was purified by flash column chromatography (20% EtOAc in hexane) to give the title compounds **22** and **23**.

Compound **22**: (118 mg, 12%) as an orange oil; R*_f_* = 0.38 (20% EtOAc in hexane); ^1^H NMR (400 MHz, CDCl_3_) *δ*: 7.97 (d, *J =* 9.1 Hz, 1H), 7.73–7.66 (m, 2H), 7.66–7.57 (m, 2H), 7.53–7.31 (m, 9H), 7.06 (dd, *J =* 7.6 Hz, 1.2 Hz, 1H), 5.47 (s, 4H); ^13^C NMR (100.6 MHz, CDCl_3_) *δ*: 162.1, 153.1, 136.4, 136.1, 134.6, 130.0, 128.8 (2C), 128.6 (2C), 128.2, 128.1, 127.8 (2C), 127.0 (2C), 125.3, 125.3, 121.3, 116.8, 114.1, 109.6, 93.8, 71.3, 71.1.; IR (solid): 3067, 3027, 2922, 2887, 2214, 2094, 1737, 1677, 1591 cm^−1^; HRMS (ESI): *m*/*z* [M + H]^+^ calcd. for C_25_H_20_NO_2_: 366.1489, found: 366.1485.
Compound **23**: (450 mg, 61%) as a colorless solid, m.p. = 136–138 °C; R*_f_* = 0.49 (20% ethyl acetate in hexane); ^1^H NMR (400 MHz, CDCl_3_) *δ*: 7.82–7.75 (m, 2H), 7.57 (d, *J =* 8.8 Hz, 1H), 7.41 (d, *J =* 8.8 Hz, 1H), 7.33–7.12 (m, 6H), 5.44 (s, 2H); ^13^C NMR (100.6 MHz, CDCl_3_) *δ*: 156.0, 139.6, 135.7, 131.3, 129.7, 129.3 (2C), 128.1, 127.0, 126.3 (2C), 124.9, 124.4, 123.9, 120.3, 120.2, 110.8, 47.8; IR (solid): 3052, 2920, 2845, 2216, 2117, 1886, 1752 cm^−1^; HRMS (ESI): *m*/*z* [M + H]^+^ calcd. for C_18_H_14_NO_2_: 276.1019, found: 216.1018.

### 3.23. Synthesis of 2-(Benzyloxy)-8-methoxy-1-naphthonitrile (***24***)

This was prepared according to the experimental procedure for the synthesis of (**13**) from (**5**) (Scheme 2). Used as starting material of compound (**23**) (400 mg, 5 mmol, 1 equiv), MeI (724 mg, 5.1 mmol, 1.02 equiv) Na_2_CO_3_ (540 mg, 5.1 mmol, 1.02 equiv), acetone (15 mL) and H_2_O (3 mL), which gave the title compound (384 mg, 93%) as a yellow solid, m.p. = 100–103 °C; R*_f_* = 0.32 (20% EtOAc in hexane); ^1^H NMR (400 MHz, CDCl_3_) *δ*: 7.80 (d, *J =* 9.2 Hz, 1H), 7.57–7.39 (m, 2H), 7.36–7.13 (m, 6H), 6.86 (dd, *J =* 6.3, 2.5 Hz, 1H), 5.31 (s, 2H), 3.97 (s, 3H); ^13^C NMR (100.6 MHz, CDCl_3_) *δ*: 162.0, 154.3, 136., 134.5, 129.9, 128.8 (2C), 128.2, 127.0 (2C), 125.4, 125.2, 121.0, 116.8, 114.1, 107.7, 93.7, 71.3, 55.8; IR (solid): 2919, 2844, 2212, 2101, 1737, 1595 cm^−1^; HRMS (ESI): *m*/*z* [M + H]^+^ calcd. for C_19_H_16_NO_2_: 290.1176, found: 290.1177.

### 3.24. Synthesis of 2-(Benzyloxy)-8-methoxy-1-naphthaldehyde ***25***

This was prepared according to the experimental procedure for the synthesis of compound **21** from **13** (Scheme 2). Used as starting material of compound **24** (100 mg, 0.34 mmol, 1 equiv), DIBAL (580 μL of 1.2 M in toluene, 0.68 mmol, 2 equiv) and dry toluene (10 mL), which gave the title compound (55.6 mg, 55%) as a yellow oil; R*_f_* = 0.46 (20% EtOAc in hexane); ^1^H NMR (400 MHz, CDCl_3_) *δ*: 10.76 (s, 1H), 7.81 (d, *J =* 9.0 Hz, 1H), 7.46–7.27 (m, 8H), 6.86 (dd, *J =* 7.7, 1.1 Hz, 1H), 5.23 (s, 2H), 3.94 (s, 3H); ^13^C NMR (100.6 MHz, CDCl_3_) δ: 195.0, 155.0, 153.7, 136.9, 131.7, 130.5, 128.7 (2C), 128.1, 127.4 (2C), 124.8, 124.1, 123.4, 121.1, 116.4, 106.62, 72.3, 56.0; IR (solid): 2924, 2843, 2210, 1711, 1596 cm^−1^; HRMS (ESI): *m*/*z* [M + H]^+^ calcd. for C_19_H_17_O_3_: 293.1172, found: 293.1168.

### 3.25. Synthesis of 2-Hydroxy-8-methoxy-1-naphthaldehyde (***14***)

To a stirred solution of **25** (20 mg, 0.099 mmol) in anhydrous MeOH (5 mL), under an atmosphere of N_2_, 5% Pd/C (1 mg, 5%) was added, and the reaction was purged with H_2_. Stirring at room temperature was continued for 18 h (TLC analysis showed complete conversion of the starting material and the presence of a new spot. The reaction mixture was filtered, and the solvent was removed under vacuum. To the remaining residue, EtOAc (20 mL) was added and then washed with brine (10 mL). The organic layer was dried over anhydrous Na_2_SO_4_ and concentrated under reduced pressure. The acquired crude product was purified by flash column chromatography (20% EtOAc in hexane) to give the title compound (18 mg, 92%) as a yellow solid, m.p. = 67–69 °C (lit. [60], m.p. = 68–70 °C); R*_f_* = 0.54 (20% ethyl acetate in hexane); (the ^1^H NMR data were in agreement with those reported for this compound synthesized from compound **12** (Scheme 2) and with those previously reported) [60].

### 3.26. Synthesis of (E)-2-Hydroxy-8-methoxy-1-naphthaldehyde Oxime (***6***)

To a stirred solution of **14** (200 mg, 0.99 mmol, 1 equiv) in MeOH (20 mL), NH_2_OH.HCl (76.4 mg, 0.0011 mmol, 1.1 equiv) was added, and the resulting mixture was cooled to 0 °C, followed by the dropwise addition of an aqueous saturated Na_2_CO_3_ solution until pH = 8. The reaction mixture was left stirring at room temperature for 1 h. TLC analysis showed the absence of the starting material and the presence of a new spot on the baseline. The solution was cooled to 0 °C, followed by the dropwise addition of MeCO_2_H until pH = 5. The solvent was evaporated under vacuum, water (25 mL) was added to the residue, and the mixture was extracted with EtOAc (3 × 10 mL). The combined organic extracts were washed with brine (10 mL), dried over anhydrous Na_2_SO_4_, and concentrated under reduced pressure. The acquired light yellow solid was purified by flash column chromatography (20% EtOAc in hexane) to give the title compound (164 mg, 78%) as a yellow solid, m.p. = 142–143 °C (lit. [60], m.p. = 141–143 °C); R*_f_* = 0.32 (20% EtOAc in hexane); ^1^H NMR (400 MHz, DMSO-*d*_6_) *δ*: 12.06 (s, 1H), 11.45 (s, 1H), 9.65 (s, 1H), 7.83 (d, *J =* 9.0 Hz, 1H), 7.46 (d, *J =* 6.8 Hz, 1H), 7.29 (t, *J =* 7.9 Hz, 1H), 7.18 (d, *J =* 8.9 Hz, 1H), 7.08 (d, *J =* 7.9 Hz, 1H), 3.94 (s, 3H) (in agreement with the ^1^H NMR data that were previously reported for this compound) [60].

### 3.27. Synthesis of 9-Methoxynaphtho[1,2-d]isoxazole 2-Oxide (***8***)

To a stirred solution of (*E*)-oxime **6** (150 mg, 0.70 mmol, 1 equiv) in dry *t*-BuOH (15 mL), under an atmosphere of N_2_, PIDA (450 mg, 1.40 mmol, 2 equiv) was added, and the resulting mixture was stirred at room temperature for 0.5 h. TLC analysis showed the absence of the starting material and the presence of a new spot. Water (15 mL) was added, followed by the dropwise addition of 5% NaHCO_3_ aq. solution until pH = 7–8. The solvents were evaporated under vacuum and water (20 mL) was added to the residue, and the resulting mixture was extracted with EtOAc (3 × 10 mL). The combined organic extracts were washed with brine (10 mL), dried over anhydrous Na_2_SO_4_, and concentrated under reduced pressure. The acquired residue was purified by flash column chromatography (17% EtOAc in hexane) to give the title compound (120 mg, 81%) as a yellow solid, m.p. = 91–93 °C; R*_f_* = 0.44 (20% EtOAc in hexane); ^1^H NMR (400 MHz, CDCl_3_) *δ*: 8.10 (s, 1H), 7.89 (d, *J =* 8.9 Hz, 1H), 7.52 (d, *J =* 8.1 Hz, 1H), 7.47 (t, *J =* 7.9 Hz, 1H), 7.36 (d, *J =* 9.0 Hz, 1H), 7.02 (d, *J =* 7.6 Hz, 1H), 4.07 (s, 3H); ^13^C NMR (100.6 MHz, CDCl_3_) *δ*: 155.2, 149.7, 131.9, 129.33, 126.1, 121.2, 117.4, 112.2, 111.3, 108.4, 106.6, 55.8; IR (solid) 3098, 2919, 2840, 2365, 2209, 2107, 1743, 1568 cm^−1^; HRMS (ESI): *m*/*z* [M + H]^+^ calcd. for C_12_H_10_NO_3_: 216.0661, found: 216.0664.

### 3.28. In Situ Generation of 2-Hydroxy-8-methoxy(naphthalen-1-yl)nitrile Oxide (***9***)

A solution of isoxazole 2-oxide **8** (5 mg, 0.023 mmol) in dry DMSO-*d*_6_ (0.5 mL), under an atmosphere of N_2_, was stirred at room temperature for 6 h. TLC analysis revealed the absence of the starting material and the presence of a new spot. The solution was transferred to an NMR tube and the ^1^H and ^13^C NMR spectra of the new compound were recorded. R*_f_* = 0.2 (20% EtOAc in hexane); ^1^H NMR (400 MHz, DMSO-*d*_6_) *δ*: 11.29 (s, 1H), 7.92 (d, *J =* 9.0 Hz, 1H), 7.46 (dd, *J =* 8.2, 1.0 Hz, 1H), 7.39–7.20 (m, 2H), 7.07 (dd, *J =* 7.8, 1.0 Hz, 1H), 3.98 (s, 3H).; ^13^C NMR (100.6 MHz, DMSO-*d*_6_) *δ*: 161.4, 153.3, 132.8, 128.9, 124.4, 124.4, 121.2, 117.5, 107.6, 88.7, 56.4.; HRMS (ESI): *m*/*z* [M + H]^+^ calcd. for C_12_H_10_NO_3_: 216.0661, found: 216.0661.

### 3.29. Procedure K for the Synthesis of Dimethyl 3-(2-Hydroxy-8-methoxynaphthalen-1-yl)isoxazole-4,5-dicarboxylate (***10a***), 8-Methoxy-1-(5-phenylisoxazol-3-yl)naphthalen-2-ol (***10b***), Methyl 3-(2-Hydroxy-8-methoxynaphthalen-1-yl)-4,5-dihydroisoxazole-5-carboxylate (***10c***) and 8-Methoxy-1-(5-phenyl-4,5-dihydroisoxazol-3-yl)naphthalen-2-ol (***10d***)

A solution of 2-oxide **8** (5 mg, 0.023 mmol, 1 equiv) in dry DMSO (1.5 mL), under an atmosphere of N_2_, was stirred at room temperature for 15 min. DMAD, phenylacetylene, methyl acrylate, or styrene (0.069 mmol, 3 equiv) was added, and the resulting mixture was stirred at room temperature for 18 h. TLC analysis showed the absence of the starting material and the presence of a new spot (visualized under a UV lamp). The reaction was quenched with water (15 mL), and the resulting mixture was extracted with EtOAc (3 × 10 mL). The combined organic extracts were washed with brine (10 mL), dried over anhydrous Na_2_SO_4_, and concentrated under vacuum. The acquired residue was purified by flash column chromatography (17% EtOAc in hexane) to give the title compounds.

Compound **10a**: (7 mg, 85%) as a yellow solid, m.p. = 149–151 °C; R*_f_* = 0.09 (20% EtOAc in hexane); ^1^H NMR (400 MHz, CDCl_3_) *δ*: a singlet corresponding to OH is not visible, 7.83 (d, *J =* 8.9 Hz, 1H), 7.41 (d, *J =* 8.1 Hz, 1H), 7.32–7.21 (m, 1H), 6.79 (d, *J =* 7.7 Hz, 1H), 4.05 (s, 3H), 3.62 (s, 3H), 3.38 (s, 3H); ^13^C NMR (100.6 MHz, CDCl_3_) *δ*: 162.2, 160.5, 159.2, 157.2, 154.4, 153.4, 132.9, 130.4, 124.2, 124.0, 121.5, 118.7, 118.3, 106.6, 103.8, 55.3, 53.6, 52.2; IR (solid) 3352, 2922, 2848, 2364, 2119, 1717, 1613, 1520 cm^−1^; HRMS (ESI): *m*/*z* [M + H]^+^ calcd. for C_18_H_16_NO_7_: 358.0921, found: 358.0916.
Compound **10b**: (6.2 mg, 93%) as a colorless solid, m.p. = 108–110 °C; R*_f_* = 0.49 (20% EtOAc in hexane); ^1^H NMR (400 MHz, CDCl_3_) *δ*: 8.61 (s, 1H), 7.88–7.79 (m, 3H), 7.55–7.41 (m, 4H), 7.37–7.29 (m, 2H), 6.90 (d, *J =* 7.6 Hz, 1H), 6.55 (s, 1H), 3.72 (s, 3H); ^13^C NMR (100.6 MHz, CDCl_3_) *δ*: 168.01, 167.4, 154.9, 154.0, 132.4, 130.9, 130.4, 129.2 (2C), 127.7, 126.0 (2C), 124.1, 123.5, 121.6, 118.8, 107.6, 105.2, 103.9, 55.3; IR (solid) 3205, 2919, 2845, 2363, 2123, 1732, 1606 cm^−1^; HRMS (ESI): *m*/*z* [M + H]^+^ calcd. for C_20_H_16_NO_3_: 318.1125, found: 318.1125.
Compound **10c**: (6.2 mg, 89%) as a yellow solid, m.p. = 118–120 °C; R*_f_* = 0.06 (20% EtOAc in hexane); ^1^H NMR (400 MHz, CDCl_3_) *δ*: a singlet corresponding to OH is not visible, 7.76 (d, *J =* 8.9 Hz, 1H), 7.40 (d, *J =* 8.0 Hz, 1H), 7.29 (t, *J =* 7.9 Hz, 1H), 7.22 (d, *J =* 8.9 Hz, 1H), 6.90 (d, *J =* 7.8 Hz, 1H), 5.20 (t, *J =* 8.6 Hz, 1H), 3.88 (s, 3H), 3.86 (s, 3H), 3.58 (d, *J =* 8.6 Hz, 2H); ^13^C NMR (100.6 MHz, CDCl_3_) *δ*: 171.4, 158.6, 154.4, 153.3, 132.3, 130.6, 124.3, 124.1, 121.7, 118.7, 108.0, 104.7, 56.3, 53.0, 45.2; IR (solid) 3173, 2928, 2848, 2364, 2122, 1899, 1736, 1607, 1516 cm^−1^; HRMS (ESI): *m*/*z* [M + H]^+^ calcd. for C_16_H_16_NO_5_: 302.1023, found: 302.1028.
Compound **10d**: (7.1 mg, 97%) as a colorless solid, m.p. = 205–207 °C; R*_f_* = 0.34 (20% EtOAc in hexane); ^1^H NMR (400 MHz, CDCl_3_) *δ*: 8.31 (s, 1H), 7.67 (d, *J =* 8.9 Hz, 1H), 7.41 (d, *J =* 7.5 Hz, 2H), 7.36 (t, *J =* 7.5 Hz, 2H), 7.30 (t, *J =* 7.8 Hz, 2H), 7.23–7.14 (m, 2H), 6.73 (d, *J =* 7.7 Hz, 1H), 5.73 (dd, *J =* 10.4, 7.4 Hz, 1H), 3.54 (dd, *J =* 16.3, 10.3 Hz, 1H), 3.37–3.29 (m, 4H); ^13^C NMR (100.6 MHz, CDCl_3_) *δ* 159.1, 154.3, 153.4, 141.0, 132.0, 130.6, 128.7 (2C), 128.0, 125.8 (2C), 124.0, 123.9, 121.4, 118.5, 107.3, 105.3, 81.4, 55.2, 49.0; IR (solid) 3135, 2922, 2848, 2363, 2110, 1917, 1754, 1605, 1514 cm^−1^; HRMS (ESI): *m*/*z* [M + H]^+^ calcd. for C_20_H_18_NO_3_: 320.1281, found: 320.1279.

## 4. Conclusions

We developed a fairly efficient four-step synthesis of (*E*)-2-hydroxy-8-methoxy-1-naphthaldehyde from naphtho[1,8-*de*][1,2]oxazin-4-ol with an overall yield of 28.7%. The corresponding aldoxime was oxidized with PIDA to 9-methoxynaphtho[1,2-*d*]isoxazole 2-oxide. From previous works, under similar conditions, (*E*)-2-hydroxy-1-naphthaldehyde oxime afforded naphtho[1,8-*de*][1,2]oxazin-4-ol and not the parent naphtho[1,2-*d*]isoxazole 2-oxide. We therefore attributed the stability of 9-methoxynaphtho[1,2-*d*]isoxazole 2-oxide to the strong H bonding between the 9-OMe substituent and the hydrogen atom of C–1. Clearly, a *peri* substituent like OMe, which is capable of such an intramolecular H bonding interaction, is critical in keeping the heteroring intact, and thus isolable. Indeed, the disruption of this H bond occurs in DMSO by solvation followed by the subsequent ring-opening to its 2-hydroxy-8-methoxy(naphthalen-1-yl)nitrile oxide isomer. So far, this isomerization is a novel transformation which was detected by ^1^H NMR spectroscopy measured by a time-course plot. The formation of a nitrile oxide and not its isocyanate isomer or a furoxan dimer was confirmed by the 1,3-dipolar cycloaddition reactions with various dipolarophiles, which provided access to multi-substituted isoxazoles.

## Figures and Tables

**Table 1:** Methylation of 1-cyano-8-hydroxynaphthalen-2-yl acetate (**16**) ^[a]^.

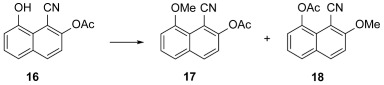							
Entry	Methyl Source (Equiv)	Base (Equiv)	Solvent (Molarity)	T (°C)	Time (h)	Yield (%) ^[a]^	
						Compd. 17	Compd. 18
1	(MeO)_2_SO_4_ (1.1)	K_2_CO_3_ (1.1)	Acetone (0.44)	r.t.	24	10	50
2	MeI (1.1)	K_2_CO_3_ (1.1)	THF (0.44)	r.t.	18	13	47
3	MeI (1.1)	NaH (1.1)	THF (0.44)	r.t.	2	22	40
4	MeI (1.1)	NaH (1.1)	THF (0.04)	r.t.	2	22	40

^[a]^ Isolated yields after purification using flash column chromatography.

**Table 2:** Demethylation of 2,8-dimethoxy-1-naphthaldehyde (**21**) ^[a]^.

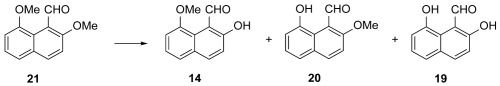							
Entry	Reagents	Solvent (Molarity)	T (°C)	Time (h)	Yield (%) ^[a]^		
					Compd. 14	Compd. 20	Compd. 19
1	MgBr_2_.·Et_2_O (2 equiv) KI (2 equiv)	MeCN (0.02)	150	2	10	0	0
2	BBr_3_ (3 equiv)	DCM (0.02)	0 to r.t.	18	8	7	59
3	BBr_3_ (1 equiv)	DCM (0.02)	0 to r.t.	1	19	11	33
4	BBr_3_ (1 equiv)	DCM (0.02)	–15	1	56	22	10

^[a]^ Isolated yields after purification using flash column chromatography.

## Data Availability

Data are contained within the article and supplementary materials.

## Supplementary Materials

The following supporting information can be downloaded at: https://www.mdpi.com/article/10.3390/molecules29010048/s1, Figures S1, S5, S6, S10, S14, S15, S19, S23, S27, S31–S34, S38, S42, S46, S50, S51, S55, S56, S62, S66, S70 and S74—^1^H NMR spectra of the compounds **11**, **5**, **12**, **13**, **14**, **15**, **16**, **17, 18, 19, 20, 21, 23, 24, 25, 6, 8, 9, 10a, 10b, 10c, 10d**; Figures S2, S7, S11, S16, S20, S24, S28, S35, S39, S43, S47, S52, S57, S63, S67, S71, and S75—^13^C NMR spectra of the compounds **11**, **12**, **13**, **15**, **16**, **17**, **18**, **22**, **23**, **24**, **25**, **10a**, **10b**, **10c**, **10d**; Figures S3, S8, S12, S17, S21, S25, S29, S36, S40, S44, S48, S53, S58, S64, S68, S72 and S76—HRMS spectra of the compounds **11**, **12**, **13**, **15**, **16**, **17**, **18**, **22**, **23**, **24**, **25**, **8**, **9**, **10a**, **10b**, **10c**, **10d**; Figures S4, S9, S13, S18, S22, S26, S30, S37, S41, S45, S49, S54, S65, S69, S73 and S77—IR spectra of the compounds **11**, **12**, **13**, **15**, **16**, **17**, **18**, **22**, **23**, **24**, **25**, **8**, **10a**, **10b**, **10c**, **10d**; Figure S59—^1^H NMR spectra of the compounds **9** and **8** superimposed; Figure S60—^1^H NMR spectra of the compounds **9** and **8** stacked; Figure S61—^1^H NMR spectra of the time-course experiment of the ring-opening of compound **8** to compound **9**.

## References

[1] Majireck M.M., Bennett J.M. (2021). 1,2-Oxazines and Their Benzo Derivatives. Comprehensive Heterocyclic Chemistry IV.

[2] Chada R.R., Kajare R.C., Bhandari M.C., Mohammed S.Z., Khatravath M., Warudikar K., Punna N. (2021). Facile Access to [1,2]-Oxazine Derivatives via annulations of Aminoxy-Tethered 1,7-Enynes. Org. Biomol. Chem..

[3] Somu C., Mohan C.D., Ambekar S., Dukanya, Rangappa S., Baburajeev C.P., Sukhorukov A., Mishra S., Shanmugam M.K., Chinnathambi A. (2020). Identification of a Novel 1,2 Oxazine That Can Induce Apoptosis by Targeting NF-ΚB in Hepatocellular Carcinoma Cells. Biotechnol. Rep..

[4] Gaonkar S.L., Nagaraj V.U., Nayak S. (2018). A Review on Current Synthetic Strategies of Oxazines. Mini Rev. Org. Chem..

[5] Tsoungas P.G. (2002). 1,2-Oxazines and Their *N*-Oxides in Synthesis. Heterocycles.

[6] Tsoungas P.G. (2002). Synthesis of 1,2-Oxazines and Their *N*-Oxides. Heterocycles.

[7] Adams R., Geissman T.A. (1938). Structure of Gossypol. VIII. ^1^ Derivatives of the Ethers of Gossypol. J. Am. Chem. Soc..

[8] Adams R., Burney D.E. (1941). The Reactions of 2,8-Dihydroxynaphthaldehyde. J. Am. Chem. Soc..

[9] Royer R.E., Deck L.M., Campos N.M., Hunsaker L.A., Vander Jagt D.L. (1986). Biologically Active Derivatives of Gossypol: Synthesis and Antimalarial Activities of Peri-Acylated Gossylic Nitriles. J. Med. Chem..

[10] Supsana P., Tsoungas P.G., Varvounis G. (2000). A Novel One-Pot Synthesis of Isomeric Naphtho[1,2-*d*]Isoxazole 2-Oxide and Naphtho[1,8-de][1,2]Oxazine Ring Systems. A Case of Simultaneous *o*- and *Peri*-Cyclisation in Naphthalene. Tetrahedron Lett..

[11] Supsana P., Tsoungas P.G., Aubry A., Skoulika S., Varvounis G. (2001). Oxidation of 1-Acyl-2-Naphthol Oximes: *Peri*- and *o*-Cyclisation and Spiro Cyclodimerisation of Naphthoquinone Nitrosomethide Intermediates. Tetrahedron.

[12] Dolka C., Van Hecke K., Van Meervelt L., Tsoungas P.G., Van Der Eycken E.V., Varvounis G. (2009). Novel Thermal and Microwave-Assisted Facile Route to Naphthalen-2(1 h)-Ones via an Oxidative Alkoxylation-Ringopening Protocol. Org. Lett..

[13] Bin L., Xue W., Shuang J., Tianyong Z., Jingyi Y., Jingchao W., Xiao S., Xiaoyuan M. (2019). Progress in Synthesis and Application of Naphthalenediol Intermediates with High Value-Added. Chem. Ind. Eng. Prog..

[14] Hügle M., Lucas X., Ostrovskyi D., Regenass P., Gerhardt S., Einsle O., Hau M., Jung M., Breit B., Günther S. (2017). Beyond the BET Family: Targeting CBP/P300 with 4-Acyl Pyrroles. Angew. Chem..

[15] Jiang J., Yuan D., Ma C., Song W., Lin Y., Hu L., Zhang Y. (2021). Palladium-Catalyzed Regiospecific Peri- And Ortho-C-H Oxygenations of Polyaromatic Rings Mediated by Tunable Directing Groups. Org. Lett..

[16] Li F., Zhou Y., Yang H., Wang Z., Yu Q., Zhang F.L. (2019). Monodentate Transient Directing Group Enabled Pd-Catalyzed Ortho-C-H Methoxylation and Chlorination of Benzaldehydes. Org. Lett..

[17] Cordero F.M., Giomi D., Machetti F. (2022). Isoxazoles. Comprehensive Heterocyclic Chemistry IV.

[18] Duc D.X., Dung V.C. (2021). Recent Progress in the Synthesis of Isoxazoles. Curr. Org. Chem..

[19] Das S., Chanda K. (2021). An Overview of Metal-Free Synthetic Routes to Isoxazoles: The Privileged Scaffold. RSC Adv..

[20] Zhu J., Mo J., Lin H.Z., Chen Y., Sun H.P. (2018). The Recent Progress of Isoxazole in Medicinal Chemistry. Bioorg Med. Chem..

[21] Pairas G.N., Perperopoulou F., Tsoungas P.G., Varvounis G. (2017). The Isoxazole Ring and Its *N*-Oxide: A Privileged Core Structure in Neuropsychiatric Therapeutics. ChemMedChem.

[22] Kumar J., Chawla G., Akhtar M., Sahu K., Rathore V., Sahu S. (2017). Design, Synthesis and Pharmacological Evaluation of Some Novel Derivatives of 1-{[3-(Furan-2-yl)-5-Phenyl-4,5-Dihydro-1,2-Oxazol-4-yl]Methyl}-4-Methyl Piperazine. Arab. J. Chem..

[23] Singhal A., Parumala S.K.R., Sharma A., Peddinti R.K. (2016). Hypervalent Iodine Mediated Synthesis of Di- and Tri-Substituted Isoxazoles via [3+2] Cycloaddition of Nitrile Oxides. Tetrahedron Lett..

[24] Imai T., Togo H. (2018). One-Pot Preparation of 3-Arylisoxazole-4,5-dicarboxylates from Benzylic Chlorides and Acetylenedicarboxylates. Eur. J. Org. Chem..

[25] Kobayashi E., Togo H. (2018). Facile One-Pot Preparation of 5-Aryltetrazoles and 3-Arylisoxazoles from Aryl Bromides. Tetrahedron.

[26] Kobayashi E., Togo H. (2019). Facile One-Pot Transformation of Primary Alcohols into 3-Aryl- and 3-Alkyl-Isoxazoles and -Pyrazoles. Synthesis.

[27] Nakano R., Togo H. (2020). Efficient Transformation of Electron-Rich Arenes into Diethyl 3-Arylisoxazole-4,5-Dicarboxylates. Tetrahedron.

[28] Kamath P., Jadhav V., Lal M. (2021). Heterocycle–Heterocycle Strategy for 4,5-Disubstituted Pyrrolidine 2,3-Diones: Reductive Rearrangement Approach from Isoxazole Esters. Synlett.

[29] Azarifar D., Maleki B., Mohammadi K. (2007). Oxidative Aromatization of 3,5-Disubstituted Isoxazolines to the Corresponding Isoxazoles with *N*,*N*,*N*’,*N*’-Tetrabromobenzene-1,3-Disolfonamide (TBBDS) and 1,3-Dibromo-5,5-Dimetylhydantoin (DBH) as Efficient Reagents under Mild Reaction Conditions. Heterocycles.

[30] Azarifar D., Khosravi K., Veisi R.A. (2010). An Efficient Oxidation of 2-Pyrazolines and Isoxazolines by Bis-Bromine-1,4-Diazabicyclo[2.2.2]Octane Complex (DABCO-Br_2_). Arkivoc.

[31] Koyama Y., Matsumura T., Yui T., Ishitani O., Takata T. (2013). Fluorescence Control of Boron Enaminoketonate Using a Rotaxane Shuttle. Org. Lett..

[32] Abdukader A., Sun Y., Zhang Z., Liu C. (2018). TBAI-Catalyzed Intramolecular Annulation of Chalcone Oximes toward Isoxazoles. Catal. Commun..

[33] Carloni L.E., Mohnani S., Bonifazi D. (2019). Synthesis of 3,5-Disubstituted Isoxazoles through a 1,3-Dipolar Cycloaddition Reaction between Alkynes and Nitrile Oxides Generated from O-Silylated Hydroxamic Acids. Eur. J. Org. Chem..

[34] Pramanik N., Sarkar S., Roy D., Debnath S., Ghosh S., Khamarui S., Maiti D.K. (2015). Synthesis and Diverse General Oxidative Cyclization Catalysis of High-Valent Mo^VI^O_2_ (HL) to Ubiquitous Heterocycles and Their Chiral Analogues with High Selectivity. RSC Adv..

[35] Ma L., Jin F., Cheng X., Tao S., Jiang G., Li X., Yang J., Bao X., Wan X. (2021). [2 + 2 + 1] Cycloaddition of *N*-Tosylhydrazones,*tert*-Butyl Nitrite and Alkenes: A General and Practical Access to Isoxazolines. Chem. Sci..

[36] Athappan P.R., Shanti P., Natarajan C. (1995). Synthesis and Spectral Studies of Cobalt(II), Nickel(II) and Copper(II) Complexes of Some Bidentate Isoxazolines. Indian. J. Chem..

[37] Sharma S., Pathak L.V.N., Sharma A. (2010). Synthesis of New 3-(4-Fluoronaphthyl)-5-(Aryl)-2-Isoxazolines and Their Antimicrobial and Spermicidal. Indian. J. Heterocycl. Chem..

[38] Huang H., Li F., Xu Z., Cai J., Ji X., Deng G.J. (2017). Base-Promoted [3+2]-Annulation of Oxime Esters and Aldehydes for Rapid Isoxazoline Formation. Adv. Synth. Catal..

[39] Wang C.Y., Wang C.Y., Teng F., Li Y., Li J.H. (2020). Heteroannulation of Alkenes with Enynyl Benziodoxolones and Silver Nitrite Involving CC Bond Oxidative Cleavage: Entry to 3-Aryl-Δ^2^-Isoxazolines. Org. Lett..

[40] Kociolek M.G., Casbohm J.S. (2013). Benzisoxazole 2-Oxides as Novel UV Absorbers and Photooxidation Inhibitors. J. Phys. Org. Chem..

[41] Boulton A.J., Tsoungas P.G. (1980). 1,2-Benzisoxazole N-Oxides. J. Chem. Soc. Chem. Commun..

[42] Boulton A.J. (1981). Indazole, Indoxazene, and Furazan Oxides: Their Synthesis and Rearrangement. Bull. Des. Sociétés Chim. Belg..

[43] Boulton A.J., Tsoungas P.G., Tsiamis C. (1986). Preparation of 1,2-Benzisoxazole 2-Oxides. J Chem Soc Perkin 1.

[44] Jadhav V.K., Deshmukh A.P., Wadagaonkar P.P., Salunkhe M.M. (2000). Sodium Perborate: A Facile Synthesis of 1,2-Benzisoxazole 2-Oxides. Synth. Commun..

[45] Gardikis Y., Tsoungas P.G., Potamitis C., Zervou M., Cordopatis P. (2011). Xanthones in Heterocyclic Synthesis. An Efficient Route for the Synthesis of C-3 o-Hydroxyaryl Substituted 1,2-Benzisoxazoles and Their *N*-Oxides, Potential Scaffolds for Angiotensin(II) Antagonist Hybrid Peptides. Heterocycles.

[46] Kociolek M.G., Hoermann O. (2012). Synthesis of 1,2-Benzisoxazole 2-Oxides. Synth. Commun..

[47] Raihan M.J., Kavala V., Habib P.M., Guan Q.Z., Kuo C.W., Yao C.F. (2011). Synthesis of Isoxazoline *N*-Oxides via [Hydroxy(Tosyloxy)Iodo]Benzene (HTIB)-Mediated Oxidative N-O Coupling. J. Org. Chem..

[48] Feuer H. (2007). Nitrile Oxides, Nitrones, and Nitronates in Organic Synthesis.

[49] Padwa A., Pearson W.H. (2002). Synthetic Applications of 1,3-Dipolar Cycloaddition Chemistry Toward Heterocycles and Natural Products.

[50] Fershtat L.L., Teslenko F.E. (2021). Five-Membered Hetarene N-Oxides: Recent Advances in Synthesis and Reactivity. Synthesis.

[51] De Angelis L., Crawford A.M., Su Y.-L., Wherritt D., Arman H., Doyle M.P. (2021). Catalyst-Free Formation of Nitrile Oxides and Their Further Transformations to Diverse Heterocycles. Org. Lett..

[52] Roscales S., Plumet J. (2018). Metal-Catalyzed 1,3-Dipolar Cycloaddition Reactions of Nitrile Oxides. Org. Biomol. Chem..

[53] Takikawa H., Sato S., Seki R., Suzuki K. (2017). Oxidative Ring Opening of Benzocyclobutenone Oximes: Novel Access to Stable Nitrile Oxides. Chem. Lett..

[54] Molteni G., Del Buttero P. (2011). Stable Nitrile Oxide Dipolar Cycloadditions in Pure Water. Tetrahedron.

[55] Faita G., Mella M., Mortoni A., Paio A., Quadrelli P., Seneci P. (2002). Solid-Supported Nitrile Oxides as Stable and Valuable Reactive Intermediates. Eur. J. Org. Chem..

[56] Groundwater P.W., Nyerges M., Fejes I., Hibbs D.E., Bendell D., Anderson R.J., McKillop A., Sharif T., Zhang W. (2000). Preparation and Reactivity of Some Stable Nitrile Oxides and Nitrones. Arkivoc.

[57] Grundmann C., Richter R. (1968). Nitrile Oxides. X. Improved Method for the Preparation of Nitrile Oxides from Aldoximes. J. Org. Chem..

[58] Koyama Y., Miura K., Cheawchan S., Seo A., Takata T. (2012). Cascade Functionalization of Unsaturated Bond-Containing Polymers Using Ambident Agents Possessing Both Nitrile *N*-Oxide and Electrophilic Functions. Chem. Commun..

[59] Cheawchan S., Koyama Y., Uchida S., Takata T. (2013). Catalyst-Free Click Cascade Functionalization of Unsaturated-Bond- Containing Polymers Using Masked-Ketene-Tethering Nitrile *N*-Oxide. Polymer.

[60] Tzeli D., Gerontitis I.E., Petsalakis I.D., Tsoungas P.G., Varvounis G. (2022). Self Cycloaddition of o-Naphthoquinone Nitrosomethide to (±) *Spiro* {naphthalene(Naphthopyranofurazan)}-one Oxide: An Insight into Its Formation. ChemPlusChem.

[61] Di Iorio N., Filippini G., Mazzanti A., Righi P., Bencivenni G. (2017). Controlling the C(Sp^3^)–C(Sp^2^) Axial Conformation in the Enantioselective Friedel–Crafts-Type Alkylation of β-Naphthols with Inden-1-ones. Org. Lett..

[62] Hwan M.K., Byeong H.J., Hyon J.Y., Myoung J.A., Mun S.S., Jin H.H., Lee K.J., Chul H.K., Joo T., Hong S.C. (2008). Two-Photon Fluorescent Turn-on Probe for Lipid Rafts in Live Cell and Tissue. J. Am. Chem. Soc..

[63] Saadati F., Meftah-Booshehri H. (2013). Antimony(V) Chloride as an Efficient Reagent for Deprotection of Methyl Ethers. Synlett.

[64] Xi F., Kamal F., Schenerman M.A. (2002). A Novel and Convenient Transformation of Nitriles to Aldehydes. Tetrahedron Lett..

[65] Mouselmani R., Hachem A., Alaaeddine A., Métay E., Lemaire M. (2018). Reduction of Aromatic Nitriles into Aldehydes Using Calcium Hypophosphite and a Nickel Precursor. Org. Biomol. Chem..

[66] Balmond E.I., Tautges B.K., Faulkner A.L., Or V.W., Hodur B.M., Shaw J.T., Louie A.Y. (2016). Comparative Evaluation of Substituent Effect on the Photochromic Properties of Spiropyrans and Spirooxazines. J. Org. Chem..

[67] Waibel M., De Angelis M., Stossi F., Kieser K.J., Carlson K.E., Katzenellenbogen B.S., Katzenellenbogen J.A. (2009). Bibenzyl- and Stilbene-Core Compounds with Non-Polar Linker Atom Substituents as Selective Ligands for Estrogen Receptor Beta. Eur. J. Med. Chem..

[68] Ye Y., Zhang B., Mao R., Zhang C., Wang Y., Xing J., Liu Y.C., Luo X., Ding H., Yang Y. (2017). Discovery and Optimization of Selective Inhibitors of Protein Arginine Methyltransferase 5 by Docking-Based Virtual Screening. Org. Biomol. Chem..

[69] Liu L., Shao L., Li J., Cui H., Li B., Zhou X., Lv P., Zhang J. (2019). Synthesis, Antibacterial Activities, Mode of Action and Acute Toxicity Studies of New Oxazolidinone-Fluoroquinolone Hybrids. Molecules.

[70] Finet J.-P. (1998). Chapter 5 Ligand Coupling Involving Organoiodine Compounds. Ligand Coupling Reactions with Heteroatomic Compounds.

[71] Koyama Y., Lee Y.G., Kuroki S., Takata T. (2015). Synthesis, 13C NMR, and UV Spectroscopic Study of 13C-Labeled Nitrile N-Oxide. Tetrahedron Lett..

