# Supplementary material for: Naphtho[1,8-de][1,2]Oxazin-4-ol: Precursor to 1,2,8-Trisubstituted Naphthalenes and 1-Unsubstituted Naphtho[1,2-d]isoxazole 2-Oxide: A Novel Isomerization of the N-Oxide to Nitrile Oxide en Route to Isoxazol(in)es
Source: Molecules. 2023 Dec 20;29(1):48. doi: 10.3390/molecules29010048 (PMC10779812; doi:10.3390/molecules29010048)
Supplement: Supplementary file 1 [file molecules-29-00048-s001.zip › molecules-2734307-supplementary.pdf]

Supporting Information

Naphtho[1,8-*de*][1,2]Oxazin-4-ol: Precursor to 1,2,8-Trisubstituted Naphthalenes and 1-Unsubstituted Naphtho[1,2-*d*]isoxazole 2-Oxide: A Novel Isomerization of the *N*-Oxide to Nitrile Oxide *en Route* to Isoxazol(in)es

Ioannis E. Gerontitis [1], Petros G. Tsoungas [2] and George Varvounis [1,*]

[1] Section of Organic Chemistry and Biochemistry, Department of Chemistry, University of Ioannina, 451 10 Ioannina, Greece; giannhs_ger@yahoo.gr

[2] Department of Biochemistry, Hellenic Pasteur Institute, 127 Vas. Sofias Ave., 115 21 Athens, Greece; pgt@otenet.gr

* Correspondence: gvarvoun@uoi.gr; Tel.: +30-694-489-9172

Contents

Figure S1. ^{1}H NMR (400 MHz, $CDCl_3$) of compound (**11**). 4

Figure S2. ^{13}C NMR (100.6 MHz, $CDCl_3$) of compound (**11**). 4

Figure S3. HRMS $[M+H]^+$ of compound (**11**) using 0.1% HCO_2H in MeOH as solvent. 4

Figure S4. IR spectrum (solid) of compound (**11**). 5

Figure S5. ^{1}H NMR (400 MHz, DMSO-d_6) of compound (**5**). 5

Figure S6. ^{1}H NMR (400 MHz, $CDCl_3$) of compound (**12**). 6

Figure S7. ^{13}C NMR (100.6 MHz, $CDCl_3$) of compound (**12**). 6

Figure S8. HRMS $[M+H]^+$ of compound (**12**) using 0.1% HCO_2H in MeOH as solvent. 7

Figure S9. IR spectrum (solid) of compound (**12**). 7

Figure S10. ^{1}H NMR (400 MHz, $CDCl_3$) of compound (**13**). 8

Figure S11. ^{13}C NMR (400 MHz, $CDCl_3$) of compound (**13**). 8

Figure S12. HRMS $[M+H]^+$ of compound (**13**) using 0.1% HCO_2H in MeOH as solvent. 9

Figure S13. IR spectrum (solid) of compound (**13**). 9

Figure S14. ^{1}H NMR (400 MHz, $CDCl_3$) of compound (**14**). 10

Figure S15. ^{1}H NMR (400 MHz, DMSO-d_6) of compound (**15**). 10

Figure S16. ^{13}C NMR (100.6 MHz, DMSO-d_6) of compound (**15**). 11

Figure S17. HRMS $[M+H]^+$ of compound (**15**) using 0.1% HCO_2H in MeOH as solvent. 11

Figure S18. IR spectrum (solid) of compound (**15**). 12

Figure S19. ^{1}H NMR (400 MHz, DMSO-d_6) of compound (**16**). 12

Figure S20. ^{13}C NMR (100.6 MHz, DMSO-d_6) of compound (**16**). 13

Figure S21. HRMS $[M+H]^+$ of compound (**16**) using 0.1% HCO_2H in MeOH as solvent. 13

Figure S22. IR spectrum (solid) of compound (**16**). 14

Figure S23. ^{1}H NMR (400 MHz, DMSO-d_6) of (**17**)....................14
Figure S24. ^{13}C NMR (100.6 MHz, DMSO-d_6) of (**17**)....................15
Figure S25. HRMS [M+H]$^+$ of compound (**17**) using 0.1% HCO_2H in MeOH as solvent.............15
Figure S26. IR spectrum (solid) of (**17**)....................16
Figure S27. ^{1}H NMR (400 MHz, DMSO-d_6) of (**18**)....................16
Figure S28. ^{13}C NMR (100.6 MHz, DMSO-d_6) of (**18**)....................17
Figure S29. HRMS [M+H]$^+$ of compound (**18**) using 0.1% HCO_2H in MeOH as solvent.............17
Figure S30. IR spectrum (solid) of (**18**)....................18
Figure S31. ^{1}H NMR (400 MHz, $CDCl_3$) of (**19**)....................18
Figure S32. ^{1}H NMR (400 MHz, $CDCl_3$) of (**20**)....................19
Figure S33. ^{1}H NMR (400 MHz, $CDCl_3$) of (**21**)....................19
Figure S34. ^{1}H NMR (400 MHz, $CDCl_3$) of (**22**)....................20
Figure S35. ^{13}C NMR (100.6 MHz, $CDCl_3$) of (**22**)....................20
Figure S36. HRMS [M+H]$^+$ of compound (**22**) using 0.1% HCO_2H in MeOH as solvent.............21
Figure S37. IR spectrum (solid) of (**22**)....................21
Figure S38. ^{1}H NMR (400 MHz, $CDCl_3$) of (**23**)....................22
Figure S39. ^{13}C NMR (100.6 MHz, $CDCl_3$) of (**23**)....................22
Figure S40. HRMS [M+H]$^+$ of compound (**23**) using 0.1% HCO_2H in MeOH as solvent.............23
Figure S41. IR spectrum (solid) of (**23**)....................23
Figure S42. ^{1}H NMR (400 MHz, $CDCl_3$) of (**24**)....................24
Figure S43. ^{13}C NMR (100.6 MHz, $CDCl_3$) of (**24**)....................24
Figure S44. HRMS [M+H]$^+$ of compound (**24**) using 0.1% HCO_2H in MeOH as solvent.............25
Figure S45. IR spectrum (solid state) of (**24**)....................25
Figure S46. ^{1}H NMR (400 MHz, $CDCl_3$) of (**25**)....................26
Figure S47. ^{13}C NMR [100.6 MHz, $CDCl_3$] of (**25**)....................26
Figure S48. HRMS [M+H]$^+$ of compound (**25**) using 0.1% HCO_2H in MeOH as solvent.............27
Figure S49. IR spectrum (solid state) of (**25**)....................27
Figure S50. ^{1}H NMR (400 MHz, DMSO-d_6) of (**6**)....................28
Figure S51. ^{1}H NMR (400 MHz, $CDCl_3$) of (**8**)....................28
Figure S52. ^{13}C NMR [100.6 MHz, $CDCl_3$] of (**8**)....................29
Figure S53. HRMS [M+H]$^+$ of compound (**8**) using 0.1% HCO_2H in MeOH as solvent...............29
Figure S54. IR spectrum of (**8**)....................30
Figure S55. ^{1}H NMR (400 MHz, DMSO-d_6) of (**9**)....................30
Figure S56. ^{1}H NMR (400 MHz, DMSO-d_6 + D_2O) of (**9**). 31
Figure S57. ^{13}C NMR [100.6 MHz, DMSO-d_6] of (**9**)....................31
Figure S58. HRMS [M+H]$^+$ of compound (**9**) in DMSO-d_6 using 0.1% HCO_2H in MeOH as solvent. 32
Figure S59. ^{1}H NMR (400 MHz, DMSO-d_6) of nitrile oxide (**9**) and isoxazole-2-oxide (**8**), superimposed. 33

Figure S60. ^{1}H NMR (400 MHz, DMSO-d_6) of nitrile oxide (**9**) and isoxazole-2-oxide (**8**), stacked. 33

Figure S61. ^{1}H NMR (400 MHz, DMSO-d_6) time-course experiment of the ring opening of isoxazole-2-oxide (**8**) (top spectra) to nitrile oxide (**9**) (bottom spectra)....33

Figure S62. ^{1}H NMR (400 MHz, $CDCl_3$) of (**10a**)....34

Figure S63. ^{13}C NMR (100.6 MHz, $CDCl_3$) of (**10a**)....34

Figure S64. HRMS $[M+H]^+$ of compound (**10a**) using 0.1% HCO_2H in MeOH as solvent....35

Figure S65. IR spectrum (solid) of (**10a**)....35

Figure S66. ^{1}H NMR (400 MHz, $CDCl_3$) of (**10b**). 36

Figure S67. ^{13}C NMR (100.6 MHz, $CDCl_3$) of (**10b**)....36

Figure S68. HRMS $[M+H]^+$ of compound (**10b**) using 0.1% HCO_2H in MeOH as solvent....37

Figure S69. IR spectrum (solid) of (**10b**). 37

Figure S70. ^{1}H NMR (400 MHz, $CDCl_3$) of (**10c**)....38

Figure S71. ^{13}C NMR (100.6 MHz, $CDCl_3$) of (**10c**). 38

Figure S72. HRMS $[M+H]^+$ of compound (**10c**) using 0.1% HCO_2H in MeOH as solvent. 39

Figure S73. IR spectrum (solid) of (**10c**)....39

Figure S74. ^{1}H NMR (400 MHz, $CDCl_3$) of (**10d**). 40

Figure S75. ^{13}C NMR (100.6 MHz, $CDCl_3$) of (**10d**)....40

Figure S76. HRMS $[M+H]^+$ of compound (**10d**) using 0.1% HCO_2H in MeOH as solvent....41

Figure S77. IR spectrum (solid) of (**10d**). 41

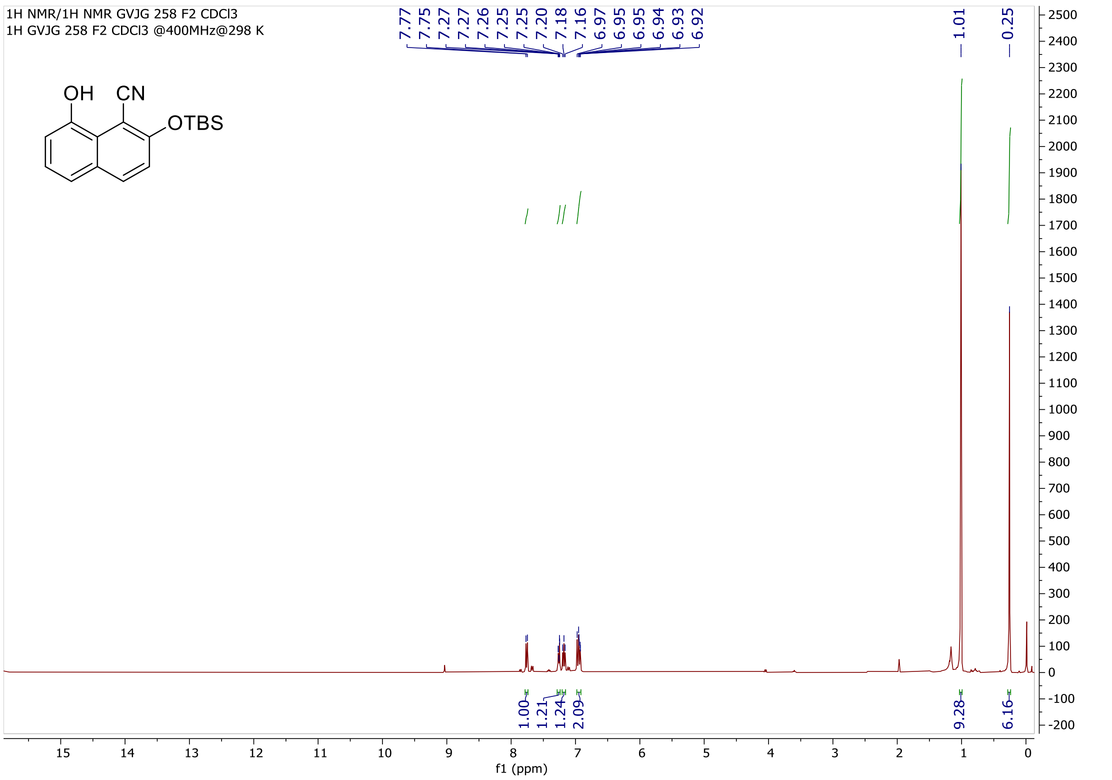

Figure S1. ^{1}H NMR (400 MHz, $CDCl_3$) of compound (**11**).

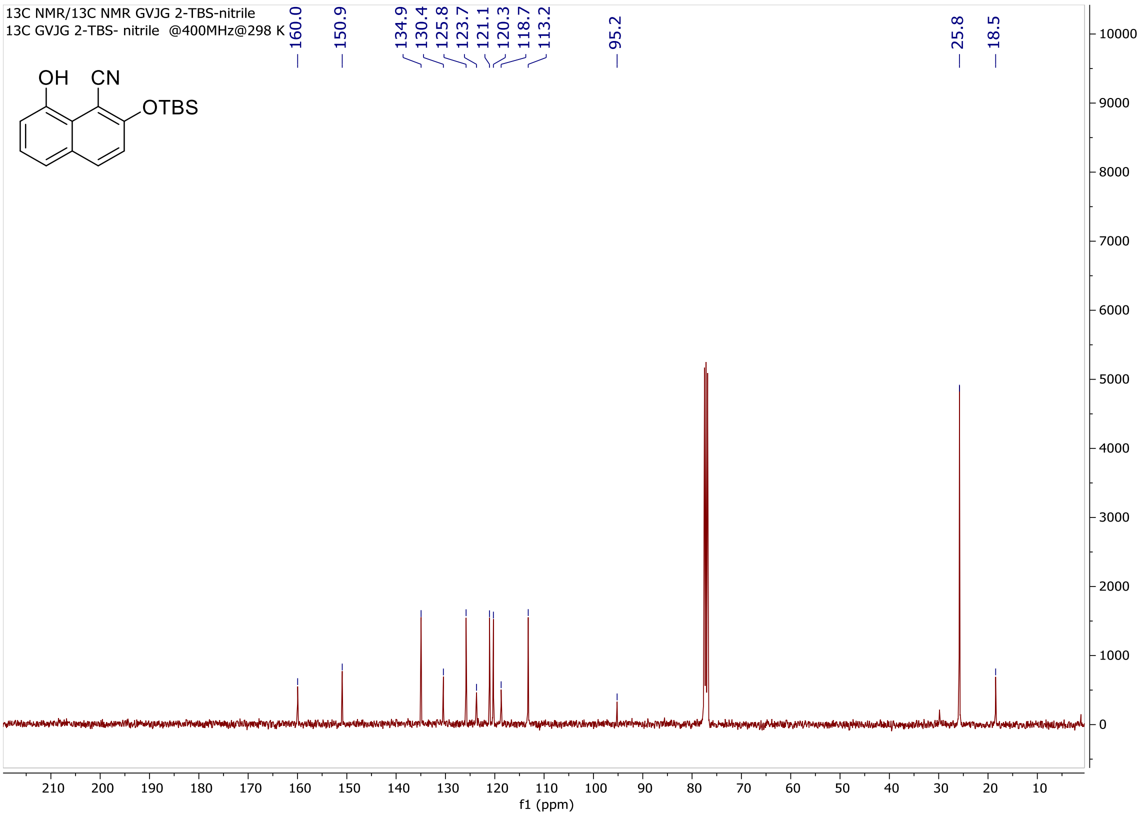

Figure S2. ^{13}C NMR (100.6 MHz, $CDCl_3$) of compound (**11**).

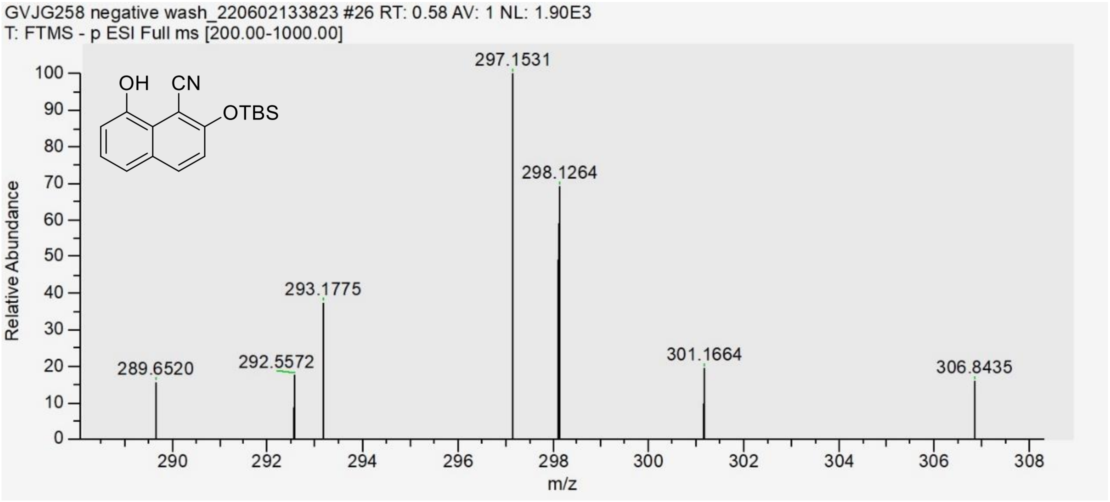

Figure S3. HRMS $[M+H]^{+}$ of compound (**11**) using 0.1% HCO_2H in MeOH as solvent.

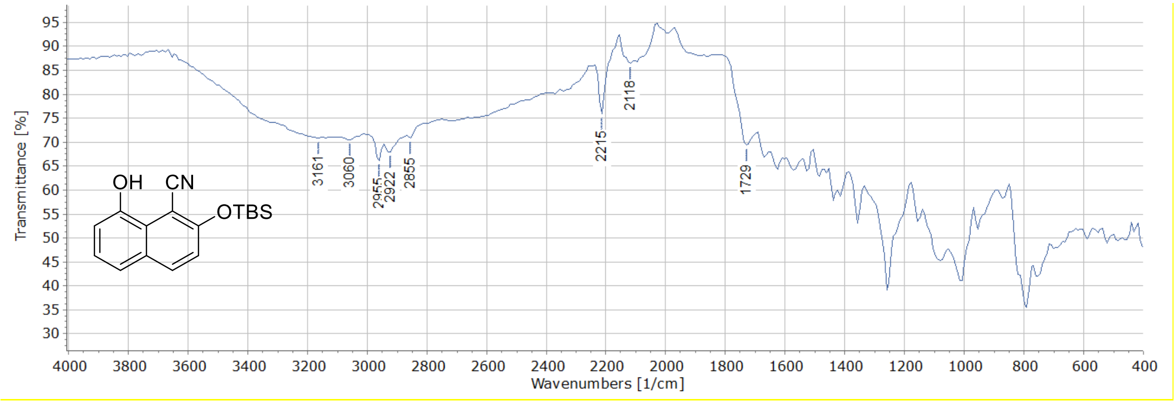

Figure S4. IR spectrum (solid) of compound (**11**).

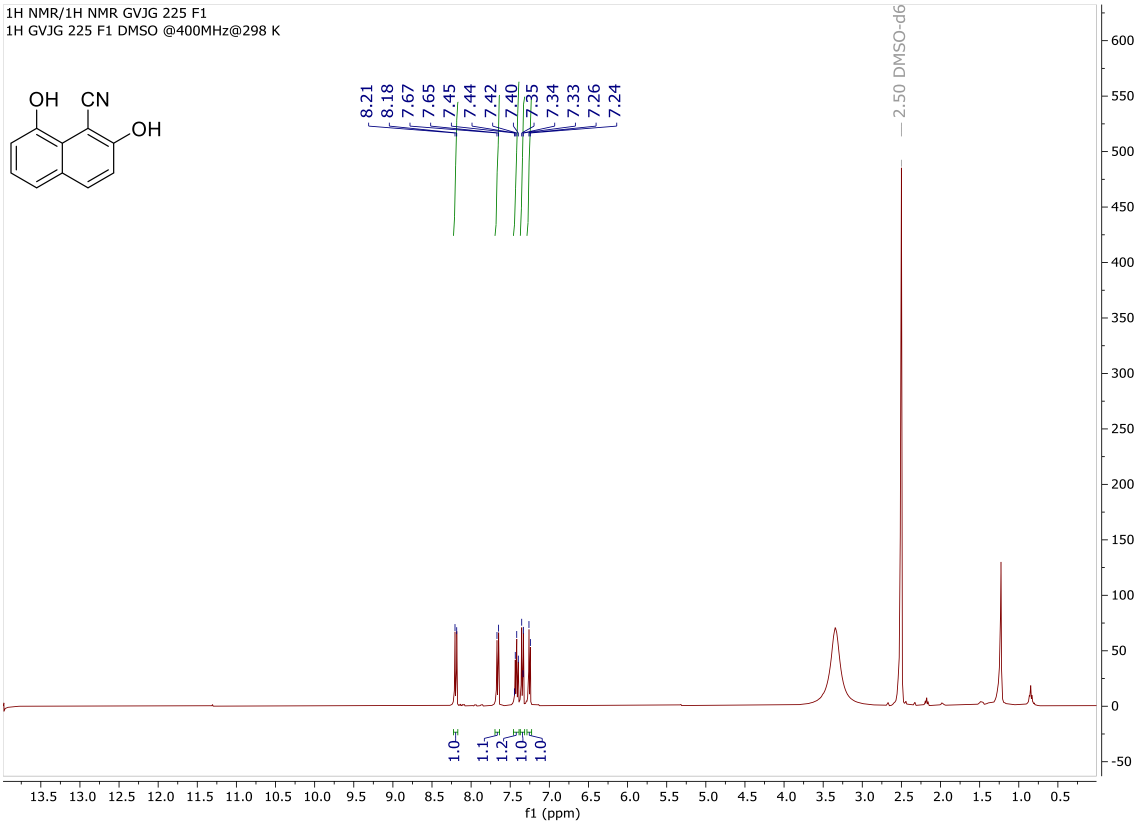

Figure S5. ^{1}H NMR (400 MHz, DMSO-d_6) of compound (**5**).

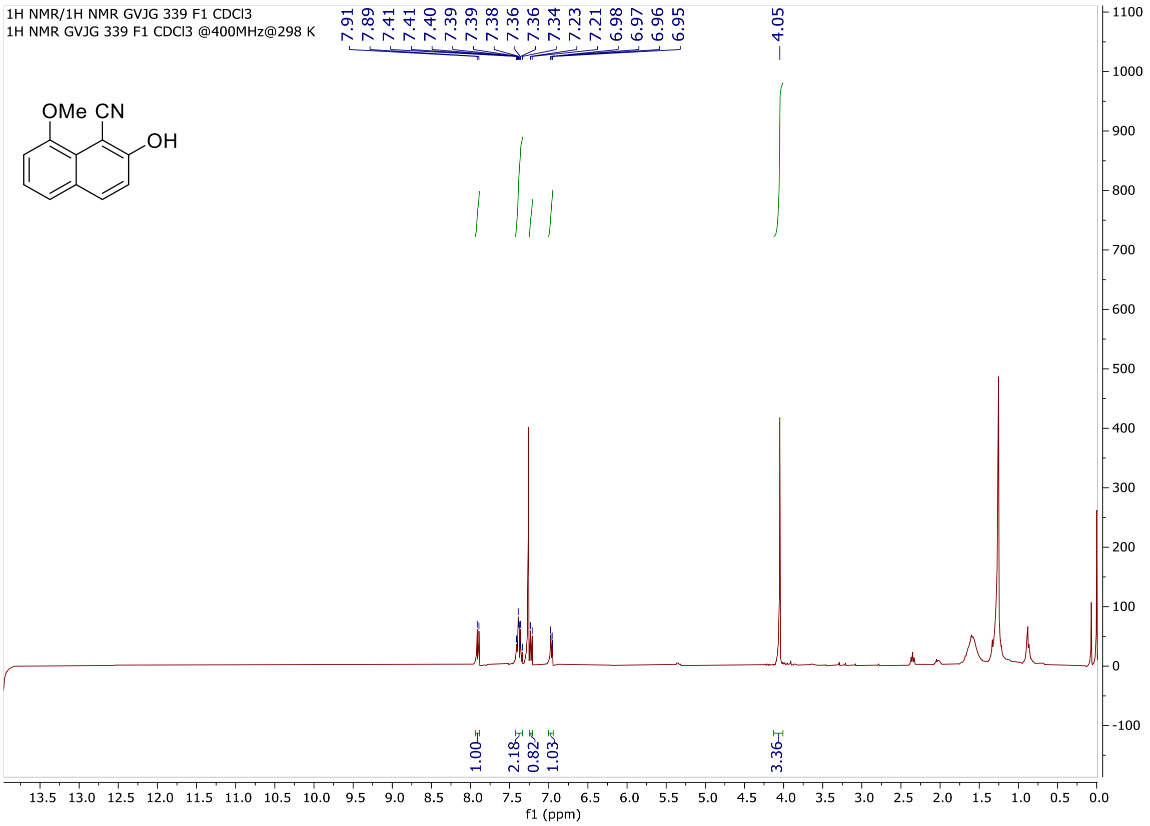

Figure S6. ^{1}H NMR (400 MHz, $CDCl_3$) of compound (**12**).

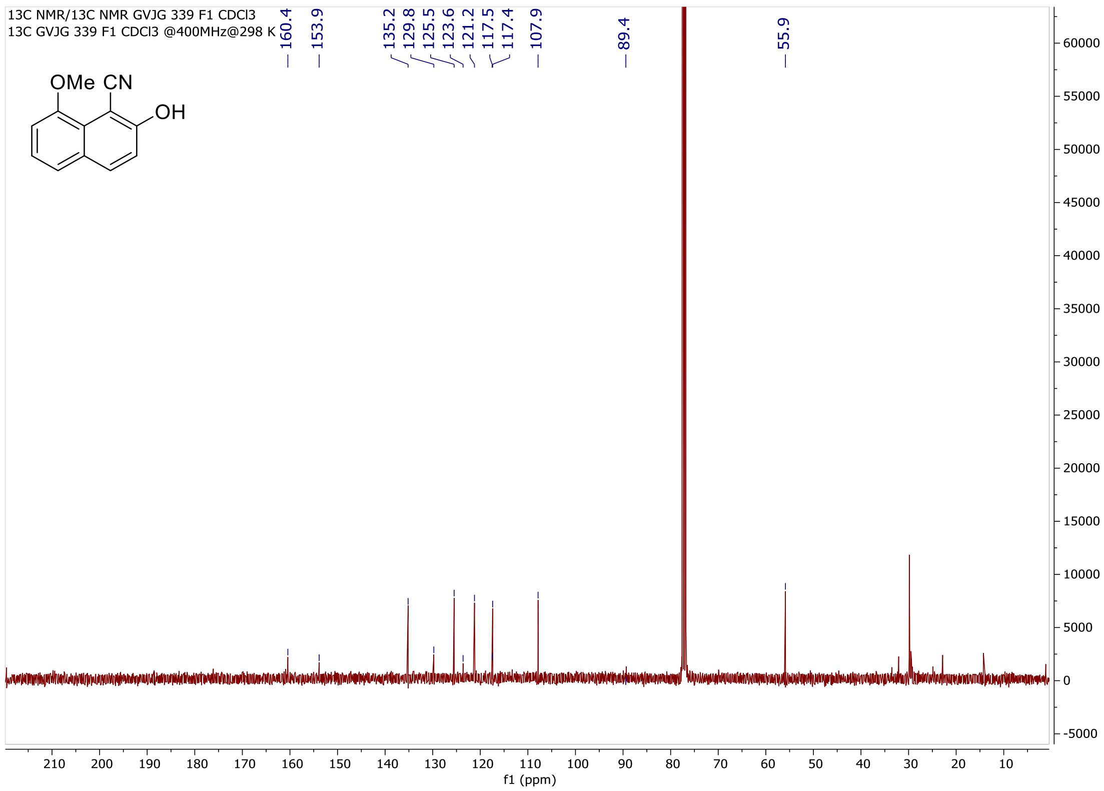

Figure S7. ^{13}C NMR (100.6 MHz, $CDCl_3$) of compound (**12**).

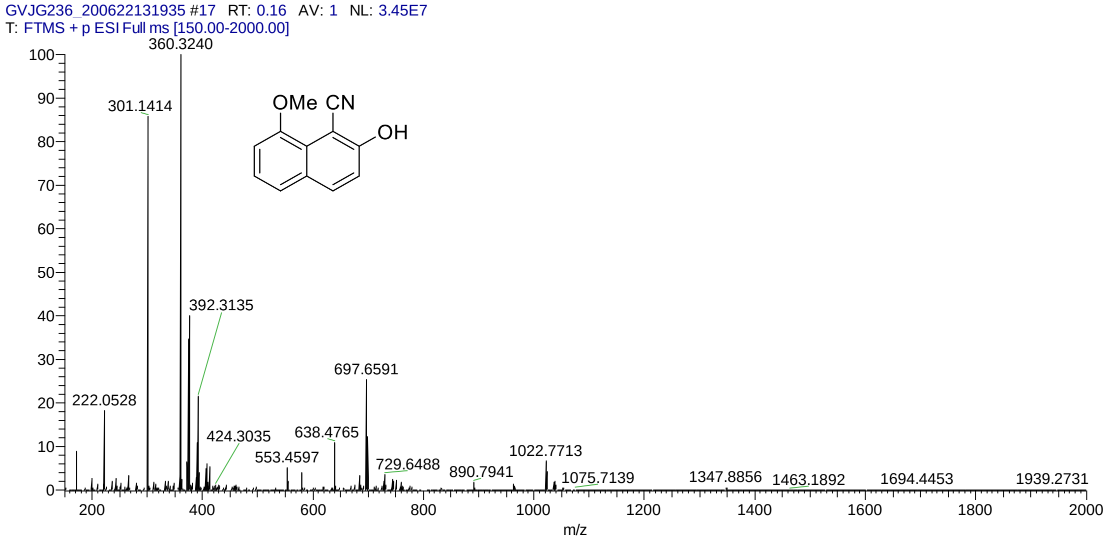

Figure S8. HRMS $[M+H]^+$ of compound (**12**) using 0.1% HCO_2H in MeOH as solvent.

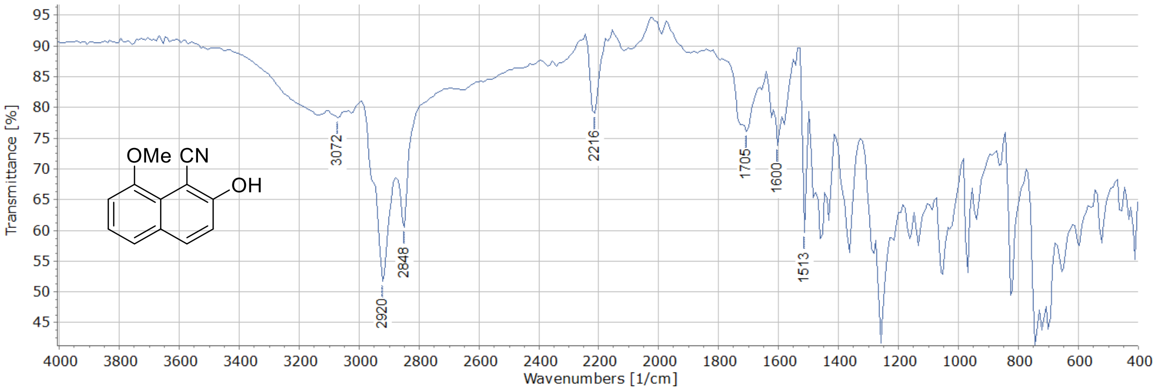

Figure S9. IR spectrum (solid) of compound (**12**).

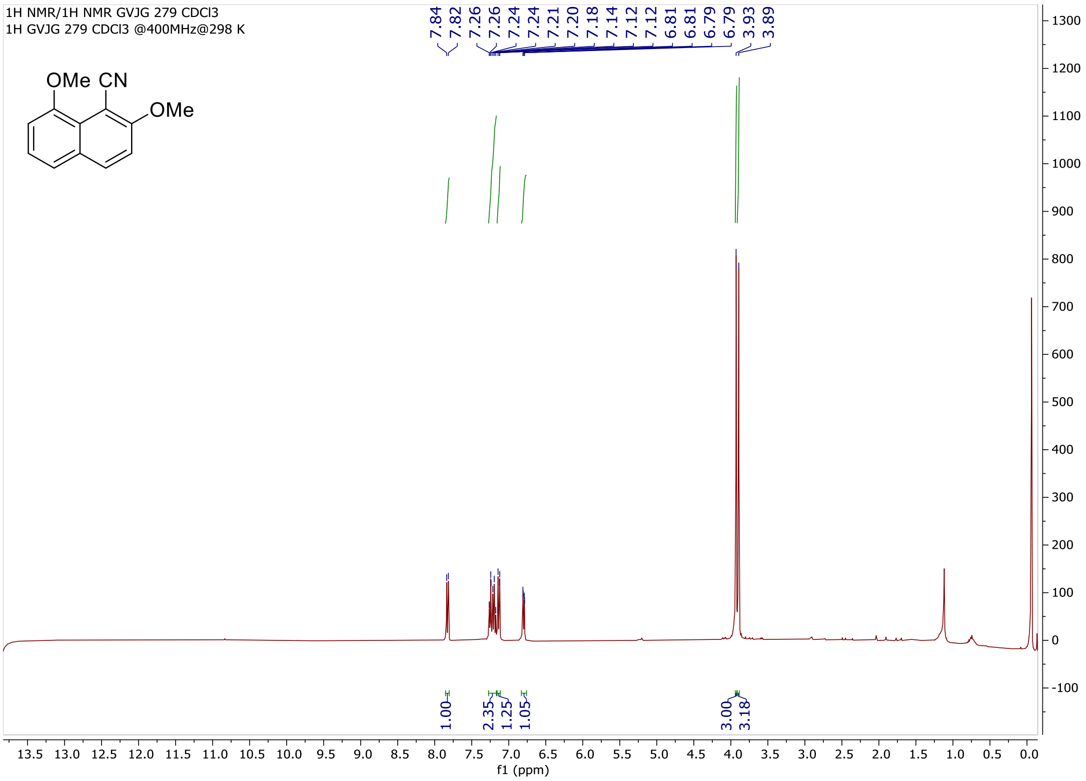

Figure S10. ^{1}H NMR (400 MHz, $CDCl_3$) of compound (**13**).

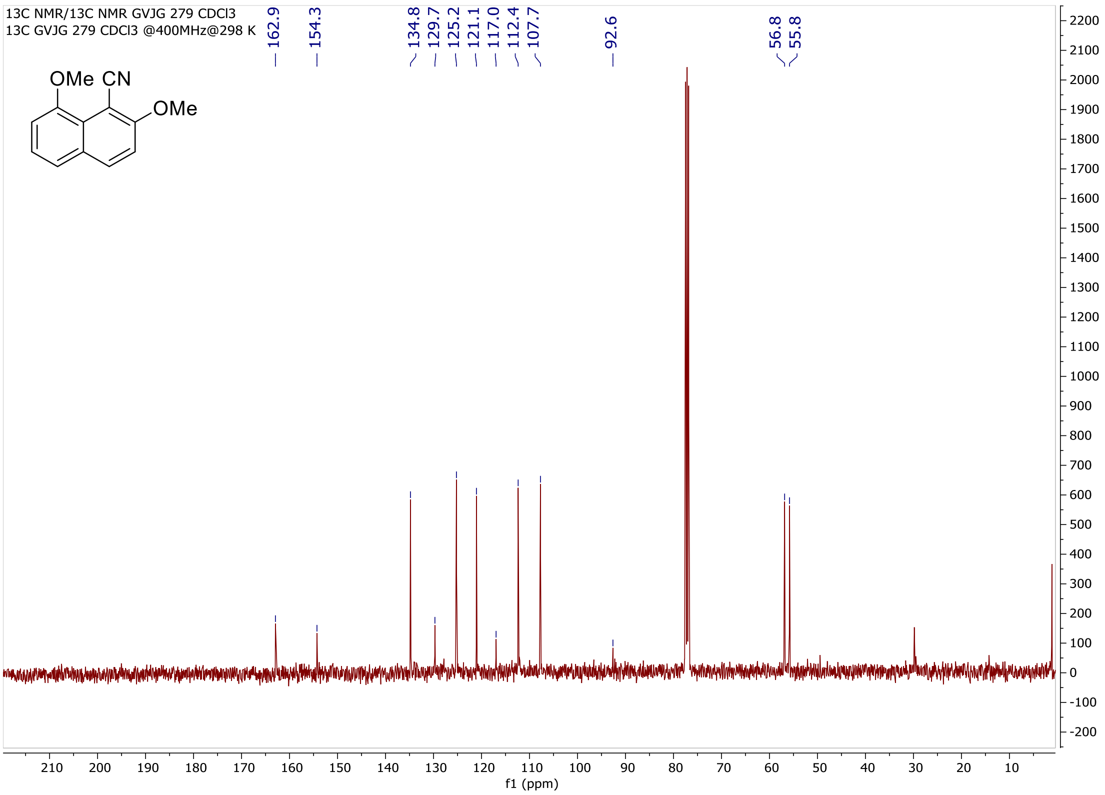

Figure S11. ^{13}C NMR (400 MHz, $CDCl_3$) of compound (**13**).

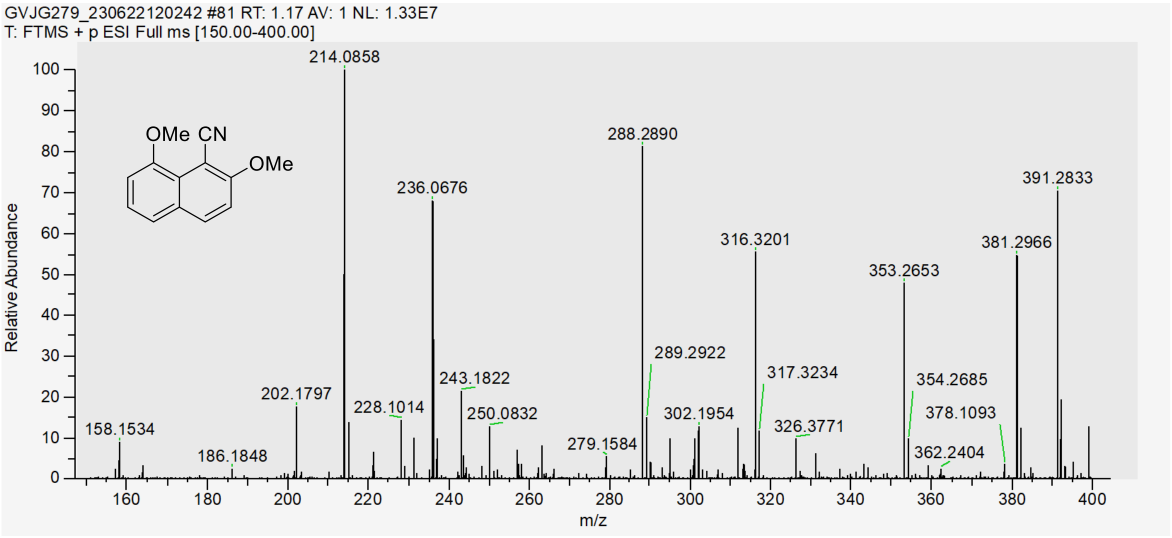

Figure S12. HRMS $[M+H]^+$ of compound (**13**) using 0.1% HCO_2H in MeOH as solvent.

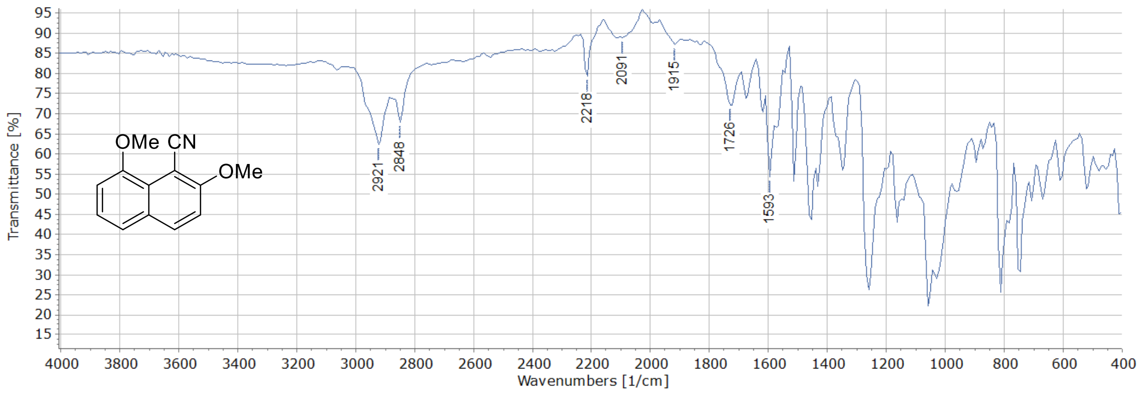

Figure S13. IR spectrum (solid) of compound (**13**).

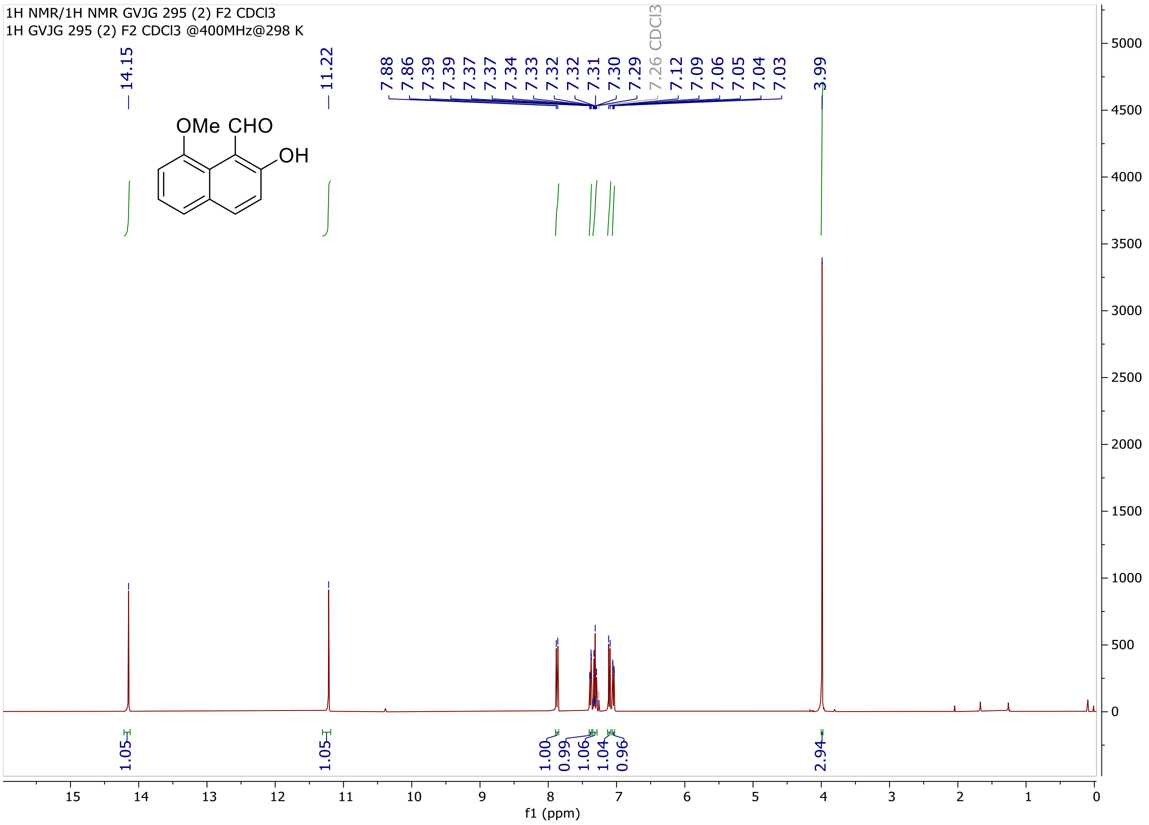

Figure S14. ^{1}H NMR (400 MHz, $CDCl_3$) of compound (**14**).

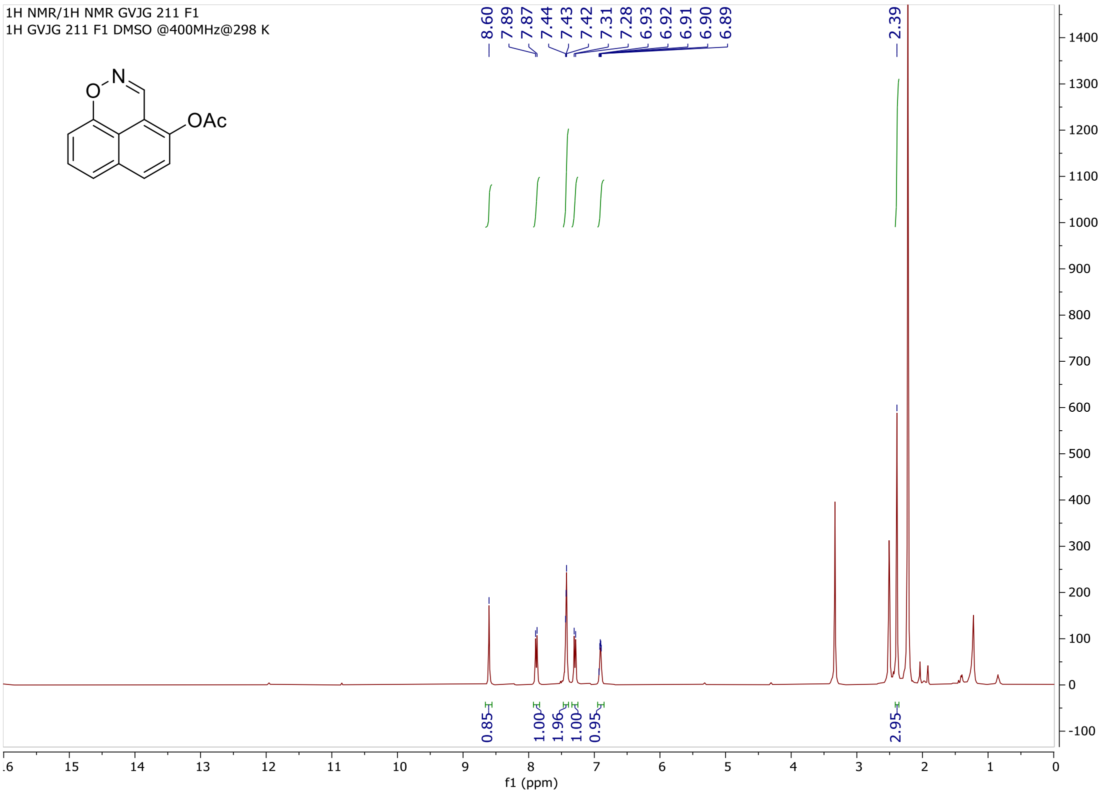

Figure S15. ^{1}H NMR (400 MHz, DMSO-d_6) of compound (**15**).

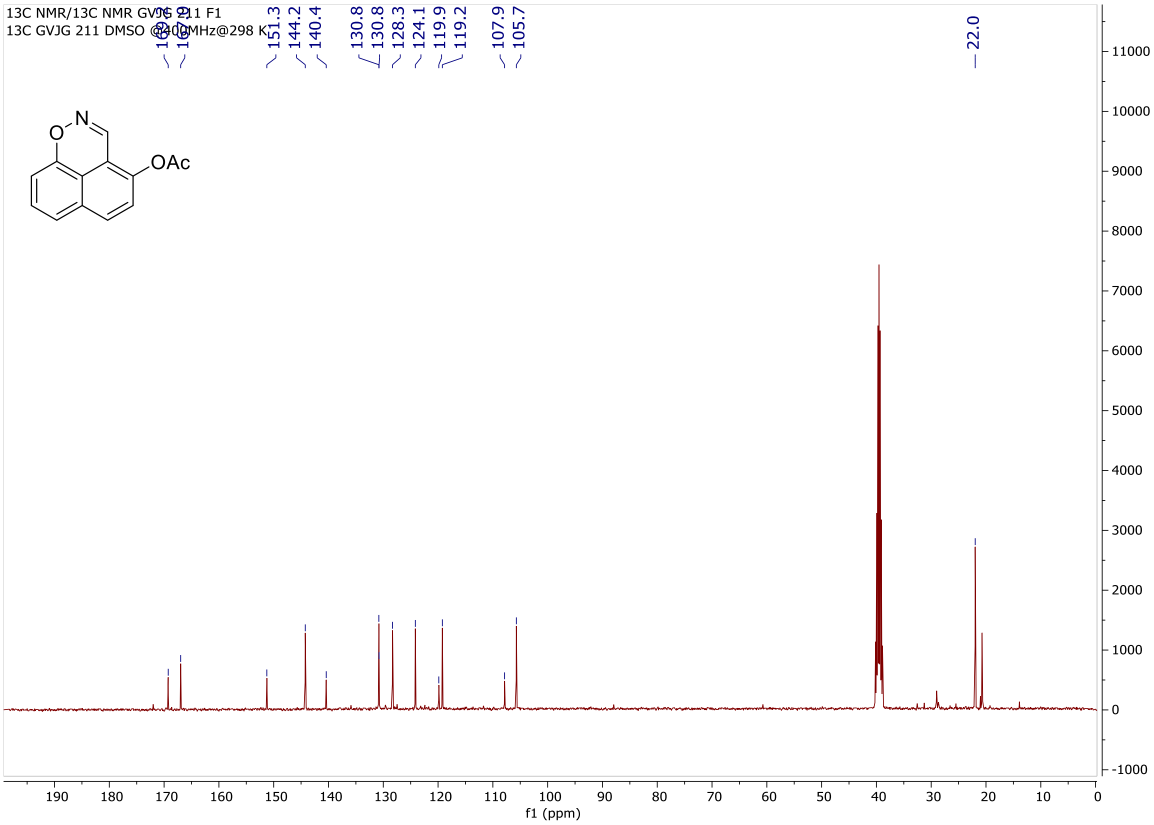

Figure S16. ^{13}C NMR (100.6 MHz, DMSO-d_6) of compound (**15**).

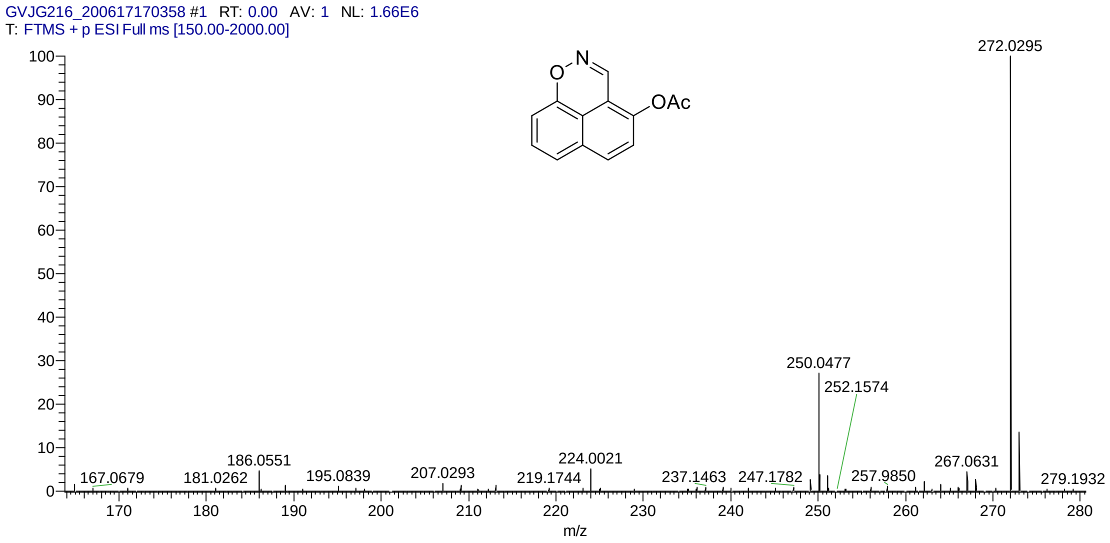

Figure S17. HRMS [M+H]$^+$ of compound (**15**) using 0.1% HCO_2H in MeOH as solvent.

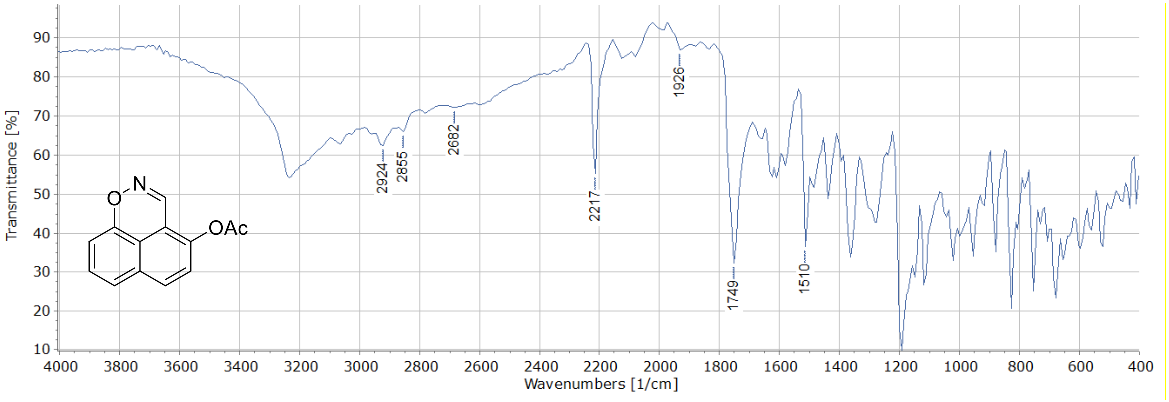

Figure S18. IR spectrum (solid) of compound (**15**).

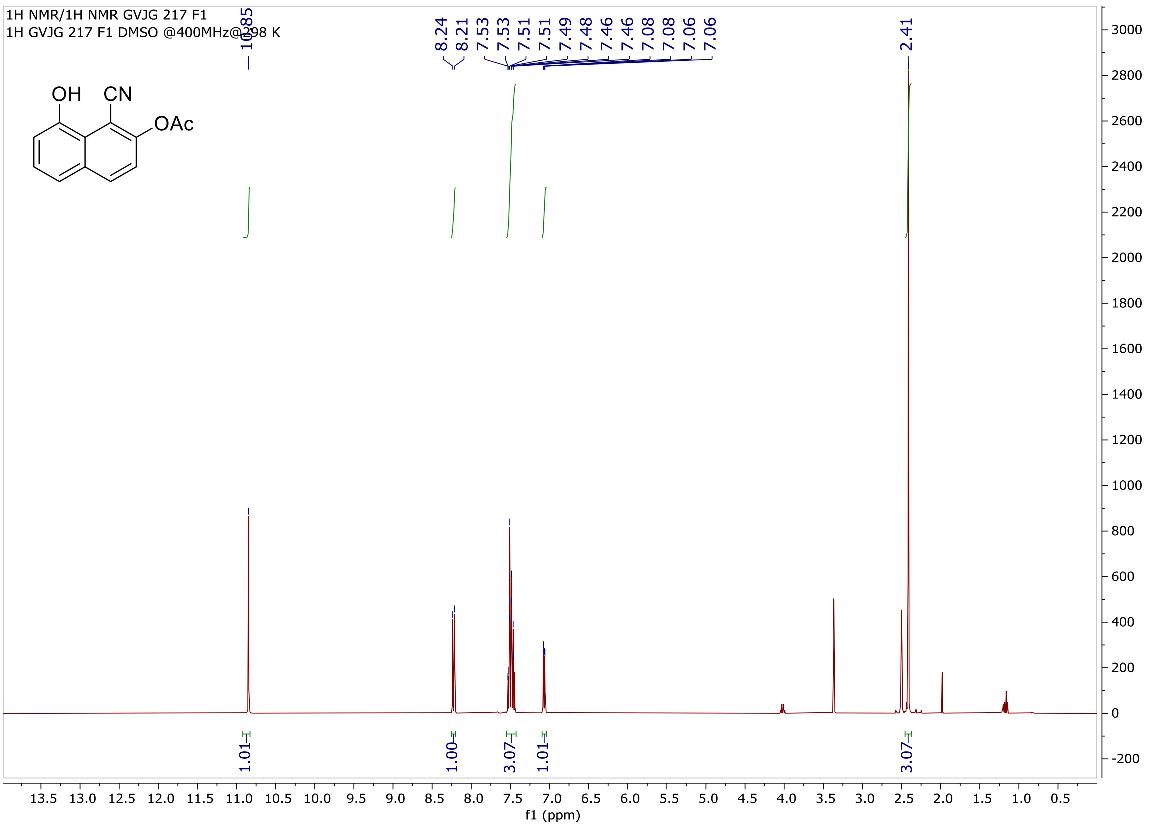

Figure S19. ^{1}H NMR (400 MHz, DMSO-d_6) of compound (**16**).

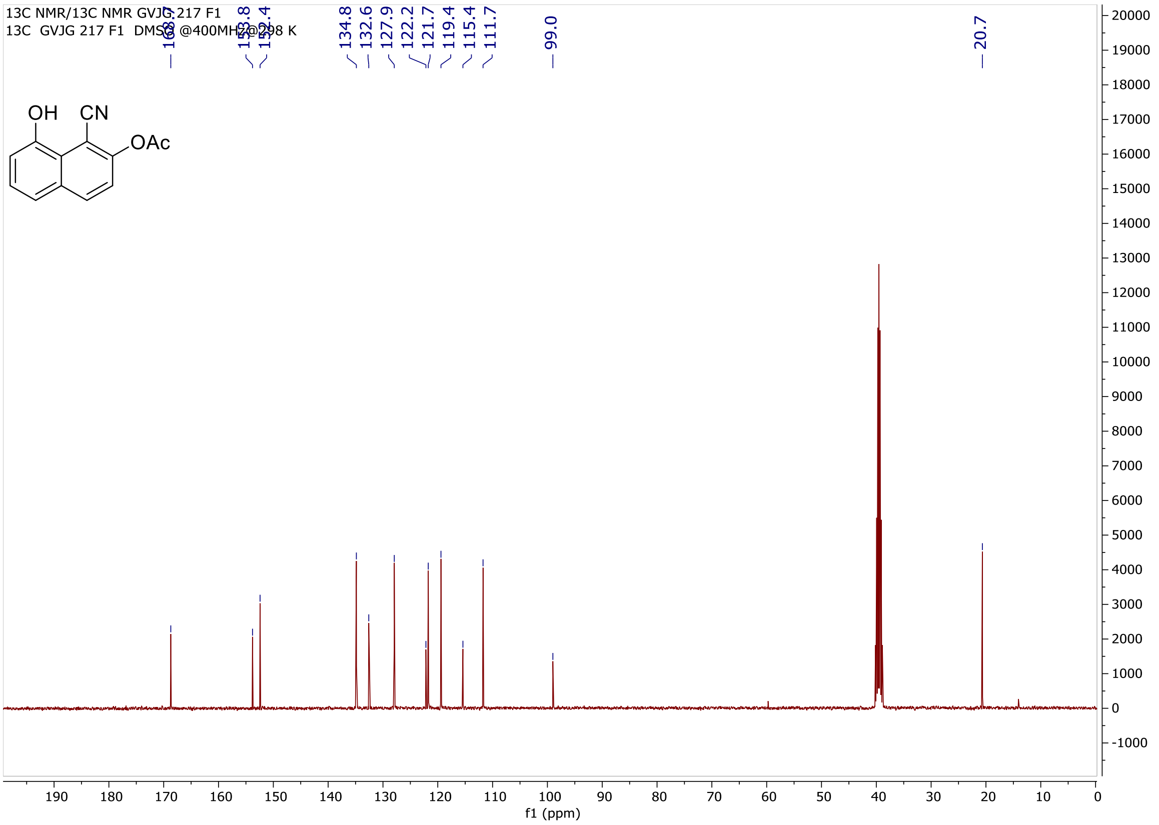

Figure S20. ^{13}C NMR (100.6 MHz, DMSO-d_6) of compound (**16**).

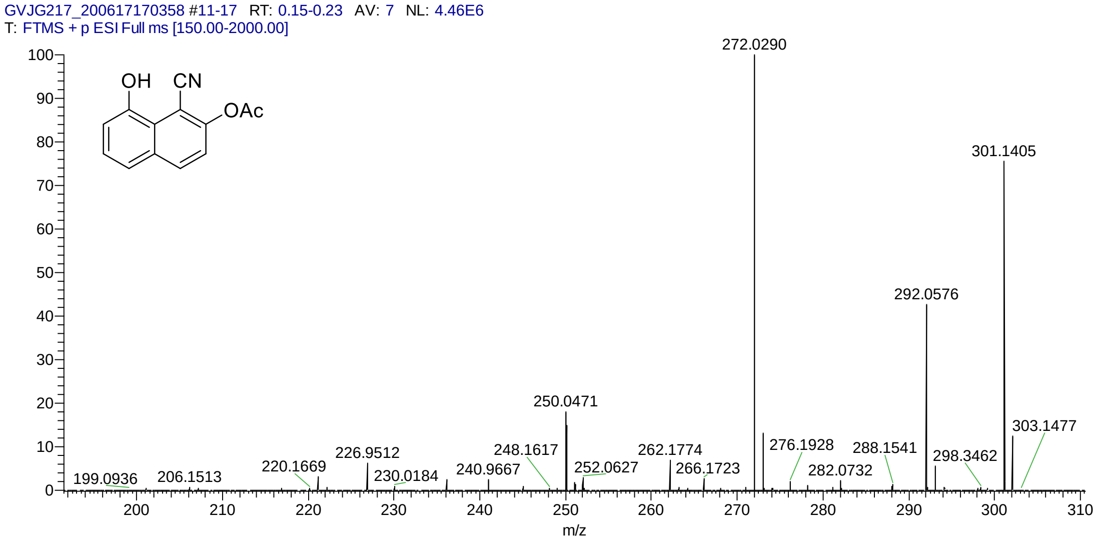

Figure S21. HRMS $[M+H]^+$ of compound (**16**) using 0.1% HCO_2H in MeOH as solvent.

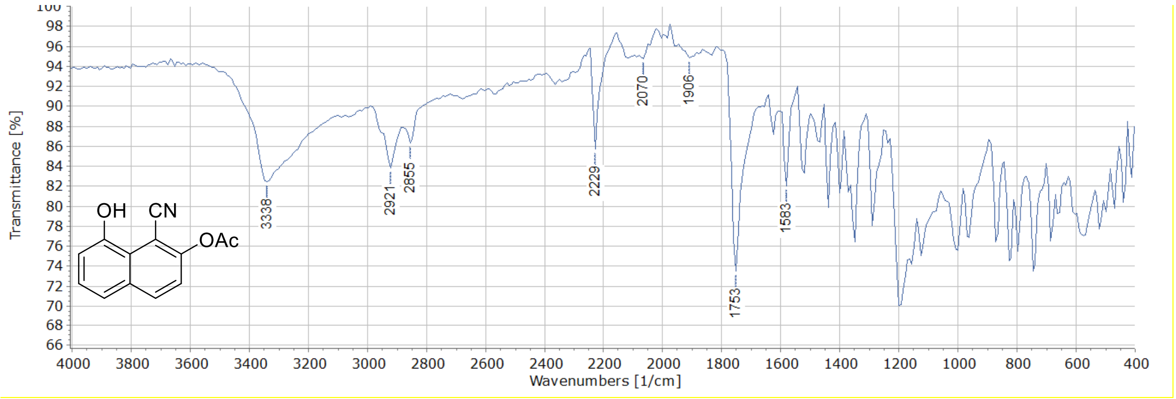

Figure S22. IR spectrum (solid) of compound (**16**).

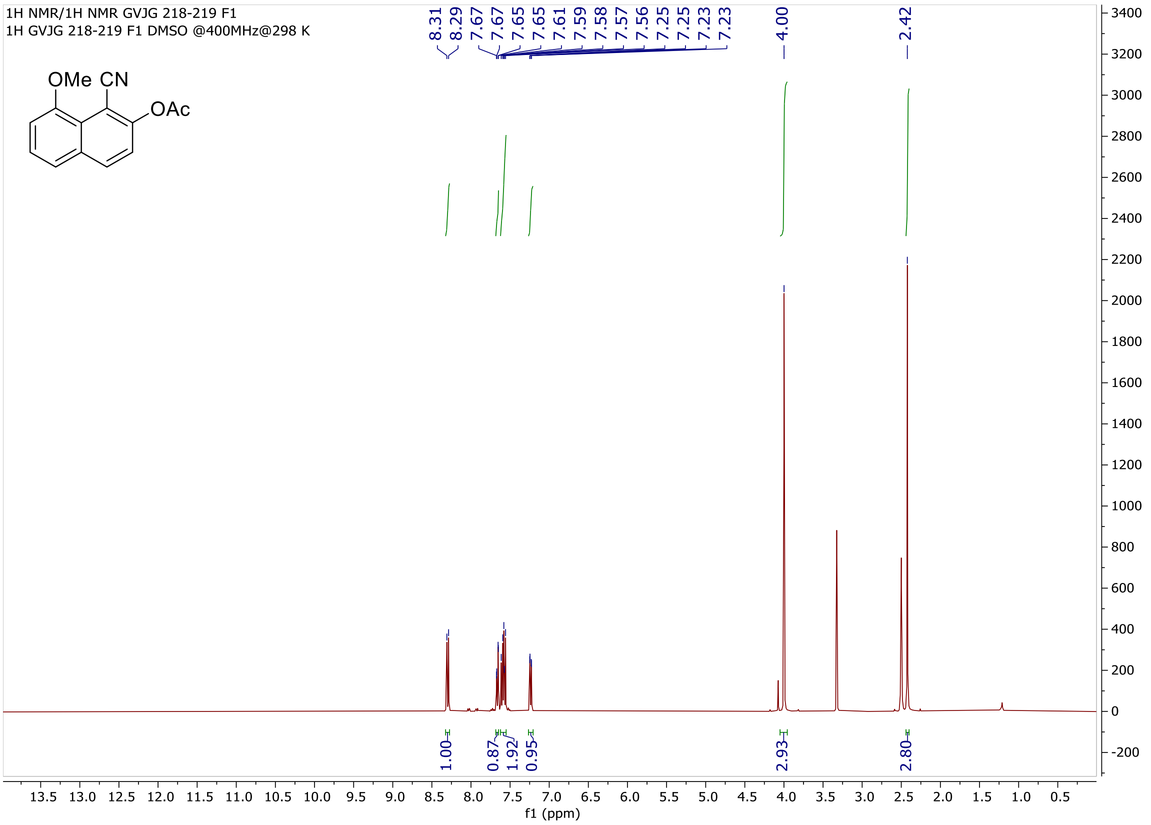

Figure S23. ^{1}H NMR (400 MHz, DMSO-d_6) of (**17**).

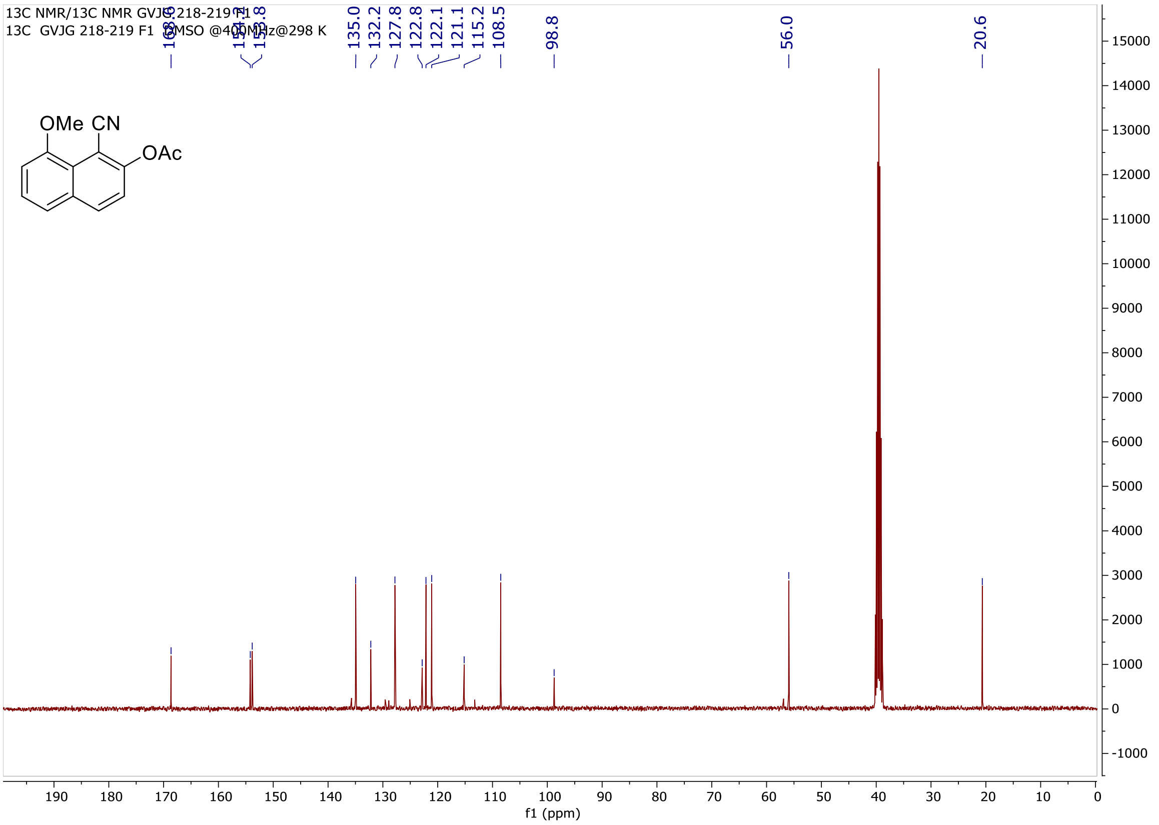

Figure S24. ^{13}C NMR (100.6 MHz, DMSO-d_6) of (**17**).

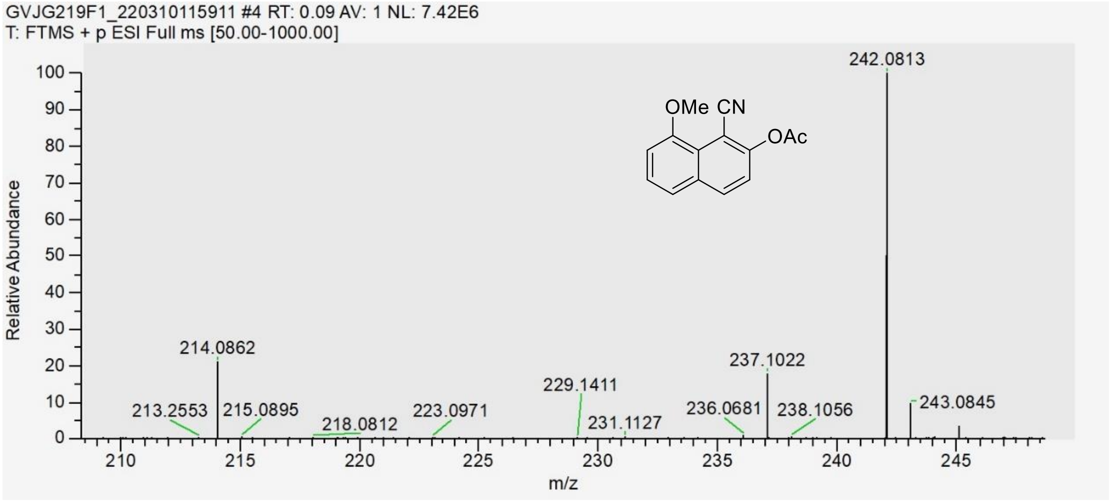

Figure S25. HRMS [M+H]$^+$ of compound (**17**) using 0.1% HCO_2H in MeOH as solvent.

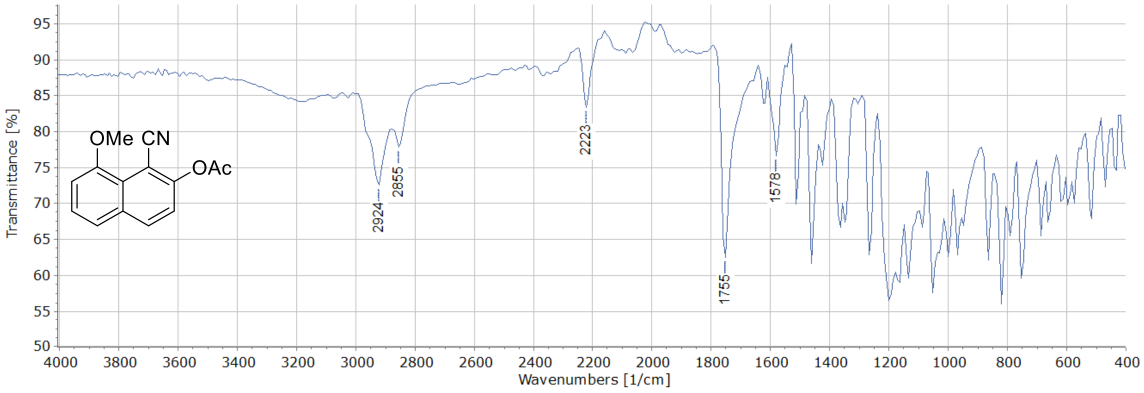

Figure S26. IR spectrum (solid) of (**17**)

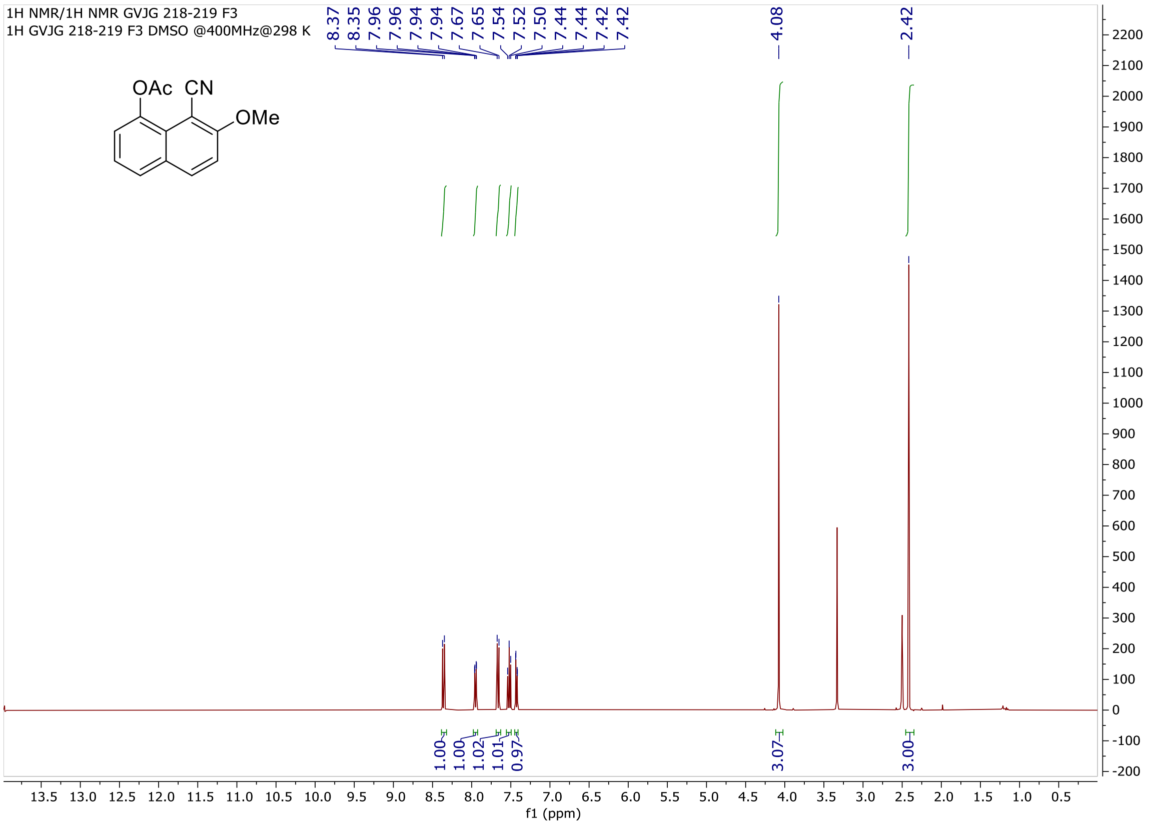

Figure S27. ^{1}H NMR (400 MHz, DMSO-d_6) of (**18**).

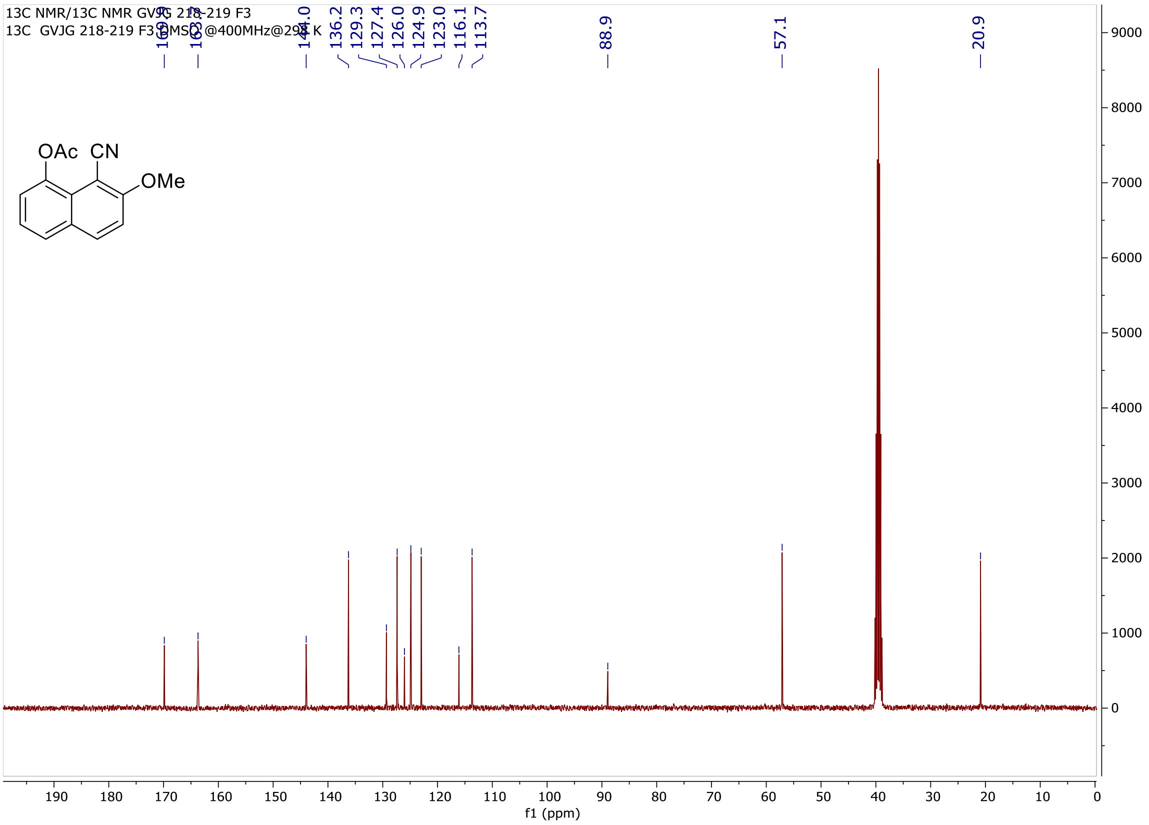

Figure S28. ^{13}C NMR (100.6 MHz, DMSO-d_6) of (**18**).

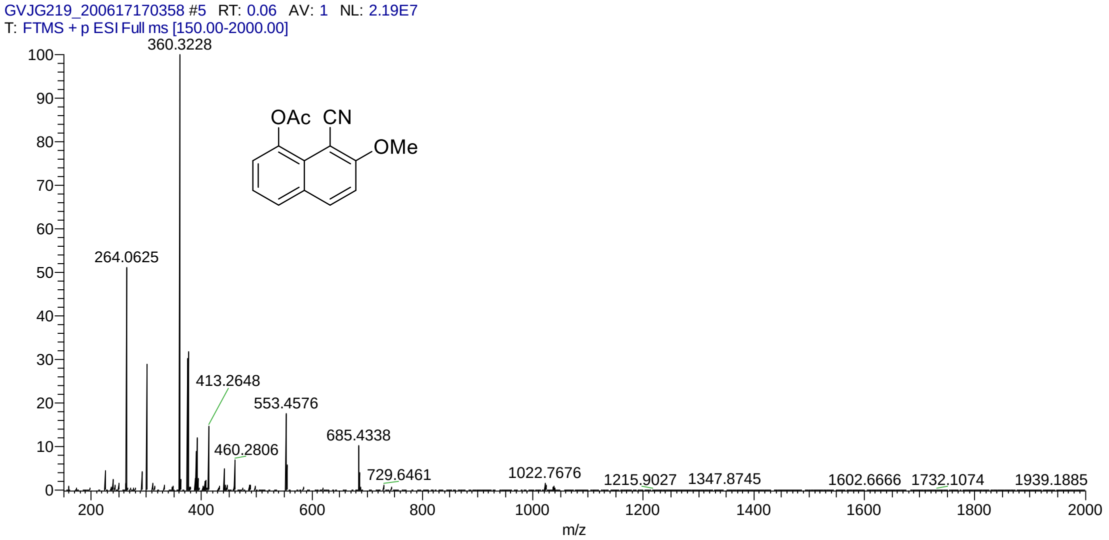

Figure S29. HRMS [M+H]$^+$ of compound (**18**) using 0.1% HCO_2H in MeOH as solvent.

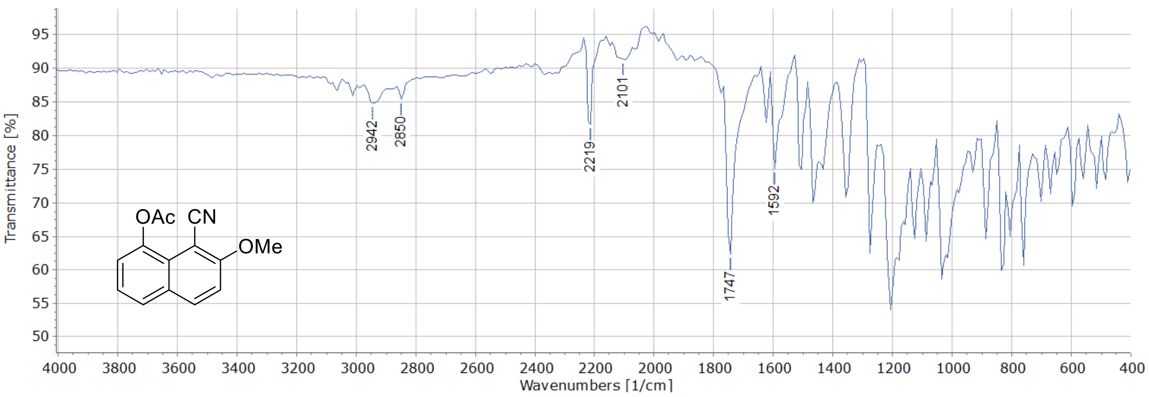

Figure S30. IR spectrum (solid) of (**18**).

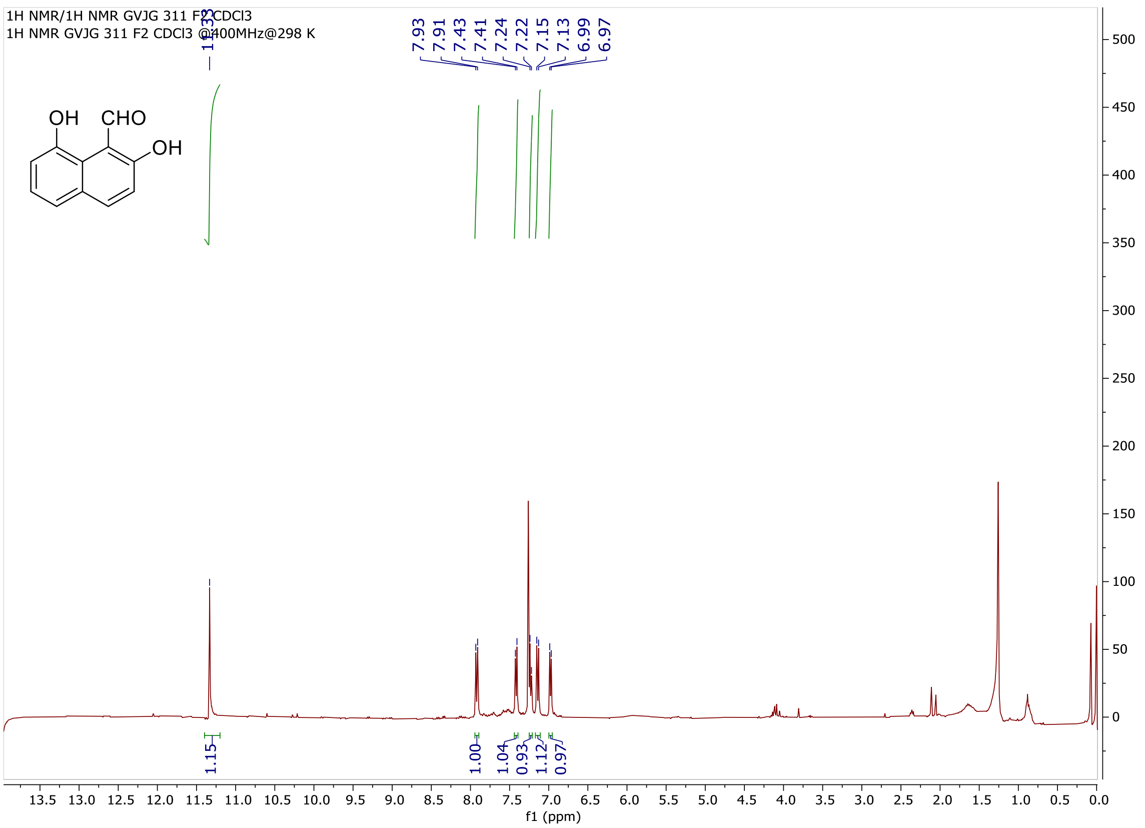

Figure S31. ^{1}H NMR (400 MHz, $CDCl_3$) of (**19**).

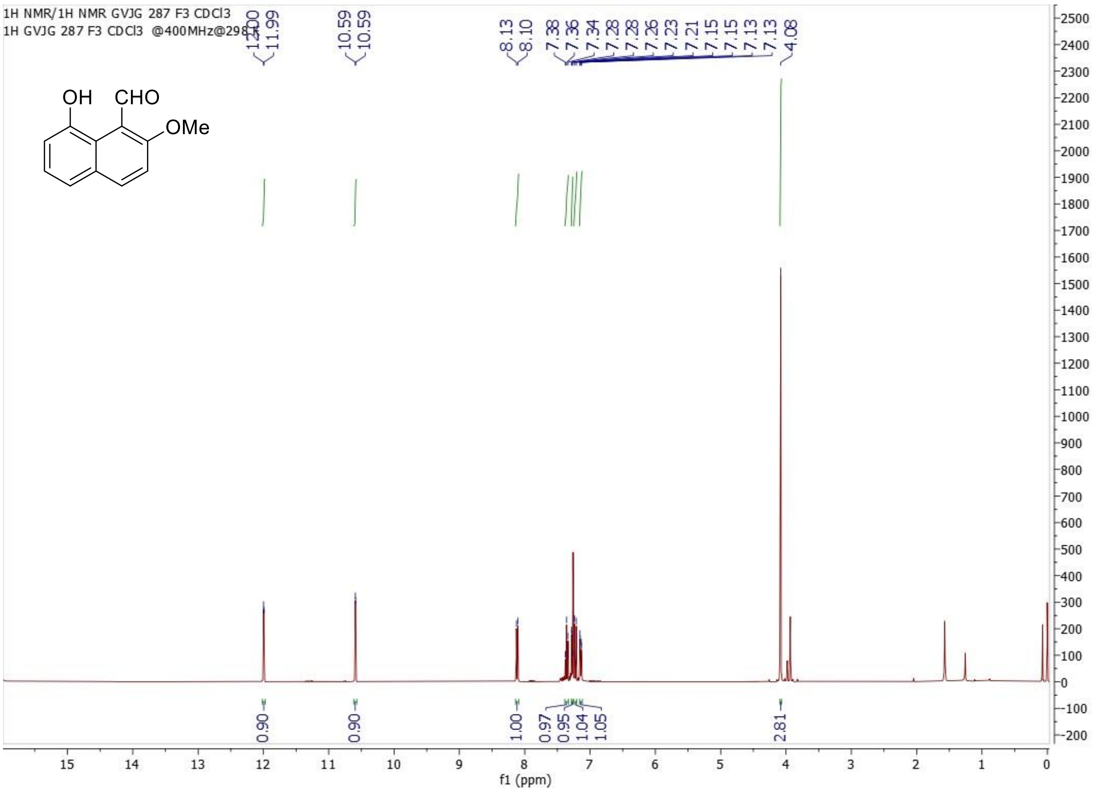

Figure S32. ^{1}H NMR (400 MHz, $CDCl_3$) of (**20**).

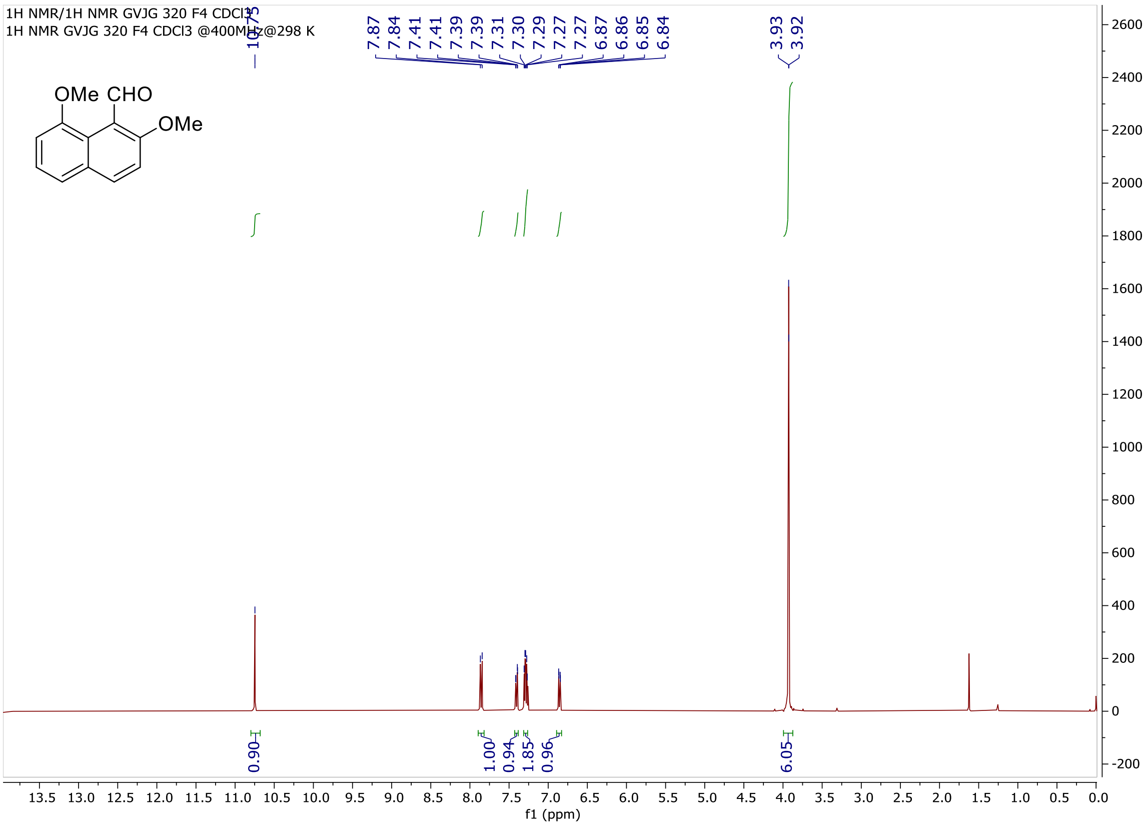

Figure S33. ^{1}H NMR (400 MHz, $CDCl_3$) of (**21**).

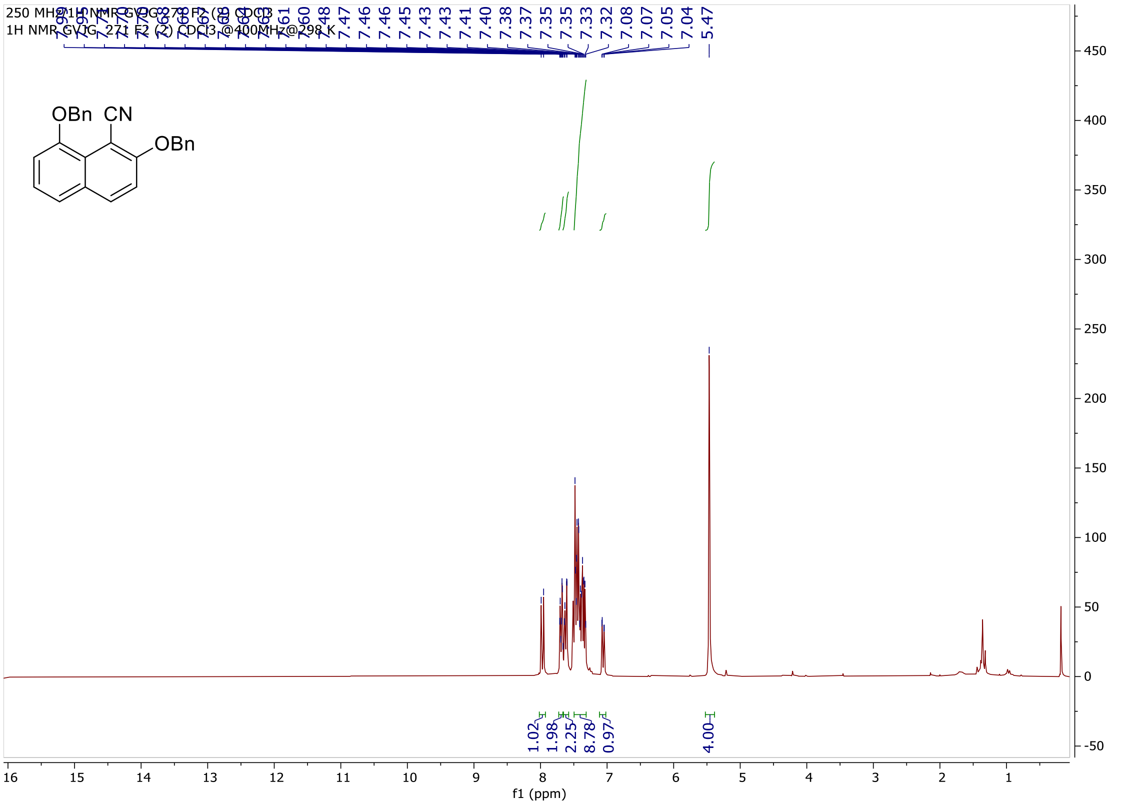

Figure S34. ^{1}H NMR (400 MHz, $CDCl_3$) of (**22**).

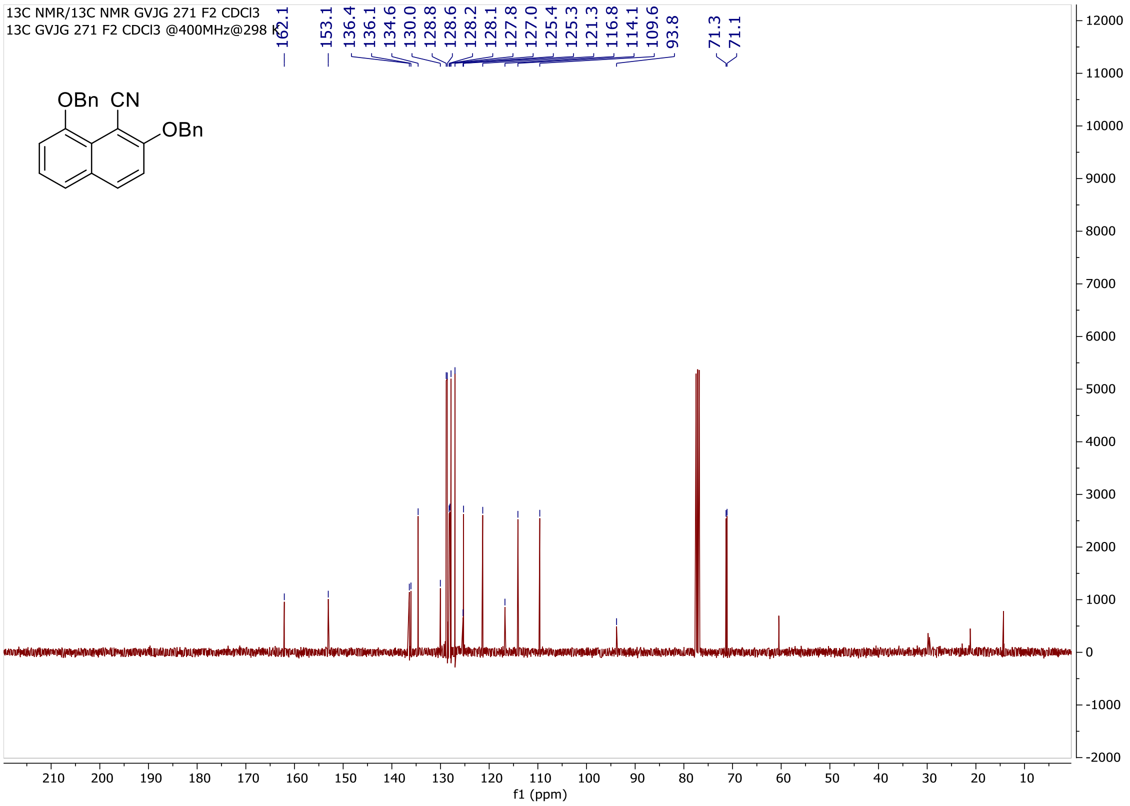

Figure S35. ^{13}C NMR (100.6 MHz, $CDCl_3$) of (**22**).

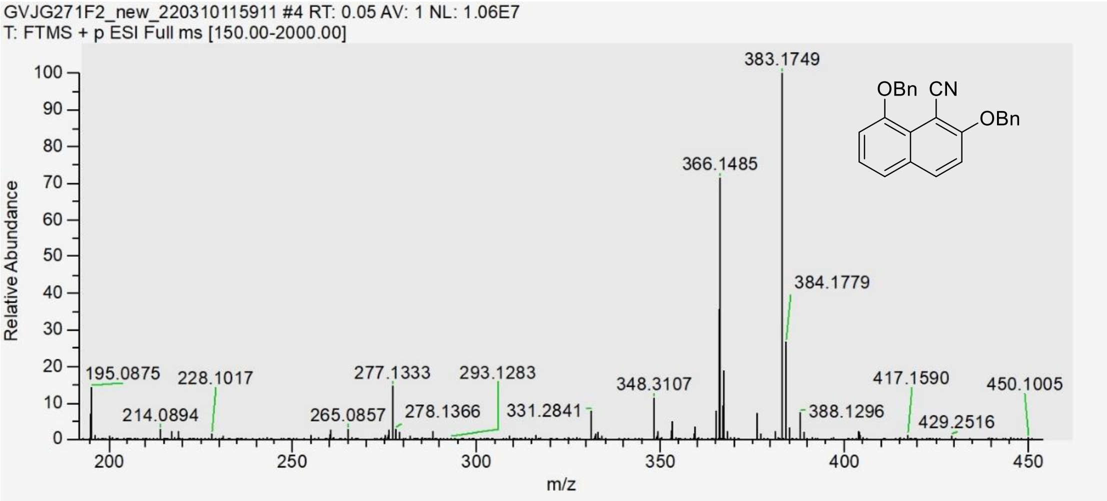

Figure S36. HRMS [M+H]+ of compound (**22**) using 0.1% HCO_2H in MeOH as solvent.

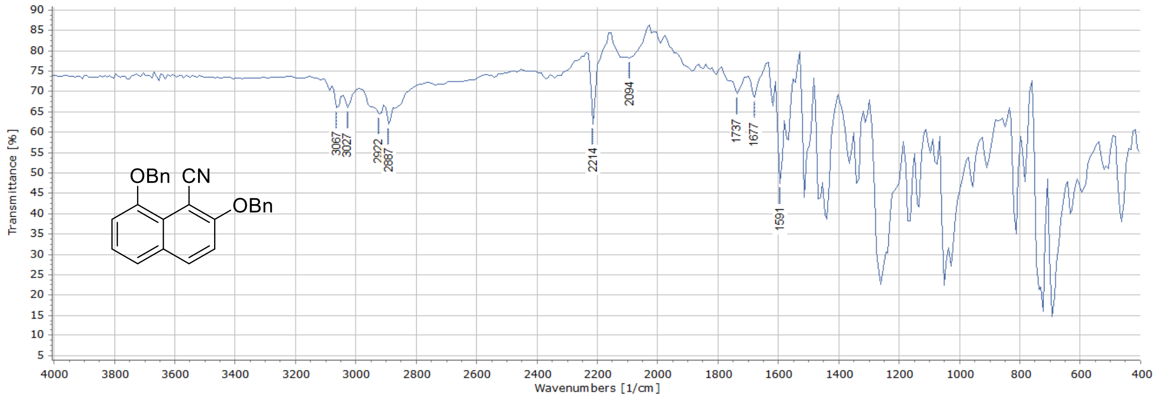

Figure S37. IR spectrum (solid) of (**22**).

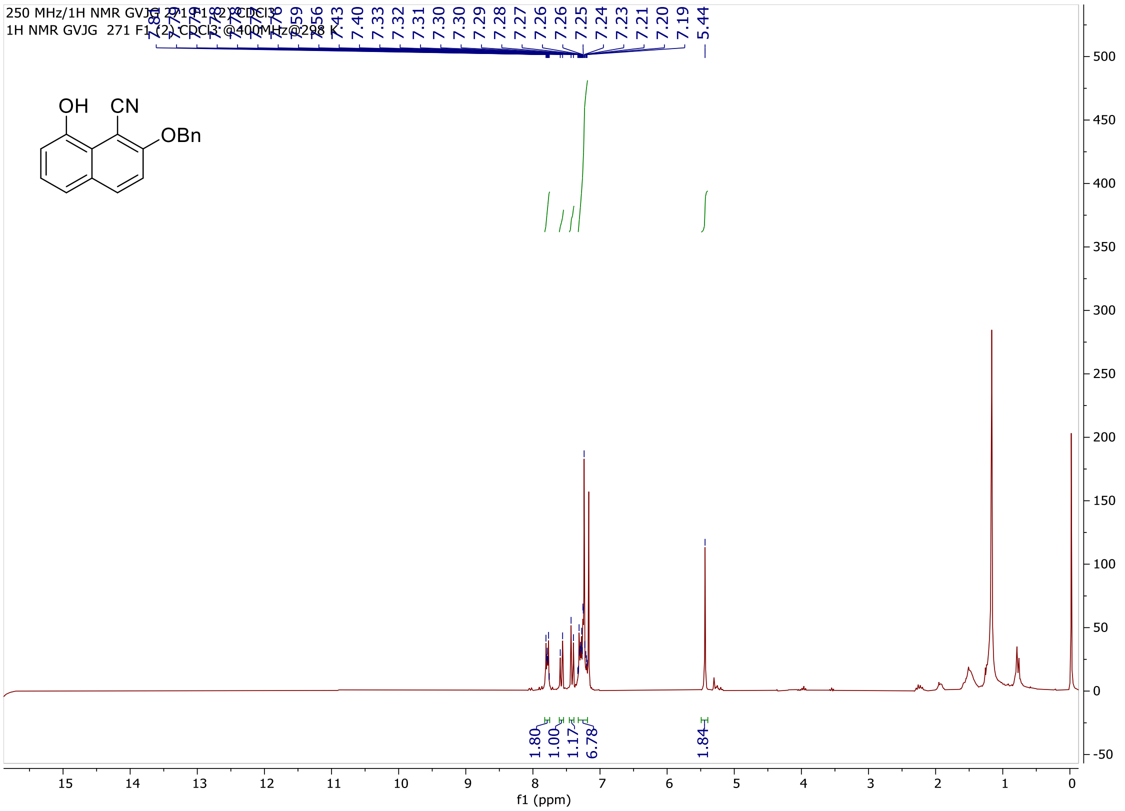

Figure S38. ^{1}H NMR (400 MHz, $CDCl_3$) of (**23**).

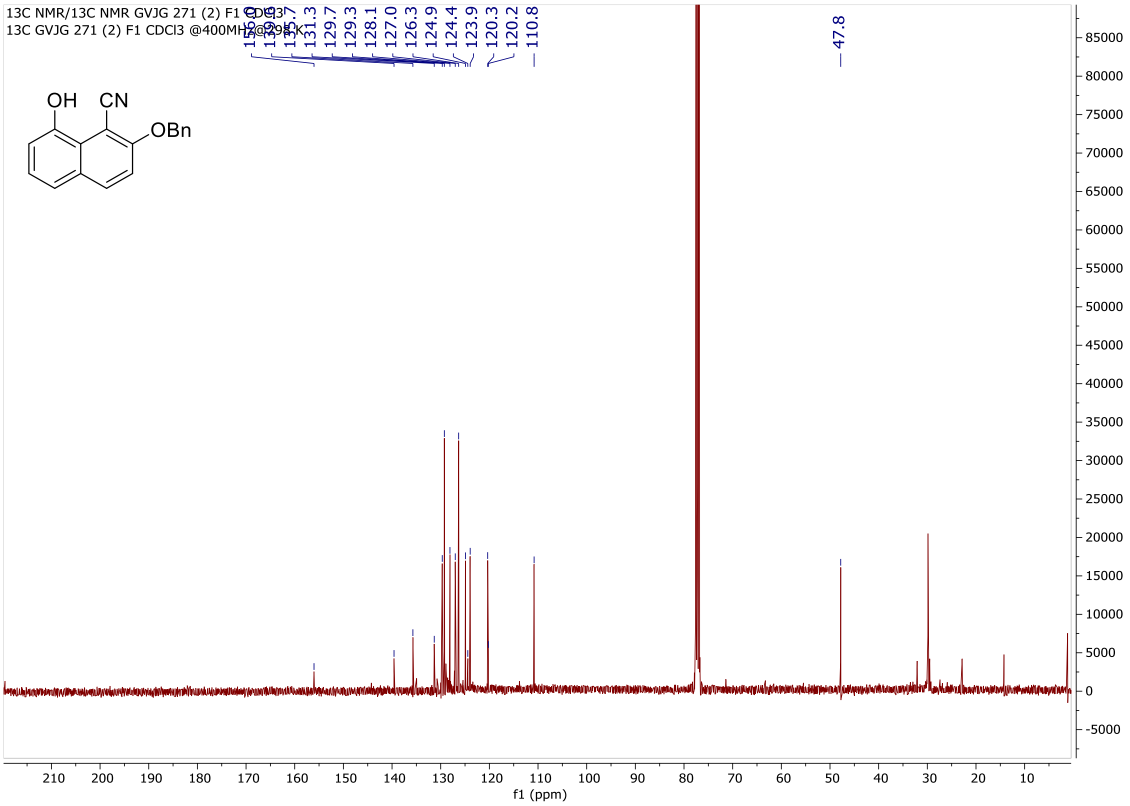

Figure S39. ^{13}C NMR (100.6 MHz, $CDCl_3$) of (**23**).

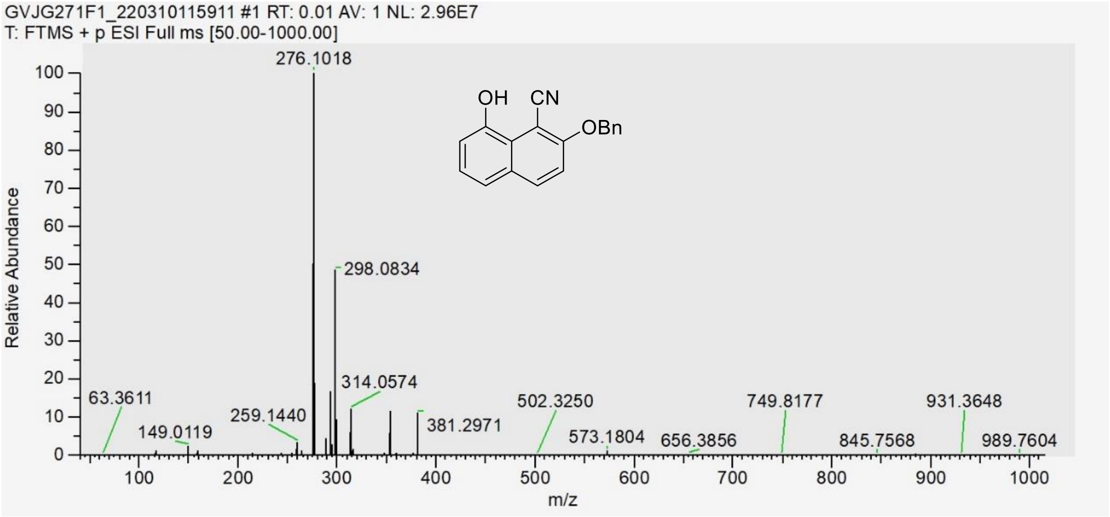

Figure S40. HRMS $[M+H]^+$ of compound (**23**) using 0.1% HCO_2H in MeOH as solvent.

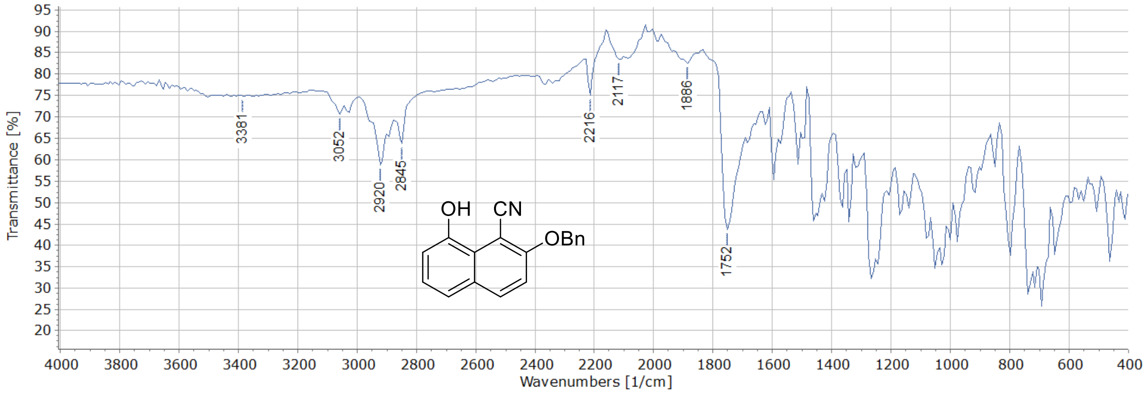

Figure S41. IR spectrum (solid) of (**23**).

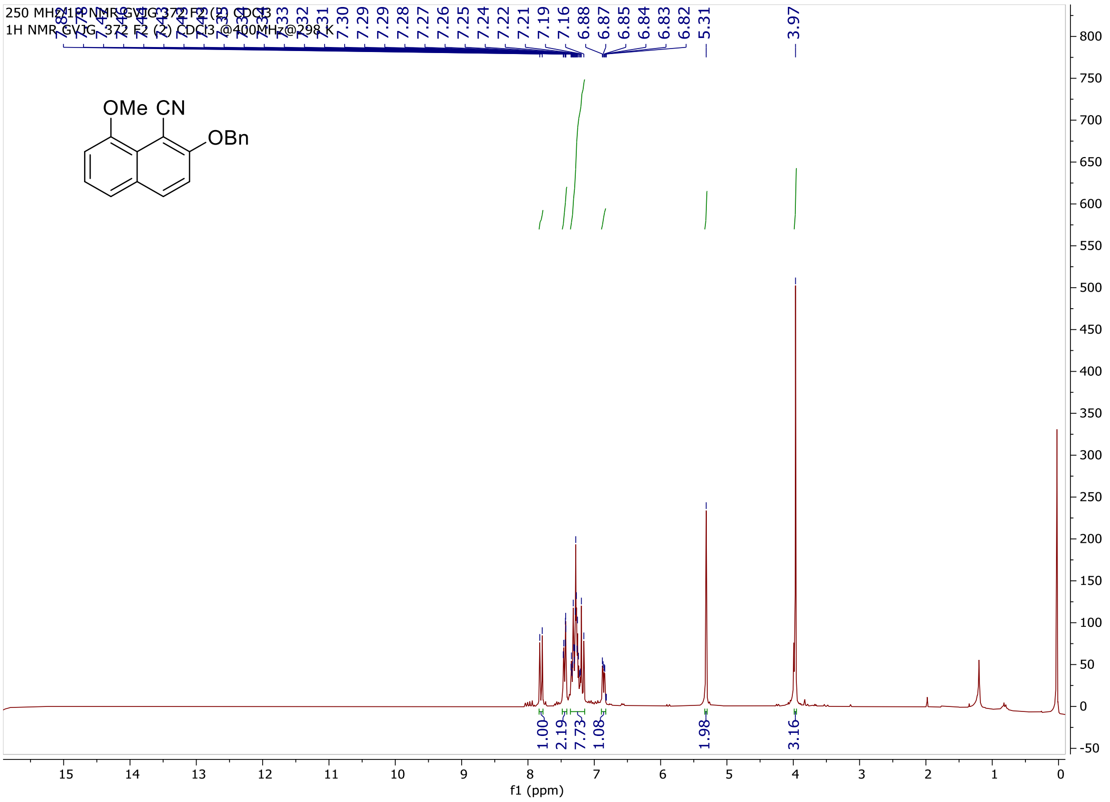

Figure S42. ^{1}H NMR (400 MHz, $CDCl_3$) of (**24**).

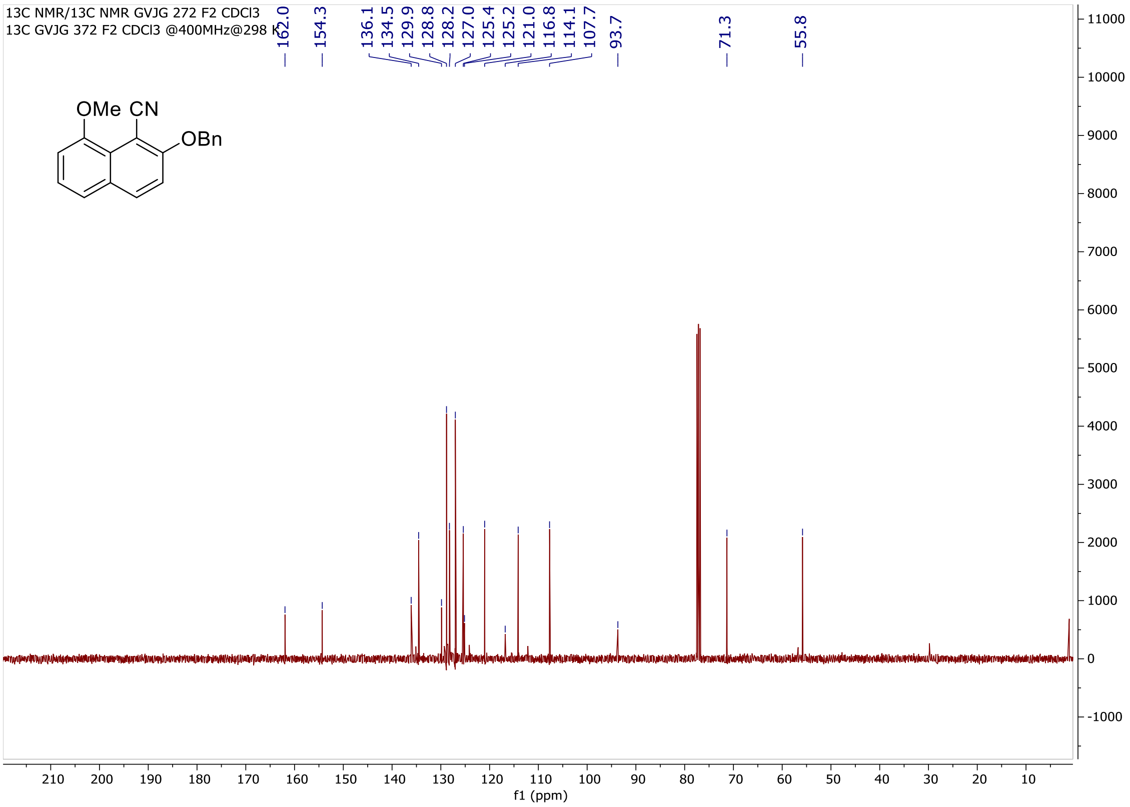

Figure S43. ^{13}C NMR (100.6 MHz, $CDCl_3$) of (**24**).

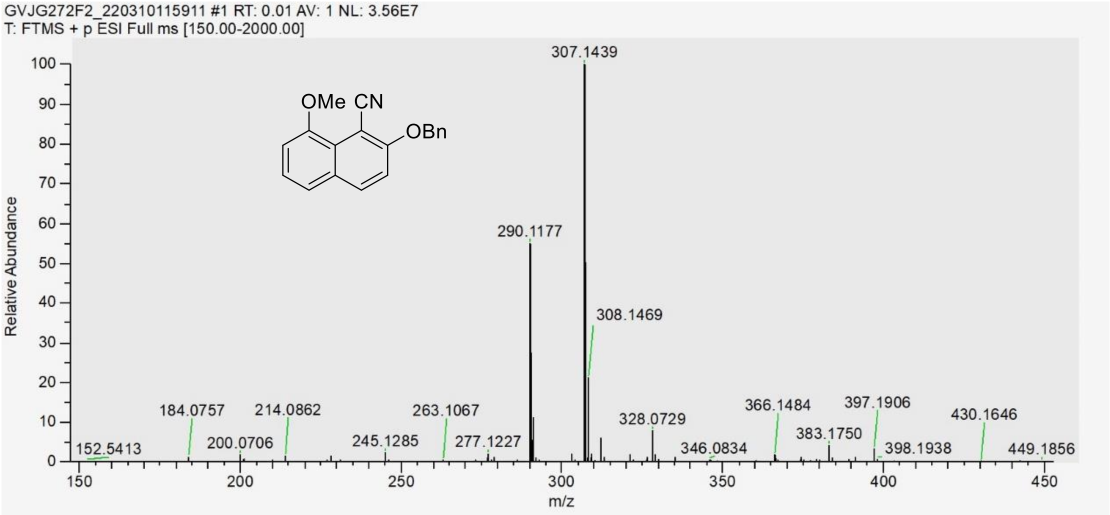

Figure S44. HRMS $[M+H]^+$ of compound (**24**) using 0.1% HCO_2H in MeOH as solvent.

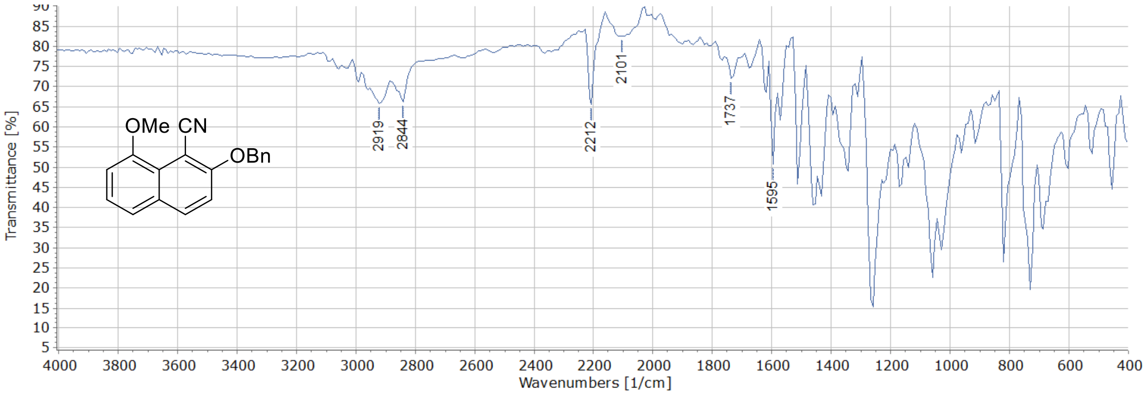

Figure S45. IR spectrum (solid state) of (**24**).

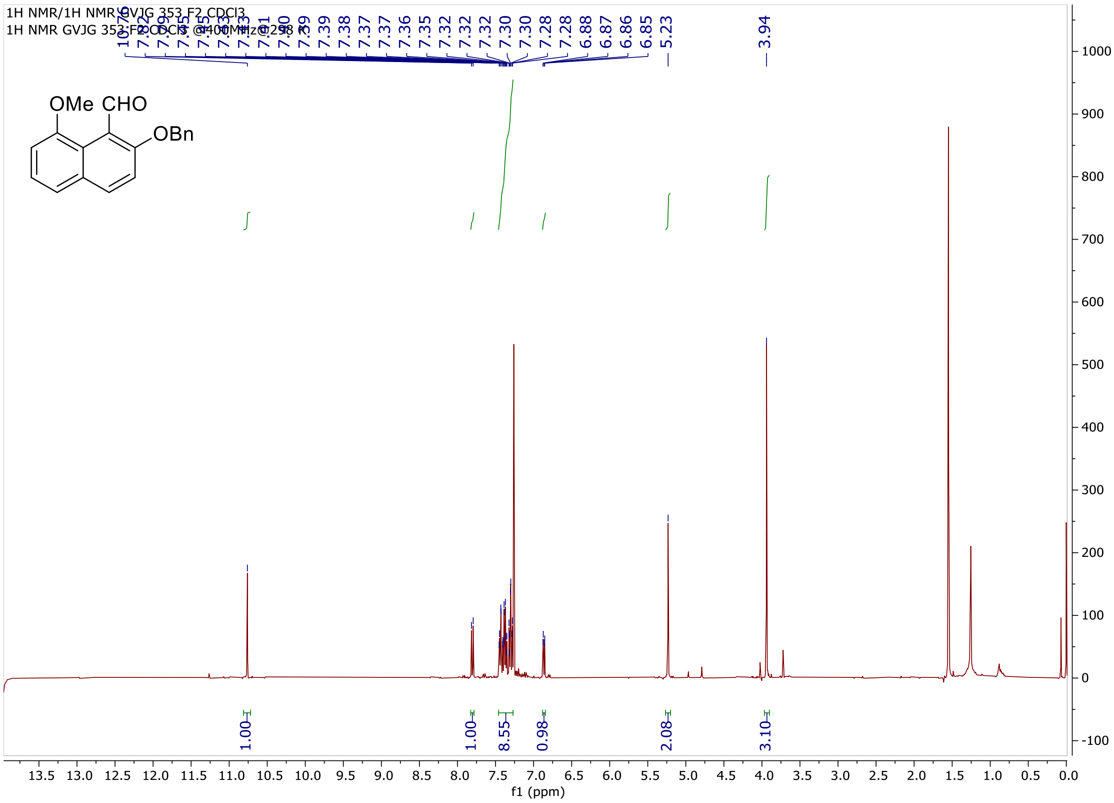

Figure S46. ^{1}H NMR (400 MHz, $CDCl_3$) of (**25**).

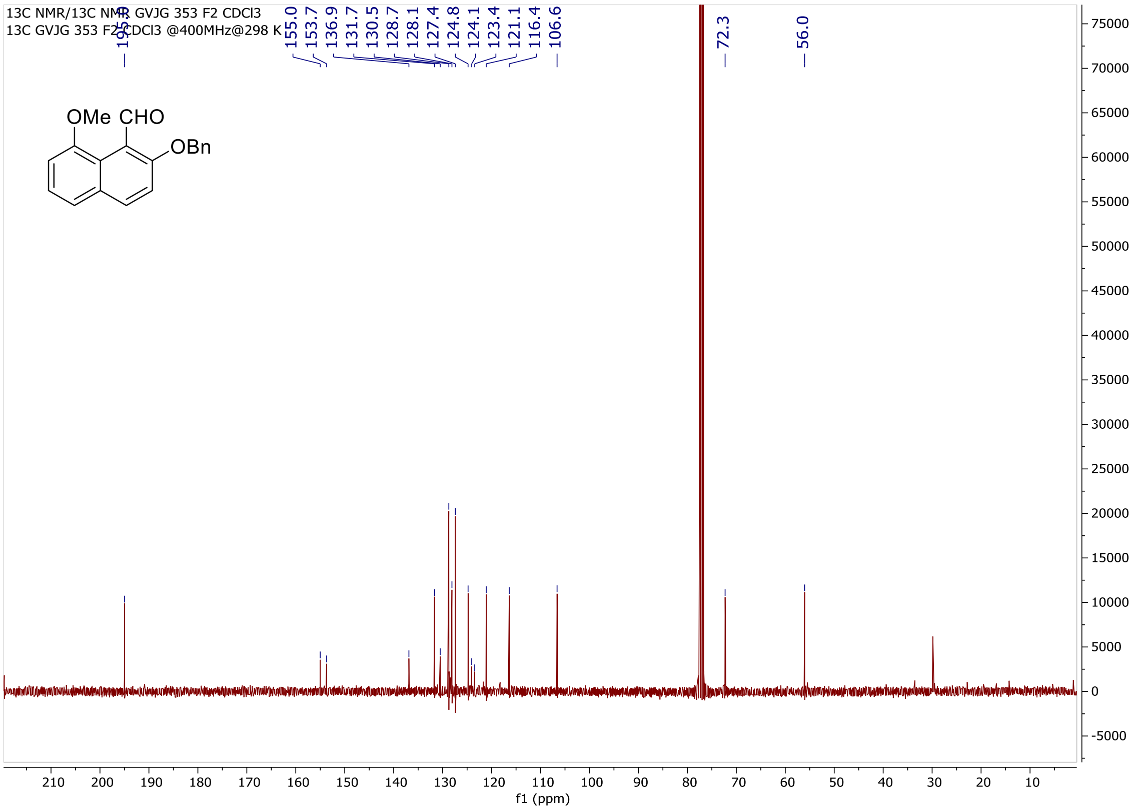

Figure S47. ^{13}C NMR [100.6 MHz, $CDCl_3$] of (**25**).

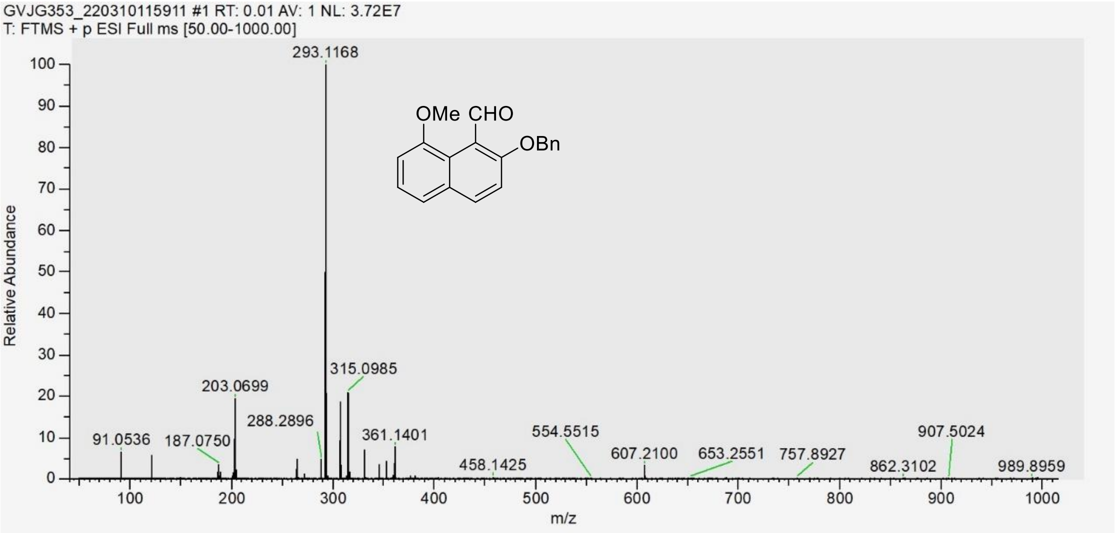

Figure S48. HRMS $[M+H]^+$ of compound (**25**) using 0.1% HCO_2H in MeOH as solvent.

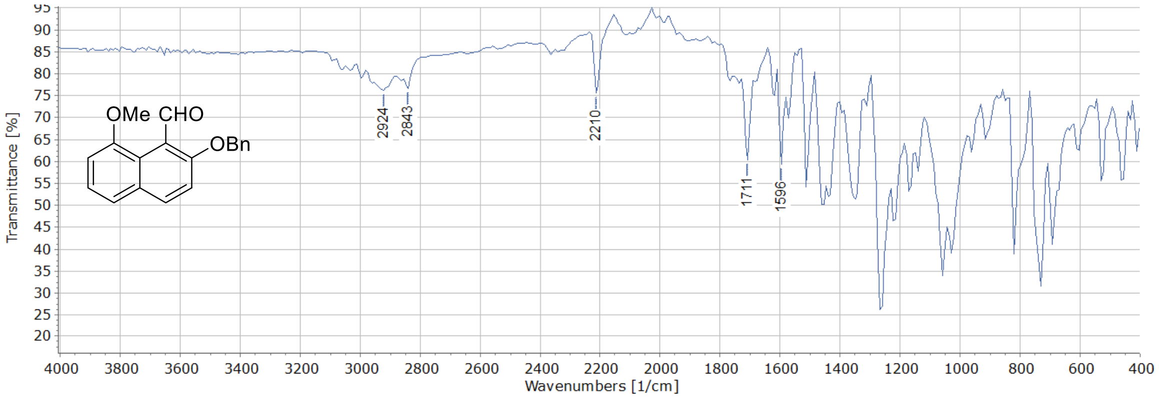

Figure S49. IR spectrum (solid state) of (**25**).

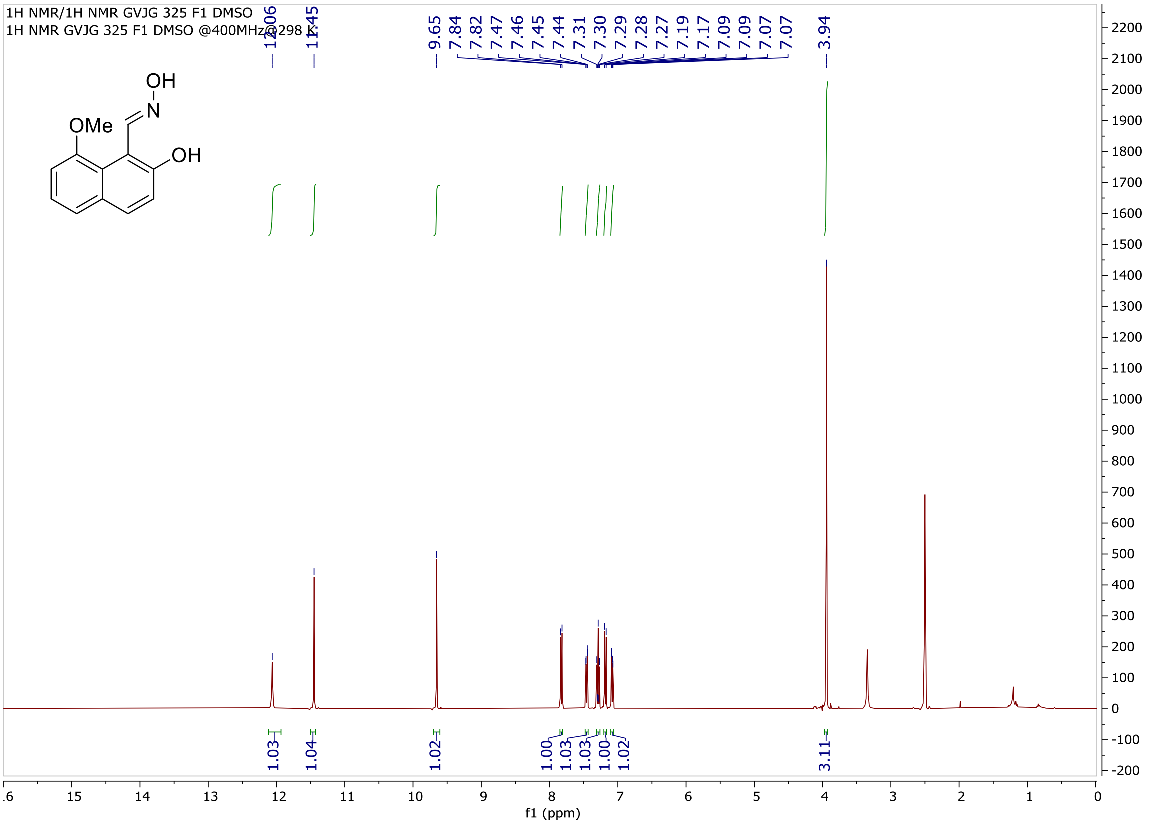

Figure S50. ^{1}H NMR (400 MHz, DMSO-d_6) of (**6**).

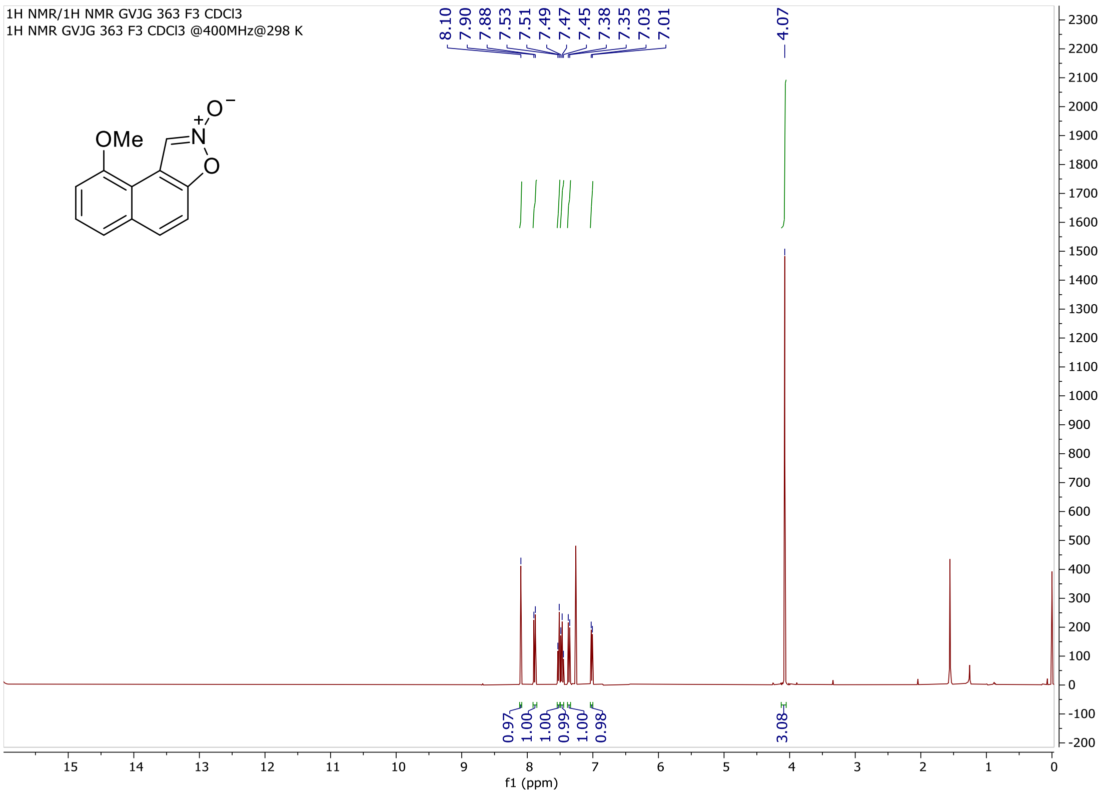

Figure S51. ^{1}H NMR (400 MHz, $CDCl_3$) of (**8**).

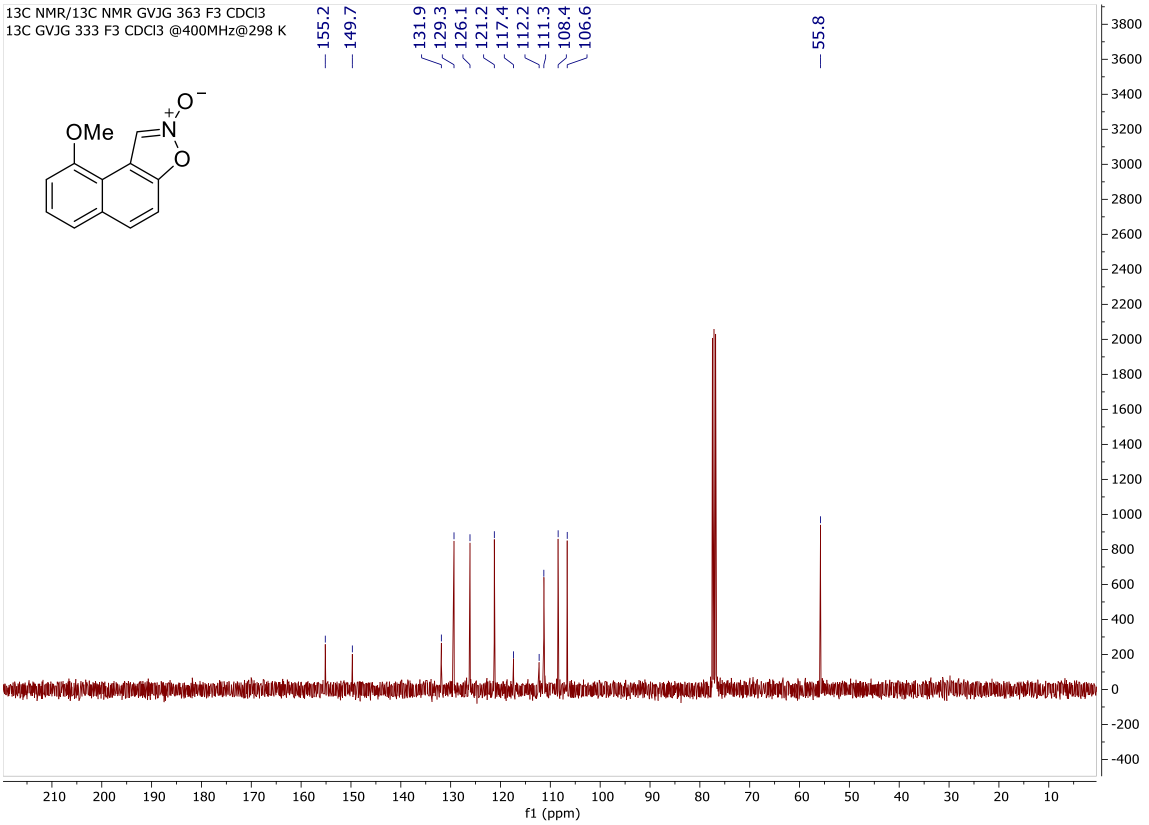

Figure S52. ^{13}C NMR [100.6 MHz, $CDCl_3$] of (**8**).

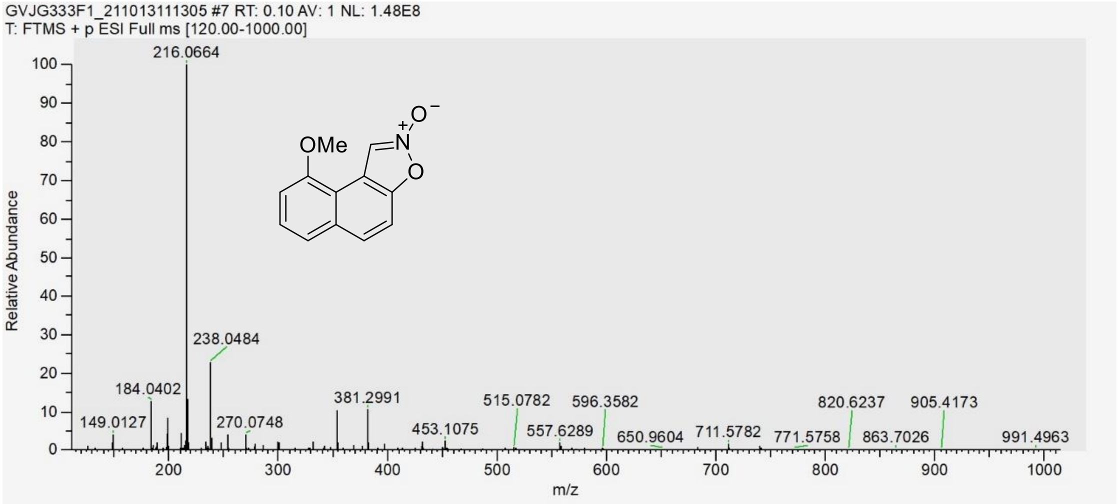

Figure S53. HRMS $[M+H]^+$ of compound (**8**) using 0.1% HCO_2H in MeOH as solvent.

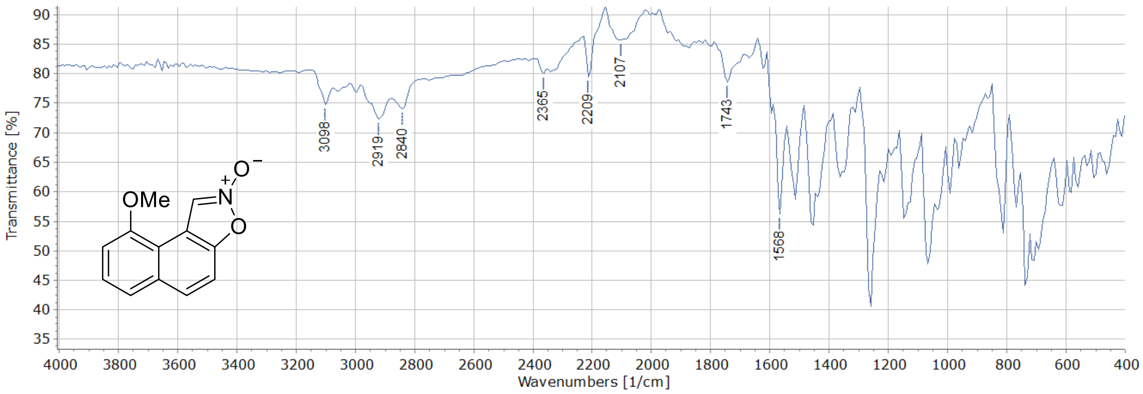

Figure S54. IR spectrum of (**8**).

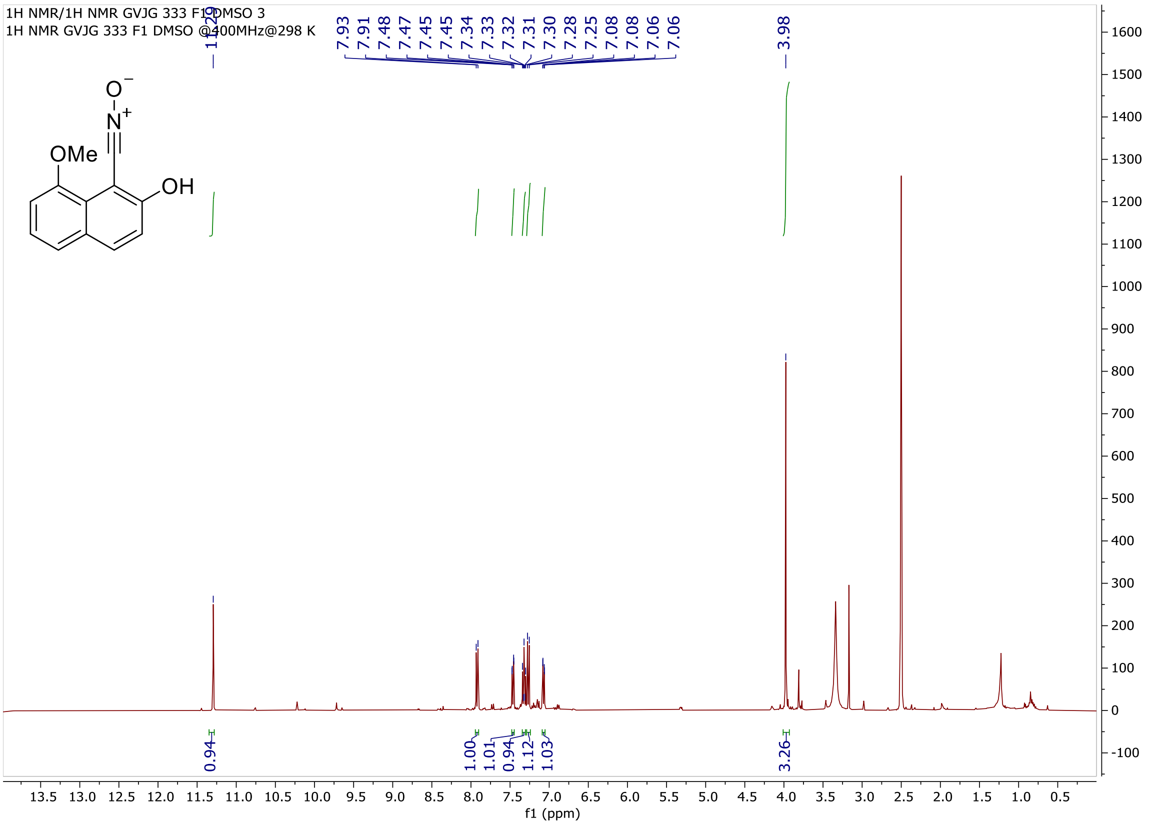

Figure S55. ^{1}H NMR (400 MHz, DMSO-d_6) of (**9**).

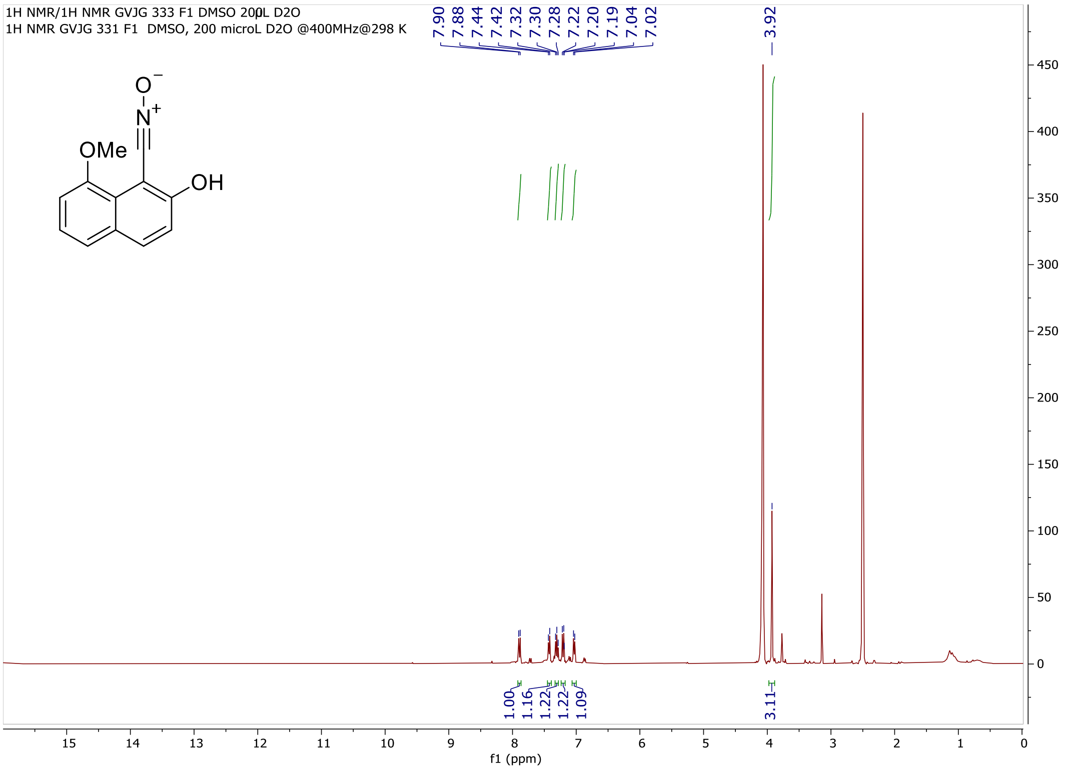

Figure S56. ^{1}H NMR (400 MHz, DMSO-d_6 + D_2O) of (**9**).

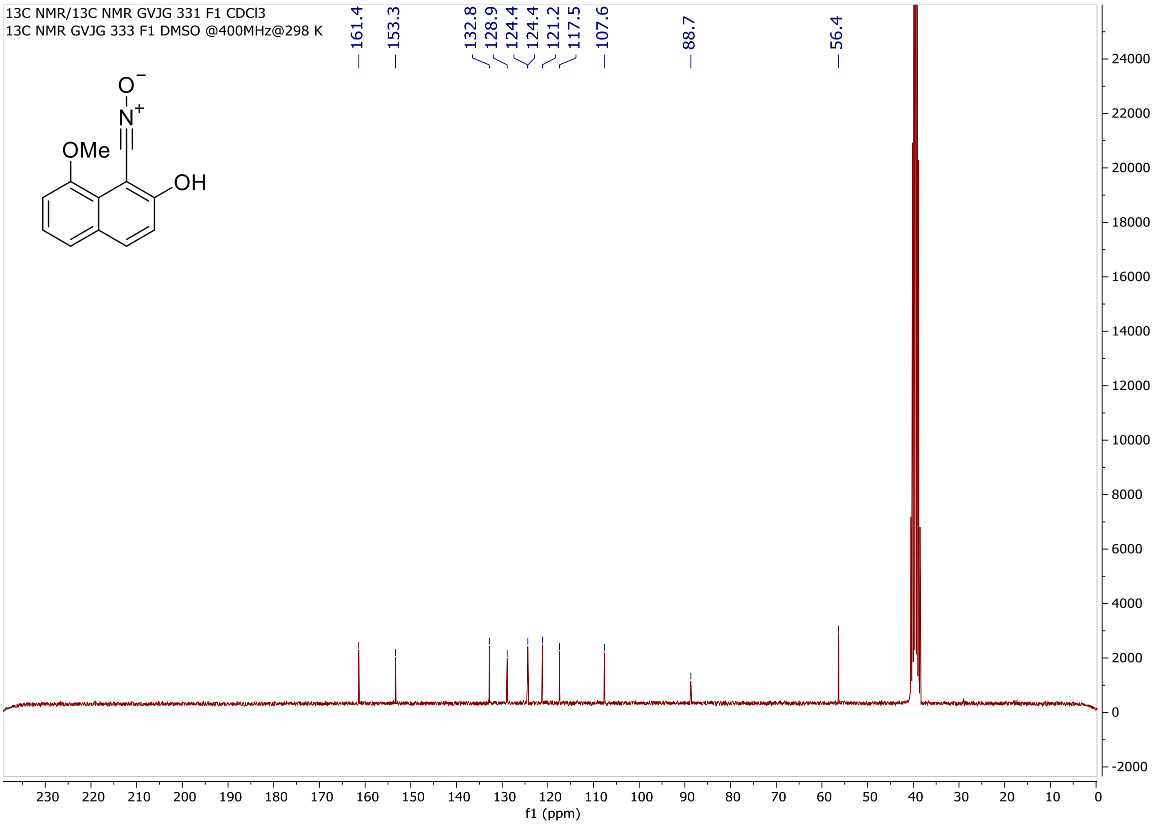

Figure S57. ^{13}C NMR [100.6 MHz, DMSO-d_6] of (**9**).

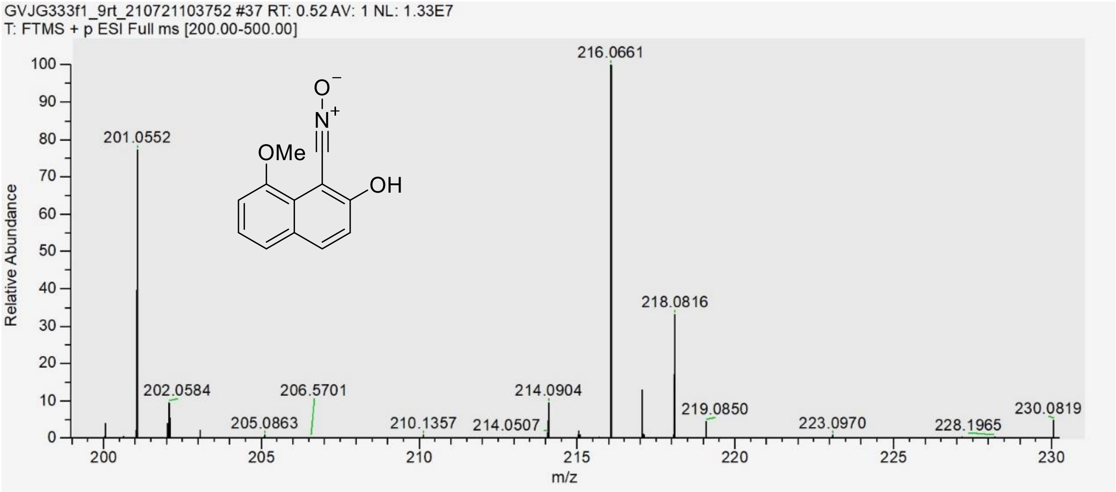

Figure S58. HRMS [M+H]$^+$ of compound (**9**) in DMSO-d_6 using 0.1% HCO_2H in MeOH as solvent.

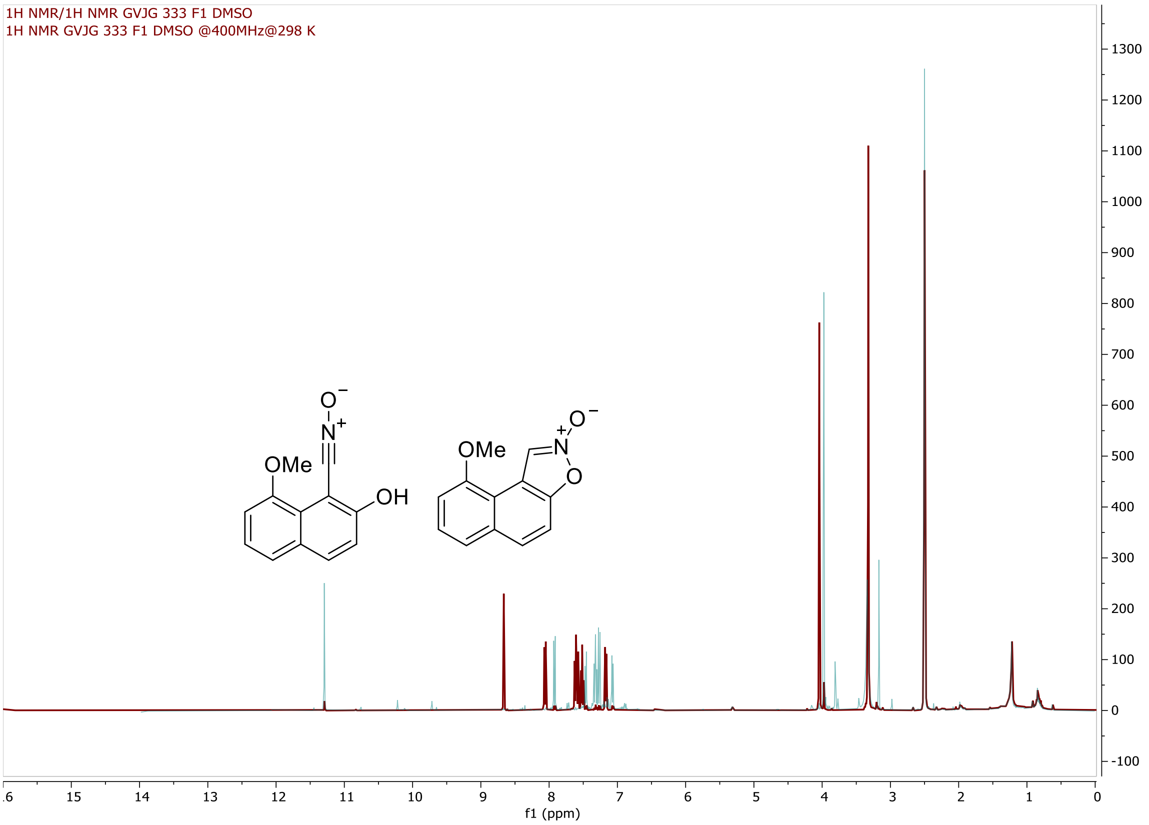

Figure S59. ^{1}H NMR (400 MHz, DMSO-d_6) of nitrile oxide (**9**) and isoxazole-2-oxide (**8**), superimposed.

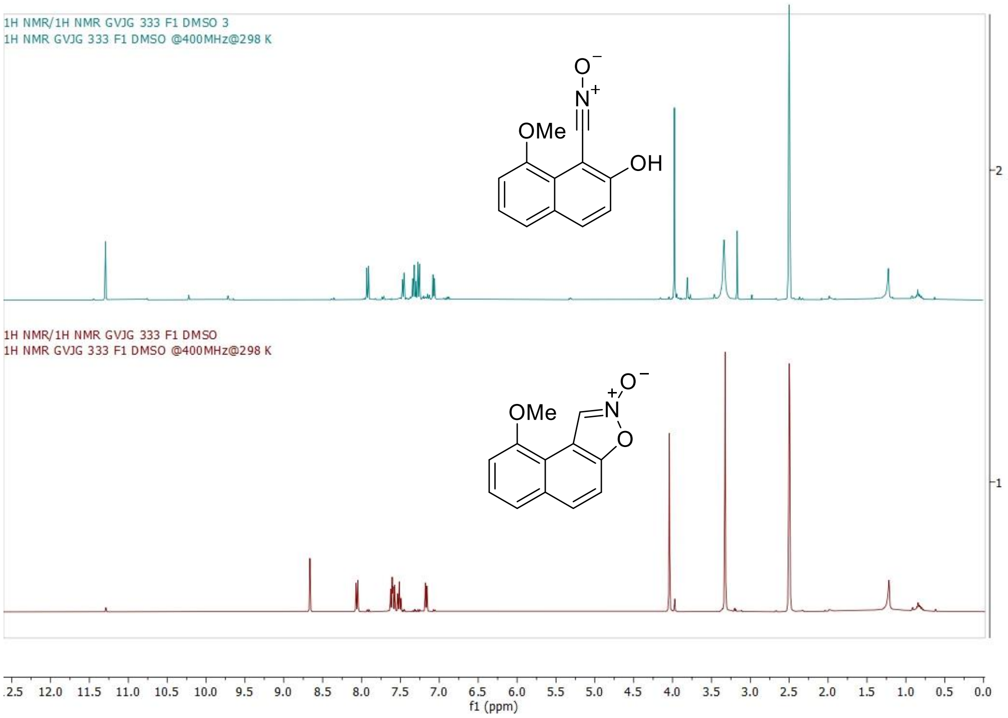

Figure S60. ^{1}H NMR (400 MHz, DMSO-d_6) of nitrile oxide (**9**) and isoxazole-2-oxide (**8**), stacked.

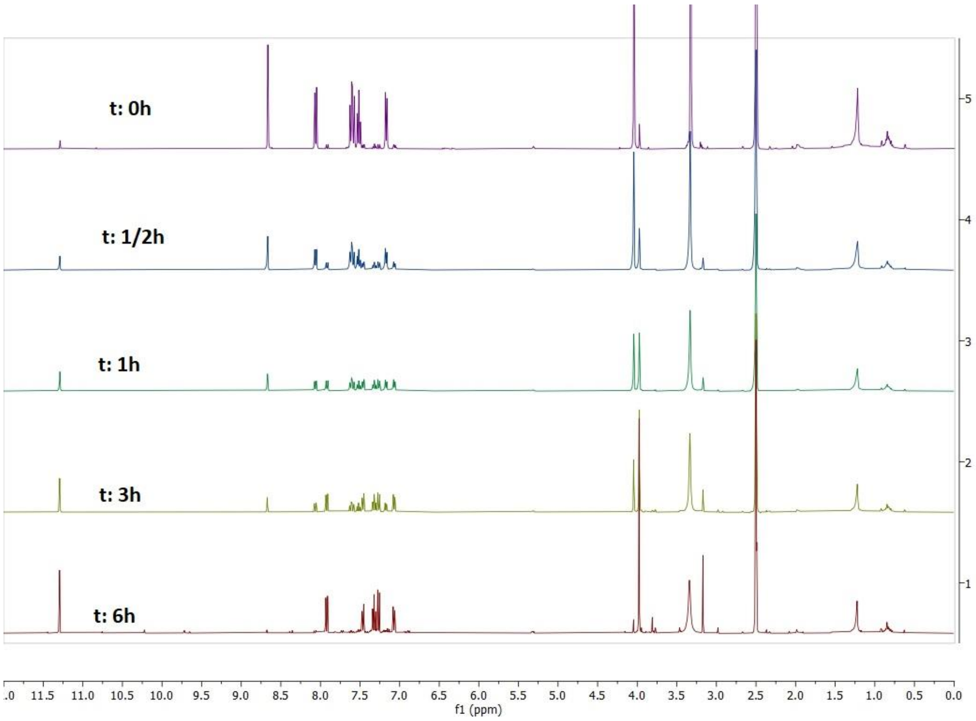

Figure S61. ^{1}H NMR (400 MHz, DMSO-d_6) time-course experiment of the ring opening of isoxazole-2-oxide (**8**) (top spectra) to nitrile oxide (**9**) (bottom spectra).

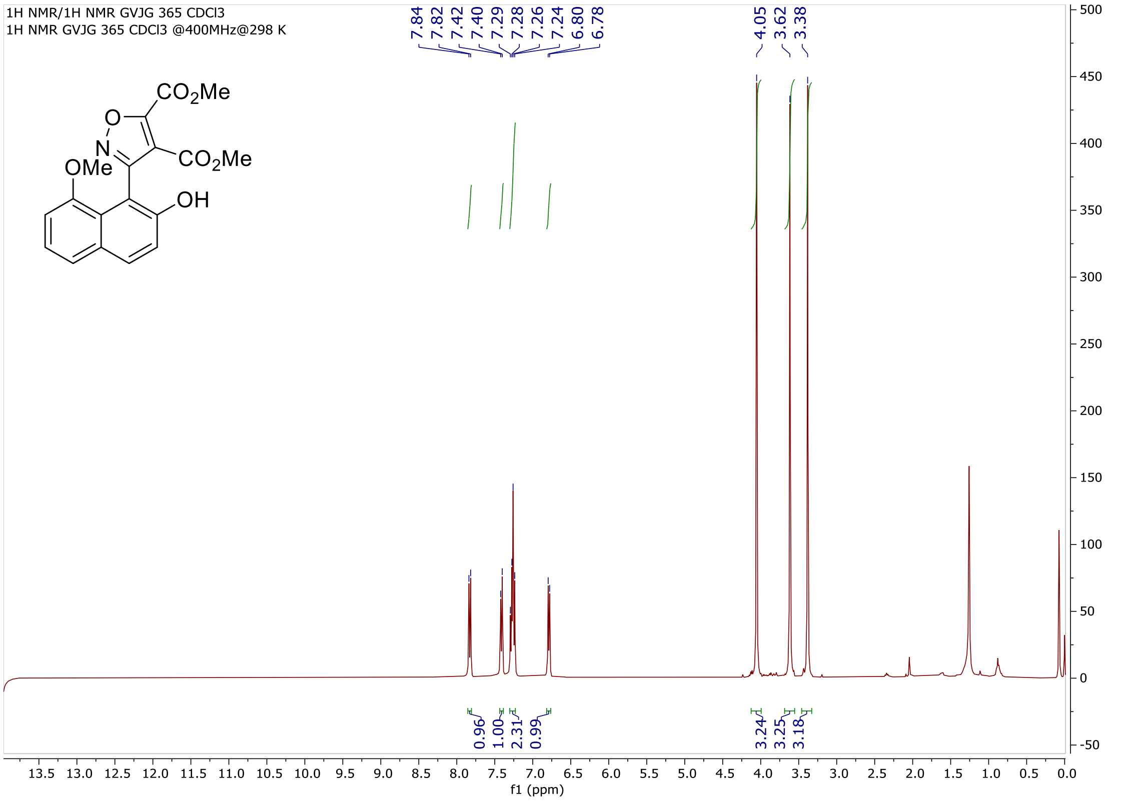

Figure S62. ^{1}H NMR (400 MHz, $CDCl_3$) of (**10a**).

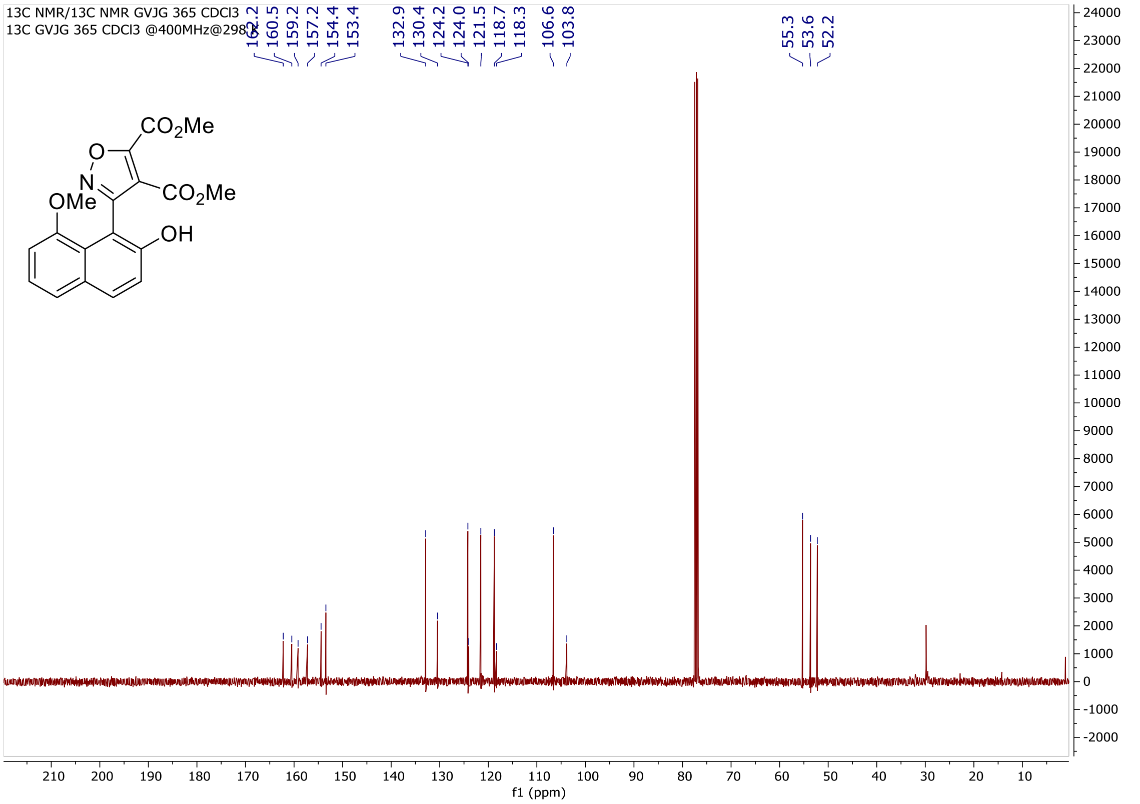

Figure S63. ^{13}C NMR (100.6 MHz, $CDCl_3$) of (**10a**).

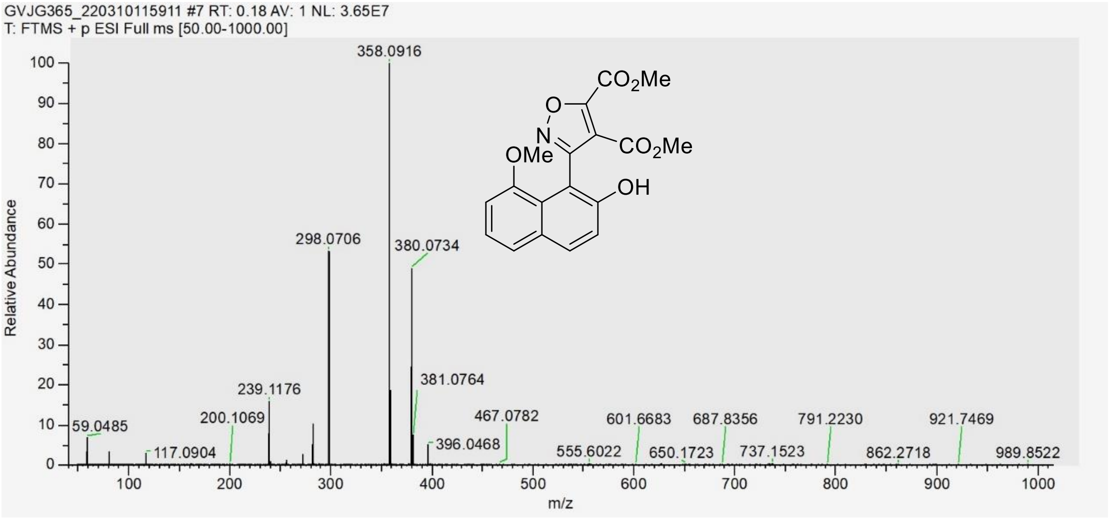

Figure S64. HRMS $[M+H]^+$ of compound (**10a**) using 0.1% HCO_2H in MeOH as solvent.

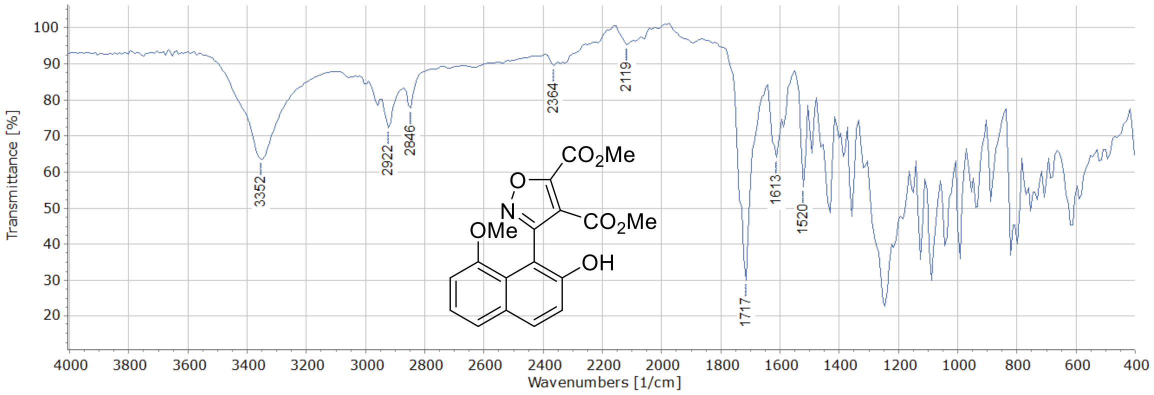

Figure S65. IR spectrum (solid) of (**10a**).

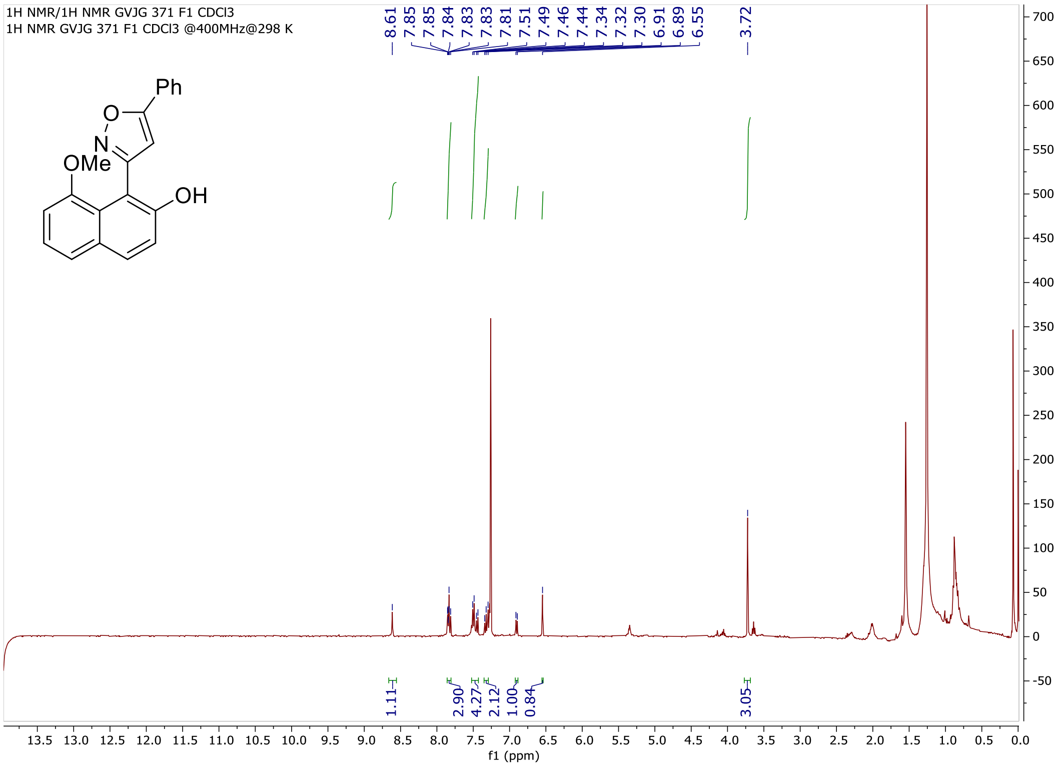

Figure S66. ^{1}H NMR (400 MHz, $CDCl_3$) of (**10b**).

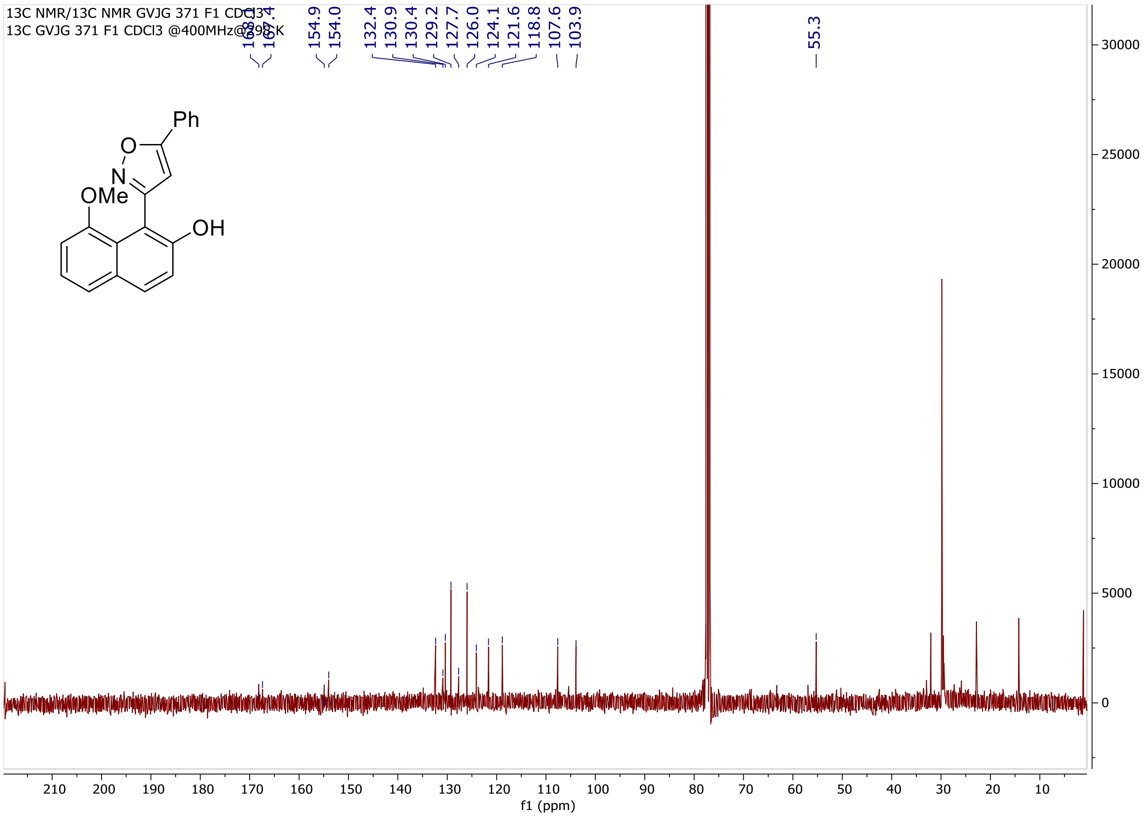

Figure S67. ^{13}C NMR (100.6 MHz, $CDCl_3$) of (**10b**).

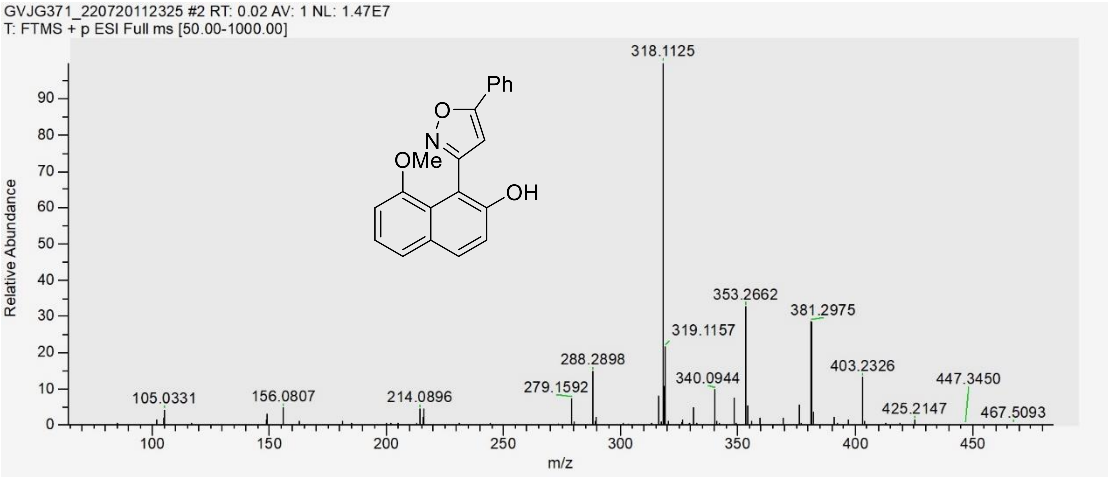

Figure S68. HRMS $[M+H]^+$ of compound (**10b**) using 0.1% HCO_2H in MeOH as solvent.

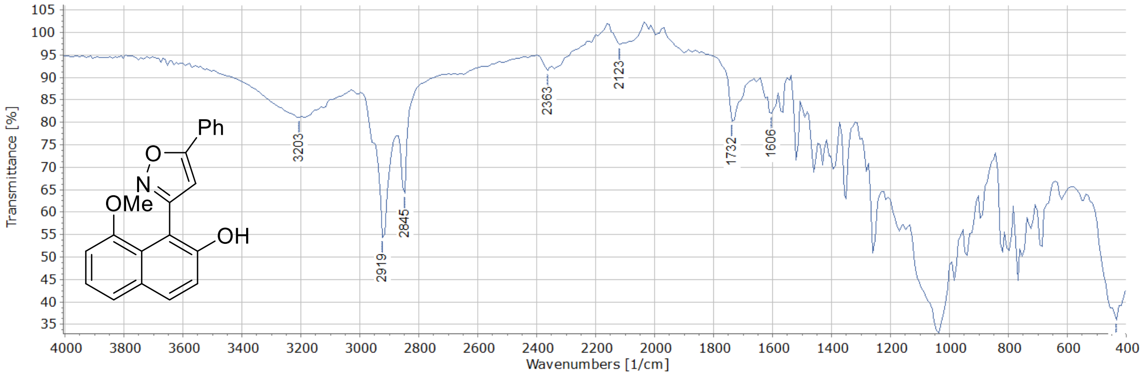

Figure S69. IR spectrum (solid) of (**10b**).

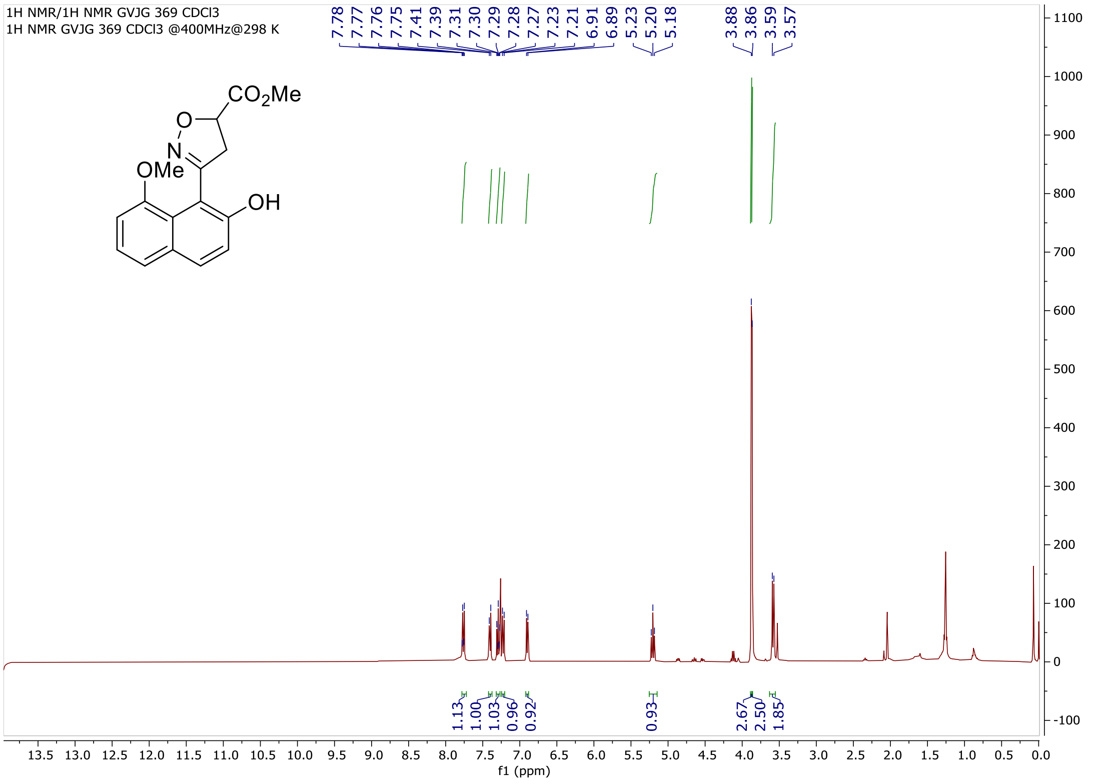

Figure S70. ^{1}H NMR (400 MHz, $CDCl_3$) of (**10c**).

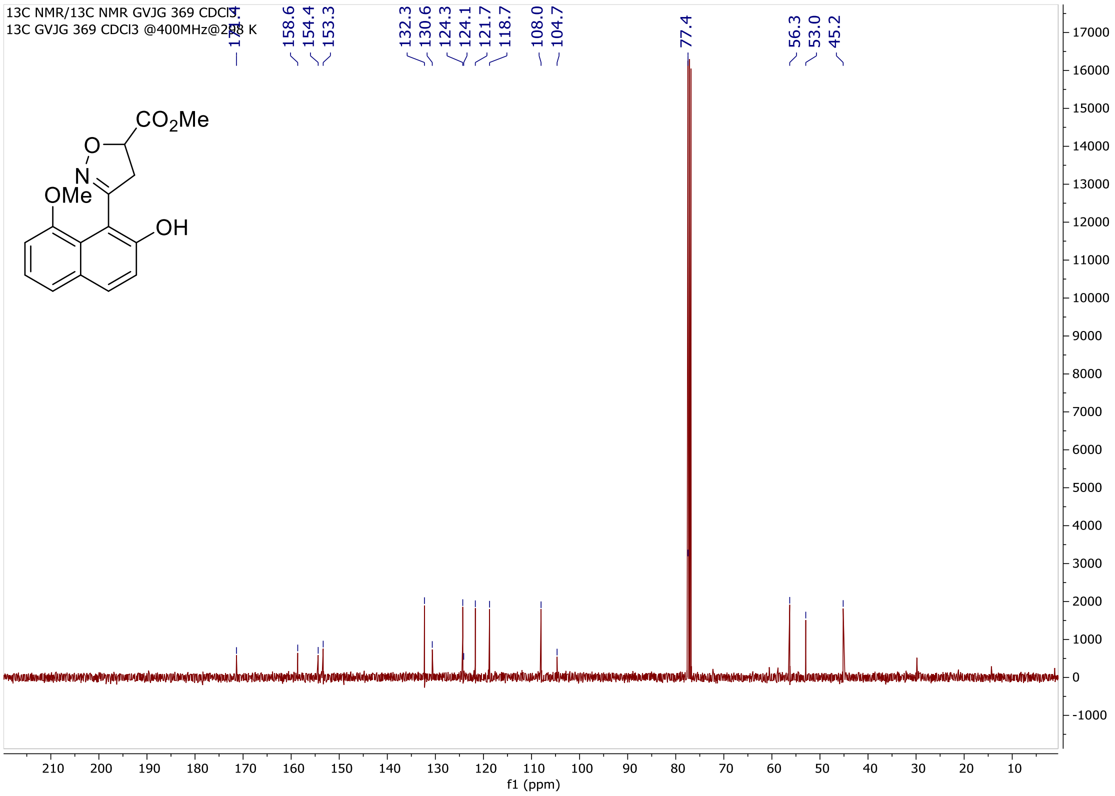

Figure S71. ^{13}C NMR (100.6 MHz, $CDCl_3$) of (**10c**).

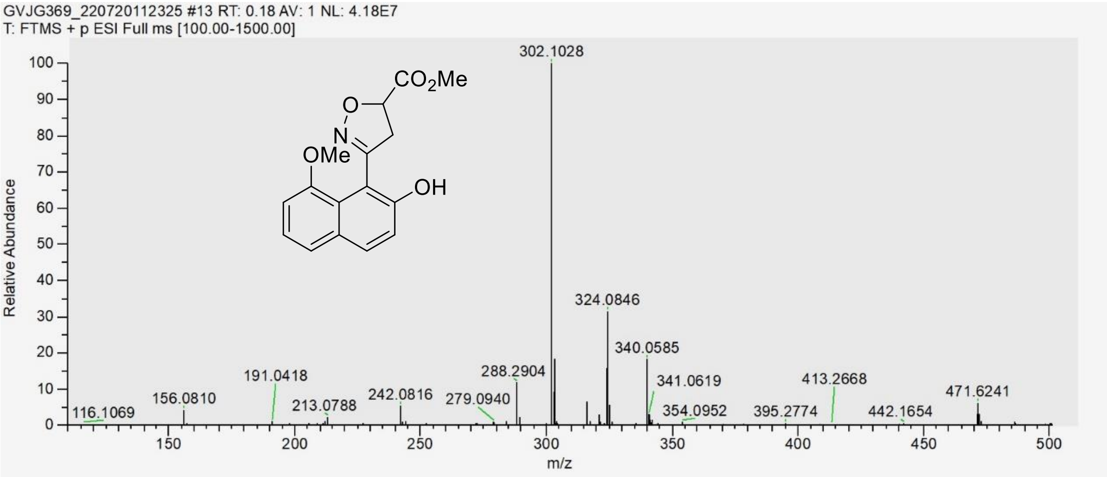

Figure S72. HRMS $[M+H]^+$ of compound (**10c**) using 0.1% HCO_2H in MeOH as solvent.

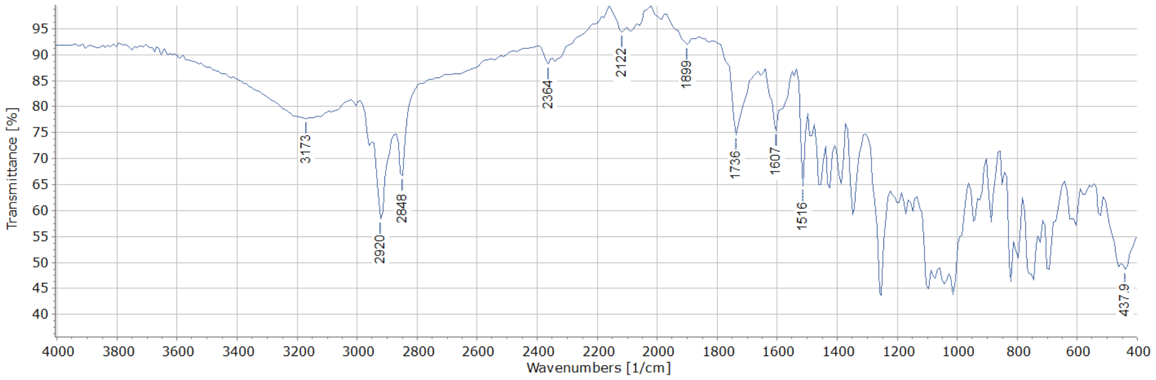

Figure S73. IR spectrum (solid) of (**10c**).

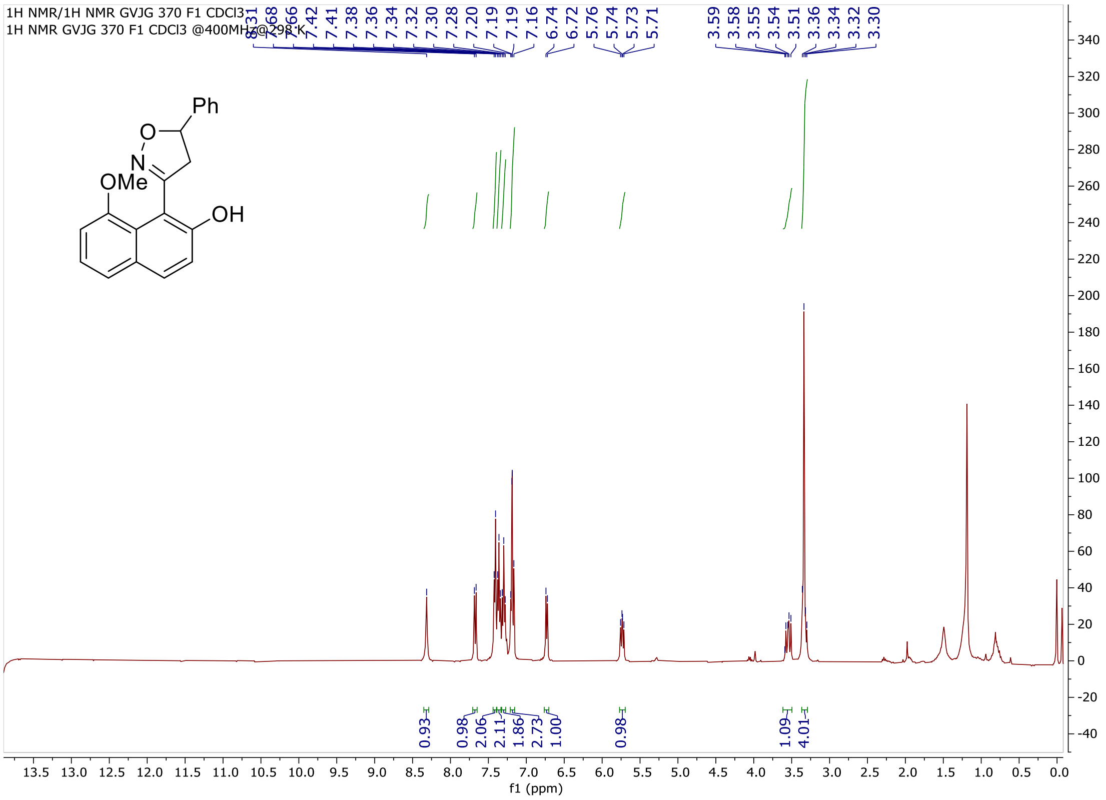

Figure S74. ^{1}H NMR (400 MHz, $CDCl_3$) of (**10d**).

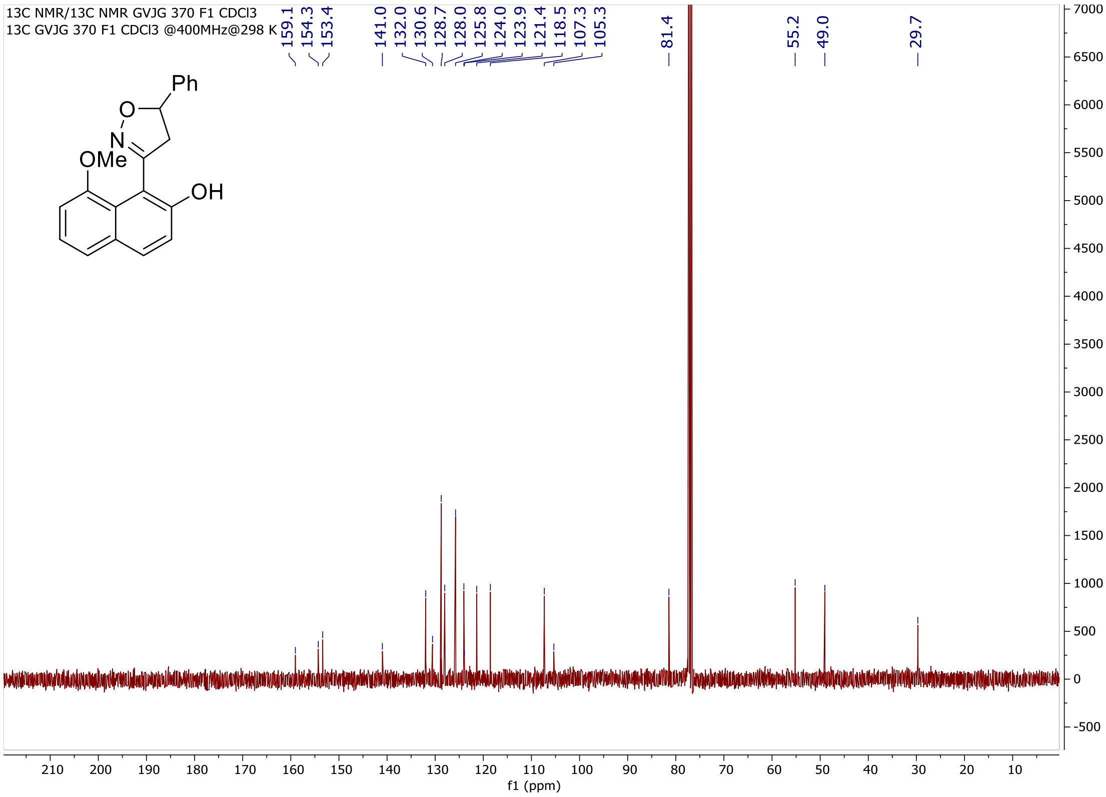

Figure S75. ^{13}C NMR (100.6 MHz, $CDCl_3$) of (**10d**).

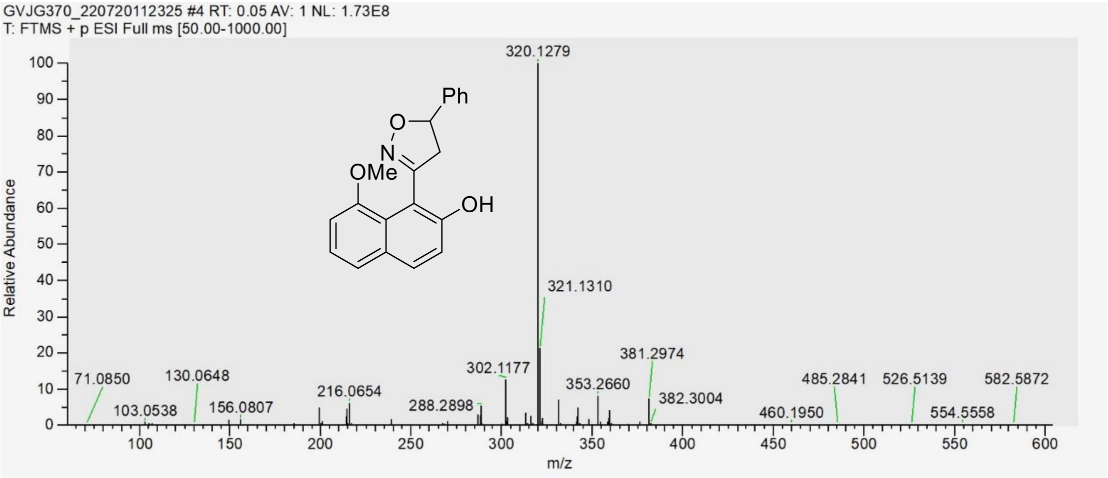

Figure S76. HRMS [M+H]+ of compound (**10d**) using 0.1% HCO_2H in MeOH as solvent.

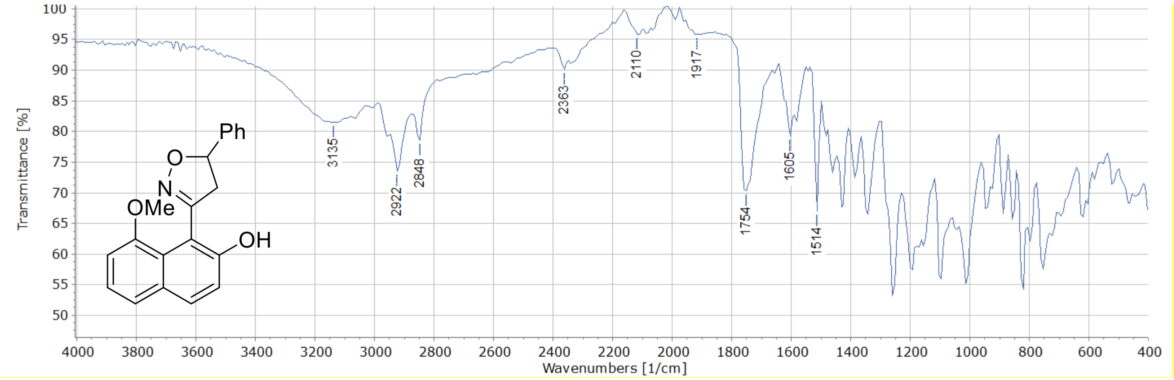

Figure S77. IR spectrum (solid) of (**10d**).
